# Fully automated closed-loop insulin delivery in adults with type 2 diabetes: an open-label, single-center, randomized crossover trial

**DOI:** 10.1038/s41591-022-02144-z

**Published:** 2023-01-11

**Authors:** Aideen B. Daly, Charlotte K. Boughton, Munachiso Nwokolo, Sara Hartnell, Malgorzata E. Wilinska, Alina Cezar, Mark L. Evans, Roman Hovorka

**Affiliations:** 1 Wellcome-MRC Institute of Metabolic Science, Addenbrooke’s Hospital, Cambridge, UK; 2 Cambridge University Hospitals NHS Foundation Trust, Wolfson Diabetes and Endocrine Clinic, Cambridge, UK

**Keywords:** Type 2 diabetes, Diabetes complications

## Abstract

In adults with type 2 diabetes, the benefits of fully closed-loop insulin delivery, which does not require meal bolusing, are unclear. In an open-label, single-center, randomized crossover study, 26 adults with type 2 diabetes (7 women and 19 men; (mean ± s.d.) age, 59 ± 11 years; baseline glycated hemoglobin (HbA1c), 75 ± 15 mmol mol^−1^ (9.0% ± 1.4%)) underwent two 8-week periods to compare the CamAPS HX fully closed-loop app with standard insulin therapy and a masked glucose sensor (control) in random order, with a 2-week to 4-week washout between periods. The primary endpoint was proportion of time in target glucose range (3.9–10.0 mmol l^−1^). Analysis was by intention to treat. Thirty participants were recruited between 16 December 2020 and 24 November 2021, of whom 28 were randomized to two groups (14 to closed-loop therapy first and 14 to control therapy first). Proportion of time in target glucose range (mean ± s.d.) was 66.3% ± 14.9% with closed-loop therapy versus 32.3% ± 24.7% with control therapy (mean difference, 35.3 percentage points; 95% confidence interval (CI), 28.0–42.6 percentage points; *P* < 0.001). Time > 10.0 mmol l^−1^ was 33.2% ± 14.8% with closed-loop therapy versus 67.0% ± 25.2% with control therapy (mean difference, −35.2 percentage points; 95% CI, −42.8 to −27.5 percentage points; *P* < 0.001). Mean glucose was lower during the closed-loop therapy period than during the control therapy period (9.2 ± 1.2 mmol l^−1^ versus 12.6 ± 3.0 mmol l^−1^, respectively; mean difference, −3.6 mmol l^−1^; 95% CI, −4.6 to −2.5 mmol l^−1^; *P* < 0.001). HbA1c was lower following closed-loop therapy (57 ± 9 mmol mol^−1^ (7.3% ± 0.8%)) than following control therapy (72 ± 13 mmol mol^−1^ (8.7% ± 1.2%); mean difference, −15 mmol mol^−1^; 95% CI, −11 to −20 mmol l^−1^ (mean difference, −1.4%; 95% CI, −1.0 to −1.8%); *P* < 0.001). Time < 3.9 mmol l^−1^ was similar between treatments (a median of 0.44% (interquartile range, 0.19–0.81%) during the closed-loop therapy period versus a median of 0.08% (interquartile range, 0.00–1.05%) during the control therapy period; *P* = 0.43). No severe hypoglycemia events occurred in either period. One treatment-related serious adverse event occurred during the closed-loop therapy period. Fully closed-loop insulin delivery improved glucose control without increasing hypoglycemia compared with standard insulin therapy and may represent a safe and efficacious method to improve outcomes in adults with type 2 diabetes. This study is registered with ClinicalTrials.gov (NCT04701424).

## Abstract (web summary)

In a randomized, crossover trial in adults with type 2 diabetes, fully closed-loop insulin delivery increased time in target glucose range compared with standard insulin therapy, without increasing hypoglycemia.

## Main

The burden of type 2 diabetes is far reaching, with an estimated 415 million people affected worldwide^1^ and an estimated annual global health expenditure of US $760 billion (ref. ^2^). Intensive glycemic management to achieve the target glycated hemoglobin (HbA1c) value of less than 7% (53 mmol mol^−1^) is supported by good-quality evidence^3^, but glucose control remains inadequate globally^2^.

In the initial stages of the disease, most people with type 2 diabetes require a combination of lifestyle interventions and pharmacological therapy, benefiting from the availability of an increasing number of oral antihyperglycemic agents^4^. Progressive beta-cell depletion and adoption of the early intensive glycemic-control paradigm mandate initiation of insulin therapy^5^, which improves glycemic control but increases the risk of hypoglycemia^6^.

Closed-loop insulin delivery offers a new, glucose-responsive method of glucose control and comprises a continuous glucose monitor, an insulin pump and a control algorithm that automatically modulates subcutaneous insulin delivery^7^. Hybrid closed-loop systems, with a requirement for carbohydrate announcement and prandial insulin boluses, are commercially available for people with type 1 diabetes^7–9^.

Fully closed-loop systems, which are more convenient and have no requirement for user input at meal times, have previously been shown to improve glucose control in people with type 2 diabetes in the inpatient setting^10–12^, and in those requiring dialysis over a period of 20 days in an outpatient setting^13^. However, benefits over longer periods of several months are unclear. Here we proposed that fully closed-loop insulin delivery in people with type 2 diabetes may improve glycemic control compared with standard insulin therapy, without increasing the risk of hypoglycemia over an 8-week period of unrestricted living.

## Results

### Participants

**Fig. 1:**
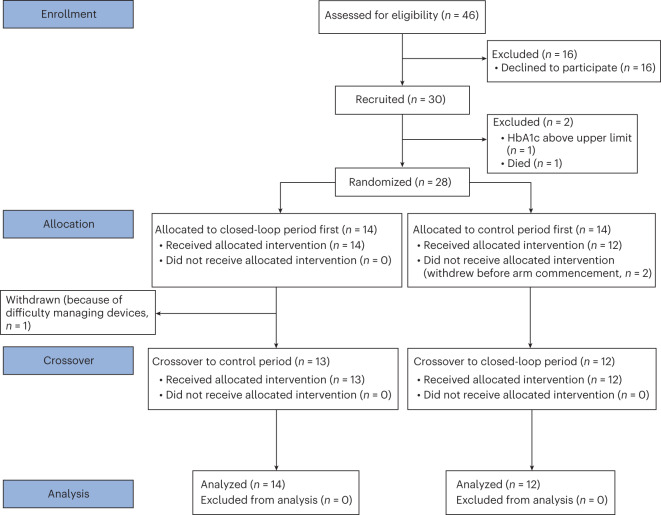
Participant flow. Overview of the participant flow.

From 16 December 2020 to 24 November 2021, a total of 46 people were screened, and 30 participants were recruited. One participant died before randomization, and one participant was ineligible because of an HbA1c level of >108 mmol mol^−1^ (12%). The flow of participants through the trial is shown in Fig. 1. Twenty-eight participants were randomized to two groups (14 to closed-loop therapy first and 14 to control therapy first). Two participants withdrew before starting their first study period (both randomized to control therapy first). One participant stopped the study early during the first intervention period (closed-loop) because of difficulty managing the devices. This participant completed a minimum of 48 h. Therefore, data for this period were included in the analysis. Baseline characteristics of the study population are shown in Table 1, and baseline diabetes regimen details are shown in Supplementary Table 1.

**Table 1:** Baseline characteristics

Characteristic	Overall^a^ (*n* = 26)	Closed-loop therapy first (*n* = 14)	Control therapy first^a^ (*n* = 12)
Age (years)	59 (11)	59 (12)	59 (10)
Female sex, *n* out of total *n* (%)	7 out of 26 (27)	3 out of 14 (21)	4 out of 12 (33)
Ethnic origin, *n* (%)			
White	25 (96)	14 (100)	11 (92)
Black	0 (0)	0 (0)	0 (0)
Asian	1 (4)	0 (0)	1 (8)
BMI (kg m^−^^2^)	35.3 (8.6)	37.4 (9.1)	33.0 (7.7)
HbA1c (mmol mol^−1^)	75 (15)	76 (12)	74 (19)
HbA1c (%)	9.0 (1.4)	9.1 (1.1)	9.0 (1.8)
Duration of diabetes (years)	17.5 (8.2)	17.2 (7.3)	17.8 (9.5)
Duration of insulin therapy (years)	8.5 (6.9)	7.9 (7.0)	9.3 (7.0)
Total daily insulin dose (U kg^−1^)	0.70 (0.54–1.31)	0.69 (0.38–1.32)	0.83 (0.58–1.36)
Prior CGM or flash glucose monitor use, *n* (%)	3 (12)	1 (4)	2 (8)
Charlson comorbidity index	4 (2–5)	4 (2–6)	4 (2–4)

Data are presented as mean (s.d.) or median (IQR) unless stated otherwise. No significant differences between the groups were observed at baseline. For the Charlson comorbidity index, the higher the comorbidity index, the greater the burden of comorbidities. CGM, continuous glucose monitor.

^a^Excludes two participants who were randomized to the control therapy period first but were withdrawn before starting the allocated treatment.

### Primary endpoint

**Extended Data Fig. 1:**
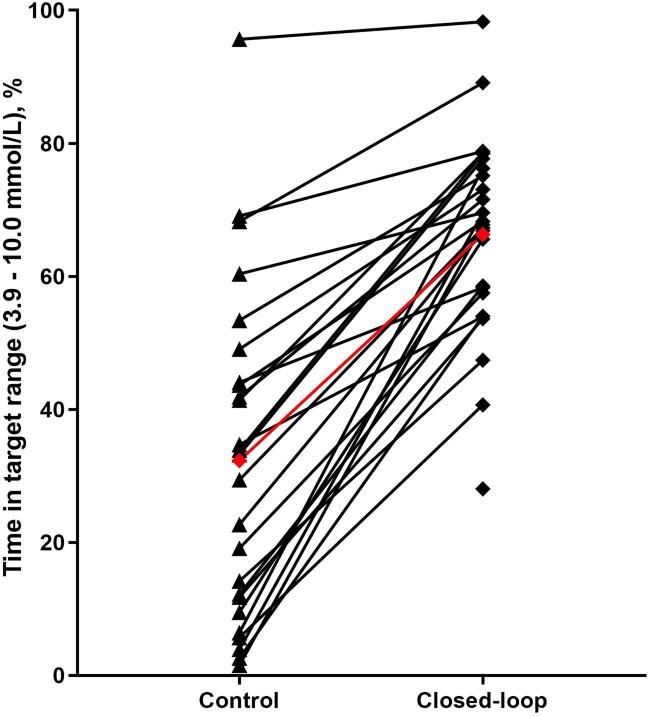
Individual participants’ time spent in target range 3.9–10.0 mmol/L (n = 26). Overall mean is shown in red.

The primary and secondary endpoints, which were calculated using data from all randomized participants for whom at least 48 h of data were collected in at least one study period (*n* = 26), are presented in Table 2. The primary endpoint, which was the proportion of time with sensor glucose in the target glucose range of 3.9–10.0 mmol l^−1^, was greater during closed-loop insulin therapy than during standard insulin therapy ((mean ± s.d.) 66.3% ± 14.9% versus 32.3% ± 24.7%, respectively), with a mean difference of 35.3 percentage points (95% confidence interval (CI), 28.0–42.6 percentage points; *P* < 0.001). The difference in the proportion of time that glucose was in the target range in the closed-loop therapy period compared with the control therapy period for individual participants is shown in Extended Data Fig. 1.

**Table 2:** Primary and secondary endpoints during the closed-loop and control therapy periods

Endpoint	Closed-loop (*n* = 26)	Control (*n* = 25)^a^	Mean difference (95% CI for treatment difference^b^)	*P* value
Primary endpoint				
Proportion of time with glucose 3.9–10.0 mmol l^−1^ (%)	66.3 (14.9)	32.3 (24.7)	35.3 (28.0 to 42.6)	<0.001
Key endpoints^c^				
Proportion of time with glucose > 10.0 mmol l^−1^ (%)	33.2 (14.8)	67.0 (25.2)	−35.2 (−42.8 to −27.5)	<0.001
Mean glucose (mmol l^−1^)	9.2 (1.2)	12.6 (3.0)	−3.6 (−4.6 to −2.5)	<0.001
HbA1c (mmol mol^−1^)	57 (9)	72 (13)	−15 (−20 to −11)	<0.001
HbA1c (%)	7.3 (0.8)	8.7 (1.2)	−1.4 (−1.8 to −1.0)	<0.001
Proportion of time with glucose < 3.9 mmol l^−1^ (%)	0.44 (0.19–0.81)	0.08 (0.00–1.05)	−0.10 (−0.36 to 0.16)	0.43
Secondary endpoints				
Proportion of time with glucose > 16.7 mmol l^−1^ (%)	1.8 (0.6–3.3)	12.5 (3.6–31.3)	NA	NA
Proportion of time with glucose > 20.0 mmol l^−1^ (%)	0.2 (0.0–0.5)	3.2 (0.2–9.7)	NA	NA
Proportion of time with glucose < 3.0 mmol l^−1^ (%)	0.04 (0.01–0.08)	0.03 (0.00–0.32)	NA	NA
s.d. of glucose (mmol l^−1^)	3.0 (0.8)	3.4 (1.0)	NA	NA
Coefficient of variation of glucose (%)	32.2 (5.7)	27.7 (8.5)	NA	NA
Total daily insulin dose (U per day)	108 (73–188)	84 (54–129)	NA	NA
Total daily insulin dose (U kg^−1^ per day)	0.90 (0.72–1.63)	0.71 (0.56–1.26)	NA	NA
Proportion of time with sensor glucose availability (%)	98.1 (96.8–98.5)	92.6 (89.8–98.0)	NA	NA
Proportion of time spent with closed-loop active (%)	92.3 (87.6–96.4)	NA	NA	NA

Data are presented as mean (s.d.) or median (IQR). Endpoints are calculated data from all randomized participants for whom at least 48 h of sensor glucose data were collected in at least one period. Glucose data are based on sensor glucose measurements. A two-sample *t*-test on paired differences was used to compare normally distributed variables. For highly skewed residuals for key and secondary endpoints, a transformation (windsorization) was used. All *P* values are two-tailed, and *P* < 0.05 was considered statistically significant. s.d. of glucose is a measure of glucose variability. NA, not applicable.

^a^One participant randomized to closed-loop therapy first did not continue to control therapy.

^b^Closed-loop minus control.

^c^Tested in hierarchy to control the type 1 error using the fixed-sequence method. A gatekeeping strategy was used, in which the primary endpoint was tested first and, if passing the significance testing, other key endpoints were tested in order. If a nonsignificant result was encountered, formal statistical hypothesis testing was terminated, and analysis of any key endpoints below the one in question (any that were lower in the hierarchy) was considered exploratory.

### Key secondary endpoints

**Fig. 2:**
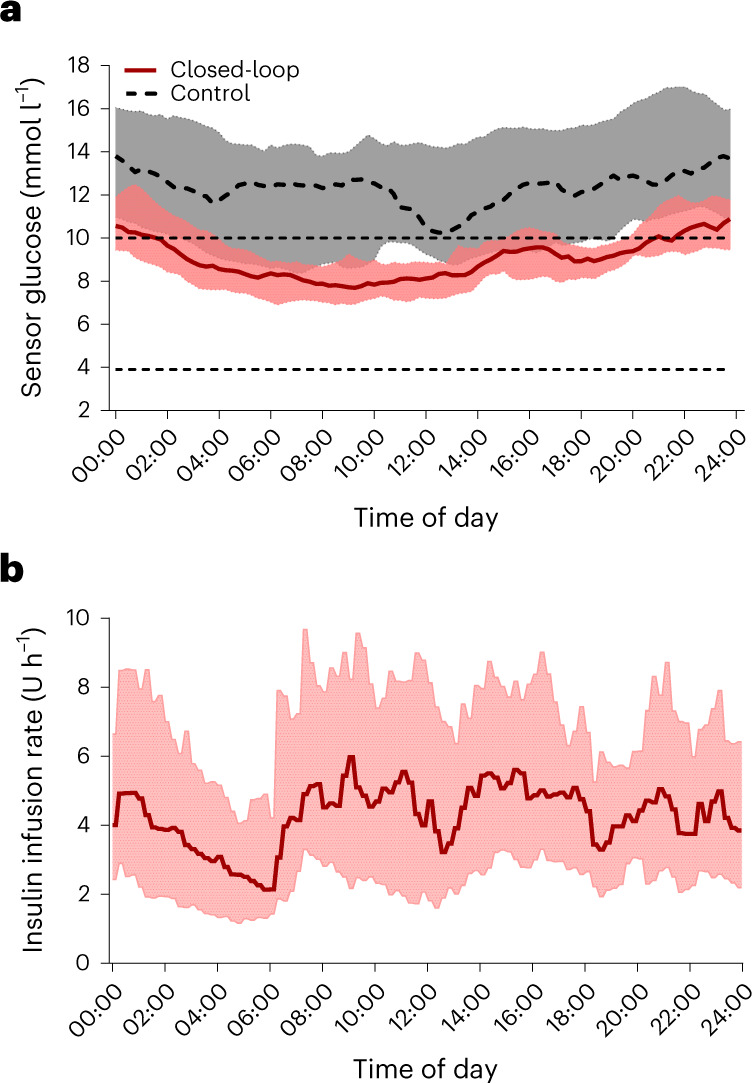
Glucose control during closed-loop and control periods. **a**, Median sensor glucose measurements during closed-loop insulin delivery and control insulin therapy (the patients' usual therapy). Red and gray shaded areas, IQR for each treatment. The values are reported during a 24-h period from midnight to midnight. Black horizontal dashed lines, lower and upper limits of the glucose target range of 3.9–10.0 mmol l^−1^. **b**, Median amount of algorithm-directed insulin delivery during the closed-loop intervention. Shaded area, IQR.

The proportion of time with sensor glucose of >10.0 mmol l^−1^ was lower during the closed-loop therapy period than during the control therapy period ((mean ± s.d.) 33.2% ± 14.8% versus 67.0% ± 25.2%, respectively; mean difference, −35.2 percentage points; 95% CI, −42.8 to −27.5 percentage points; *P* < 0.001). Mean glucose was also lower during the closed-loop therapy period than during the control therapy period ((mean ± s.d.) 9.2 ± 1.2 mmol l^−1^ versus 12.6 ± 3.0 mmol l^−1^, respectively; mean difference, −3.6 mmol l^−1^; 95% CI, −4.6 to −2.5 mmol l^−1^; *P* < 0.001). HbA1c was significantly lower after the closed-loop therapy period ((mean ± s.d.) 57 ± 9 mmol mol^−1^ (7.3% ± 0.8%)) than after the control therapy period (72 ± 13 mmol mol^−1^ (8.7% ± 1.2%); mean-adjusted difference, −15 mmol mol^−1^; 95% CI, −20 to −11 mmol l^−1^; mean difference, −1.4 percentage points; 95% CI, −1.8 to −1.0 percentage points; *P* < 0.001). Time spent in hypoglycemia (<3.9 mmol l^−1^) was low and similar between treatment periods (a median of 0.44% (interquartile range (IQR), 0.19–0.81%) during closed-loop therapy versus a median of 0.08% (IQR, 0.00–1.05%) during control therapy; *P* = 0.43). Figure 2 shows the 24-h sensor glucose and closed-loop insulin profiles.

Formal statistical hypothesis testing was terminated after time spent in hypoglycemia (3.9 mmol l^−1^) was found not to be significantly different between treatment periods (Methods). Therefore, analysis of other secondary endpoints is considered exploratory.

### Other secondary endpoints

The proportion of time spent in hyperglycemia (>16.7 mmol l^−1^ and >20.0 mmol l^−1^) was lower during closed-loop therapy than during control therapy (for >16.7 mmol l^−1^, a median of 1.8% (IQR, 0.6–3.3%) during the closed-loop therapy period versus a median of 12.5% (IQR, 3.6–31.3%) during the control therapy period; and for >20 mmol l^−1^, a median of 0.2% (IQR, 0.0–0.5%) for the closed-loop therapy period versus a median of 3.2% (IQR, 0.2–9.7%) during the control therapy period). Time spent with glucose < 3.0 mmol l^−1^ was low and similar for both periods (a median of 0.04% (IQR, 0.01–0.08%) during the closed-loop therapy period versus a median of 0.03% (IQR, 0.00–0.32) during the control therapy period). Glucose variability measured by the s.d. of glucose was lower during closed-loop therapy than during control therapy ((mean ± s.d.) 3.0 ± 0.8 versus 3.4 ± 1.0, respectively), but the coefficient of variation was higher during closed-loop therapy than during control therapy ((mean ± s.d.) 32.2% ± 5.7% versus 27.7% ± 8.5%, respectively). Total daily insulin dose was higher during the closed-loop therapy period (median, 0.90 U kg^−1^ per day; IQR, 0.72–1.63 U kg^−1^ per day)) than during the control therapy period (median, 0.71 U kg^−1^ per day; IQR, 0.56–1.26 U kg^−1^ per day).

Per-protocol analysis of the primary endpoint is similar to the intention-to-treat analysis (Supplementary Table 2). Glucose metrics during daytime and nighttime, and by fortnightly periods are shown in Supplementary Tables 3 and 4. The primary and key endpoints by treatment sequence are presented in Supplementary Table 5. There was no period effect for time in target glucose range (*P* = 0.12) or HbA1c (*P* = 0.78).

### Safety

There were no episodes of severe hypoglycemia during either intervention period (Table 3). Eight serious adverse events (SAEs) were reported. Four occurred during the closed-loop therapy period, of which one was study procedure related (hospital admission with abscess at pump cannula site requiring incision and drainage). Two SAEs were reported during the control therapy period, and both were not study related. Details of the individual SAEs are shown in Supplementary Table 6. Eleven adverse events were reported (five during the closed-loop therapy period, five during the control therapy period, and one during the washout period), and six device deficiencies occurred (six during the closed-loop therapy period and none during the control therapy period) but did not lead to an adverse event.

**Table 3:** Adverse events and safety analyses

Adverse event	Overall (*n* = 30)	Prerandomization (*n* = 30)	Closed-loop (*n* = 26)	Control (*n* = 25)	Washout (*n* = 25)
No. of severe hypoglycemic events	0	0	0	0	0
No. (%) of participants with severe hypoglycemic events	0 (0)	0 (0)	0 (0)	0 (0)	0 (0)
No. of SAEs Study related Nonstudy related	8 1 7	2 0 2	4 1 3	2 0 2	0 0 0
No. (%) of participants with SAEs	6 (20)	1 (3)	3 (12)	1 (4)	0 (0)
No. of other adverse events	11	0	5	5	1
No. (%) of participants with adverse events	11 (37)	0 (0)	5 (19)	5 (20)	1 (4)
No. of device deficiencies	6	0	6	0	0
Pump related	4	0	4	0	0
Sensor related	1	0	1	0	0
Smartphone related	1	0	1	0	0
No. (%) of participants with device deficiencies	5 (17)	0 (0)	5 (19)	0 (0)	0 (0)

### Utility

Glucose sensor availability was higher during the closed-loop therapy period than during the control therapy period (a median of 98.1% (IQR, 96.8–98.5%) versus a median of 92.6% (IQR, 89.8–98.0%), respectively). The proportion of time that the closed-loop system was active was high during the closed-loop therapy period (median, 92.3% (IQR, 87.6–96.4%)).

Protocol deviations are shown in Supplementary Table 7. The majority of protocol deviations (25 out of 30) were outside of protocol visits.

### Questionnaire responses

Hypoglycemia confidence scores and PAID (problem areas in diabetes) scores of diabetes distress were similar between interventions (for hypoglycemia confidence score, a median of 3.3 (IQR, 3.2–3.9) for closed-loop therapy versus a median of 3.4 (IQR, 2.9–3.6) for control therapy; for PAID, a median of 22.5 (IQR, 6.9–55.6) for closed-loop therapy versus a median of 20.0 (IQR, 11.3–39.7) for control therapy). Hypoglycemia worry score was higher following closed-loop therapy than following control therapy (a median of 15.0 (IQR, 6.5–20.0) versus a median of 9.5 (IQR, 6.0–21.0), respectively), indicating greater fear of hypoglycemia during closed-loop therapy than during control therapy (Supplementary Table 8). Responses to the closed-loop experience questionnaire are shown in Supplementary Table 9.

## Discussion

The present study shows that fully closed-loop insulin delivery is a safe and effective approach to improve glycemic control in people with type 2 diabetes during 8 weeks of use in the outpatient setting. The closed-loop system improved the proportion of time spent in target glucose range by 35 percentage points (an additional 8 h per day), and reduced mean glucose, compared with standard insulin therapy, without increasing the time spent in hypoglycemia (Table 2).

The results of this study build on evidence from a feasibility study evaluating fully closed-loop therapy in people with type 2 diabetes and end-stage renal failure on dialysis^13^. Here, we demonstrate that this technology can benefit the wider population with type 2 diabetes requiring insulin and can be safely implemented in the home setting. As a considerable proportion of people with type 2 diabetes struggle to achieve the recommended glycemic targets with currently available therapies, including insulin therapy^14^, fully closed-loop systems offer a new approach to improve glycemic outcomes to reduce the risk of long-term complications.

The proportion of time spent in target glucose range was higher in this study than in the previous study involving people on dialysis (66% versus 57%, respectively)^13^. We postulate that this might be due to the higher personal glucose targets applied for the more vulnerable population requiring dialysis, who have a greater risk of hypoglycemia. The majority of participants in the present study used the default glucose target of 5.8 mmol l^−1^, whereas in the previous study, a personal glucose target of 7.0–7.5 mmol ^−1^ was often applied. The shorter study duration of 20 days in the previous study limits the benefit of the algorithm adaptation to an individual user over time. Analysis of glucose metrics in fortnightly intervals in the present study shows that closed-loop insulin delivery results in an almost immediate improvement in glycemic control compared with standard insulin therapy (proportion of time in target range, 63.1% versus 34.6% during days 1–14, respectively), but the proportion of time in target glucose range improved further to 69% with increased duration of closed-loop therapy (Supplementary Table 4). In this study, very few participants used a glucose sensor as part of their usual care. Therefore, some of the glycemic benefits observed during the closed-loop therapy period may be attributable to use of a continuous glucose monitor alone.

A major contributor to the clinical inertia in the escalation of insulin therapy among healthcare professionals, and a feared side effect of insulin among people with type 2 diabetes, is the risk of hypoglycemia^15,16^. We have shown that fully closed-loop insulin delivery does not increase the risk of hypoglycemia despite improved glycemic control and there were no episodes of severe hypoglycemia during closed-loop therapy. The study cohort spent very little time in hypoglycemia during both intervention periods.

The degree of comorbidity burden in our study population is reflected in the number of nonstudy-related SAEs reported during the study. These were hospital admissions for treatment of comorbidities (diabetic foot disease) or infection. One severe adverse event was related to study procedures and occurred during the closed-loop therapy period (an abscess at the pump cannula site requiring hospital admission for incision and drainage with no long-term sequelae). Closed-loop therapy was continued provided that the participant was able to manage the system themselves and the treating healthcare professionals were satisfied that this was clinically appropriate.

Training and maintenance of study devices by participants was acceptable. One participant withdrew because of difficulty managing the devices, whereas all other participants were able to manage the devices independently and reported a high degree of satisfaction while using the closed-loop system. All participants reported that they were happy to have their glucose levels controlled automatically by the system, and 89% reported that they spent less time managing their diabetes overall. Feedback from users highlighted the elimination of the need for injections or finger-prick testing, and increased confidence in managing blood glucose as key benefits (Supplementary Table 8). There was a trend toward higher levels of hypoglycemia-related anxiety during closed-loop therapy, which may reflect increased awareness and monitoring of glucose levels associated with sensor glucose use. Questionnaire feedback on drawbacks of the closed-loop system mainly consisted of practical annoyances with wearing of devices, connectivity issues between devices and a perceived increase in hypoglycemia episodes. Increased exposure to diabetes technologies in people with type 2 diabetes (insulin pumps and glucose sensors) may mitigate some of these negatives if the glycemic benefits are perceived to be worthwhile. Future-generation closed-loop systems with improved connectivity, longer infusion set wear time and larger insulin reservoirs may also resolve these issues.

The strengths of this study include its randomized crossover design and the inclusion of a wider population with type 2 diabetes self-managing the devices in an outpatient setting, increasing the scope for uptake of this technology. The use of a fully closed-loop system obviates the need for ongoing healthcare professional input with optimization and support following initial training on the devices.

The limitations of the study are that study participants were recruited from a single center and one general practice, and the group was not ethnically diverse, with only one participant not of white ethnicity. Although the present study demonstrated glycemic benefits over the 8-week intervention period, the results should not be generalized beyond this period. The washout period of 2–4 weeks is short for measuring HbA1c. We also did not collect data on the use of boost and ease-off functionality during the closed-loop therapy period. There were a relatively large number of protocol deviations (Supplementary Table 9); however, the majority of these (25 out of 30) were outside of protocol visits with no effect on data analysis.

In conclusion, this study suggests that fully closed-loop insulin delivery is a safe and efficacious approach to manage type 2 diabetes in adults. Larger randomized controlled trials with diverse populations and longer follow-up are required to ensure generalizability across a wider target population and to determine whether it is a cost-effective approach that provides sustained benefits for people with type 2 diabetes requiring insulin therapy.

## Methods

### Study design and participants

The study used an open-label, single-center, randomized, two-period crossover design, contrasting fully closed-loop glucose control using faster-acting insulin aspart (Fiasp, Novo Nordisk) (closed-loop) and standard multiple daily insulin injection therapy (control) during two 8-week periods of unrestricted living. The intervention periods were separated by a 2-week to 4-week washout period during which participants used their pre-study treatment. Assignment of participants to the two groups (with a different order of interventions) was random.

Ethical and regulatory approvals were obtained from the London-Stanmore Ethics Committee and the Medicines and Healthcare Products Regulatory Agency. The study protocol is provided in the Supplementary Information. The safety aspects of the trial were overseen by an independent data and safety monitoring board. The study is registered with ClinicalTrials.gov (NCT04701424).

Recruitment for the study took place through diabetes outpatient clinics at Addenbrooke’s Hospital (Cambridge, UK) and a primary care participant identification center (Granta Medical Practices, Cambridge, UK). Planned population size was 30, to ensure 24 participants completed the study, allowing for a 20% dropout rate.

Inclusion criteria were age 18 years and older with type 2 diabetes requiring subcutaneous insulin therapy and screening HbA1c ≤ 12% (108 mmol mol^−1^). Participants had to be literate in English, willing to perform regular finger-prick blood glucose monitoring and willing to wear study devices and follow study-specific instructions. Exclusion criteria were type 1 diabetes, pregnancy or breastfeeding, severe visual or hearing impairment, allergy to insulin or the adhesive of plasters or serious skin disease affecting device placement, lack of reliable telephone facility for contact, alcohol abuse, Illicit or prescription drug abuse, any physical or psychological disease, or use of medication(s) likely to interfere with the conduct of the trial or interpretation of the results.

Written informed consent was obtained from all study participants before any study-related activities. Participants received £30 for each 8-week study period completed, and all reasonable traveling expenses were reimbursed.

### Randomization and masking

Participants were randomized in a 1:1 ratio to an 8-week period of fully closed-loop glucose control with faster-acting insulin aspart (Fiasp) followed by an 8-week period of standard insulin therapy, or vice versa. Randomization was performed using a web-based, permuted blocks-of-four randomization method to assign study participants to one of the two treatment sequences. Participants and investigators were not masked to the intervention used during each period because of the nature of the interventions.

### Procedures

Study visits and procedures are shown in Supplementary Tables 10 and 11. Participant demographics and medical history, body weight and height, HbA1c and total daily insulin dose were recorded at enrollment. The sex of participants was self-reported.

### Closed-loop insulin delivery system

The closed-loop app (CamAPS HX, CamDiab) involves the Cambridge adaptive model predictive control algorithm (HX software v.0.3.71) on an Android smartphone, which receives data from the continuous glucose monitor (Dexcom G6, Dexcom) and directs insulin delivery via the insulin pump (Dana Diabecare). The closed-loop app is initiated using participants’ total daily insulin dose and body weight. Every 8–12 min, in response to the sensor glucose data, the algorithm calculates an insulin infusion rate and communicates this wirelessly to the insulin pump. Sensor glucose and insulin data are automatically uploaded to the Diasend (https://diasend.com//en) data management platform. Over time, the algorithm adapts to observed glucose patterns, enabling it to tailor insulin delivery more accurately to minimize glucose excursions. The default target glucose used by the closed-loop algorithm is 5.8 mmol l^−1^ and can be adjusted as required between 4.4 mmol l^−1^ and 11.0 mmol l^−1^. Low and high glucose alarms were adjusted according to the participant’s preferences. Participants were trained to use the ‘boost’ and ‘ease-off’ functions within the app as required to increase or decrease insulin, respectively, as directed by the algorithm.

### Closed-loop therapy period

Before the closed-loop therapy period commenced, participants underwent a 1-h to 2-h training session with the study team on the use of the insulin pump, continuous glucose monitoring and closed-loop system. Once participants were deemed competent to use the devices, the closed-loop system was initiated and continued for 8 weeks. The usual insulin therapy of participants was discontinued, but all other medications were continued as directed by their clinical team without interference from the study team. The insulin pump delivered faster-acting insulin aspart continuously as directed by the algorithm, without prandial boluses or carbohydrate announcement.

The study did not interfere with the usual activities or dietary intake of participants. Other diabetes therapies were continued throughout the closed-loop therapy period. Participants were given 24-h access to a study helpline in the event of any study-related issues. At the end of the closed-loop therapy period, devices were removed and the usual insulin therapy of participants was restarted.

### Standard insulin therapy period

During the 8-week control therapy period, a glucose sensor (Dexcom G6) was worn by participants throughout the standard insulin therapy period. Sensor glucose on the sensor glucose receiver was masked to the participant and investigators until the end of the study. Participants continued their usual insulin therapy, and the study team did not make any alterations to participants’ medications. Other diabetes therapies were continued throughout the control therapy period. Fingerstick blood glucose monitoring was performed by participants as per their usual practice. Participants were unrestricted in their usual activities and dietary intake. Participants remained under the care of their local clinical team for glycemic management.

Participants were given 24-h access to a study helpline in the event of any study-related issues. At the end of the standard insulin therapy period, the glucose sensor was removed and the usual insulin therapy of participants was continued.

### Questionnaires

Questionnaires were completed by participants at the end of each study period. These questionnaires include the PAID questionnaire, which is used to assess diabetes distress; the Hypoglycemia Confidence Survey, which is used to analyze patients’ perception of their self-management of hypoglycemia; and the Hypoglycemia Fear Survey-II Worry Scale, which is used to assess hypoglycemia-related anxiety^17–19^. At the end of the closed-loop therapy period, participants completed a closed-loop experience questionnaire to feed back on the closed-loop system, provide suggestions for improvement and indicate whether they would recommend the system to friends or family.

### Endpoints

The primary endpoint was the proportion of time the sensor glucose measurement was in the target glucose range of 3.9–10.0 mmol l^−1^ based on sensor glucose levels during the 8-week study period.

Key endpoints included the proportion of time with sensor glucose above 10.0 mmol l^−1^, mean sensor glucose, HbA1c and the proportion of time with sensor glucose below 3.9 mmol l^−1^. Secondary efficacy endpoints included the proportion of time with sensor glucose below 3.0 mmol l^−1^, the proportion of time with sensor glucose levels in hyperglycemia (>16.7 mmol l^−1^ and >20 mmol l^−1^), variability of glucose measured by s.d. and the coefficient of variation of sensor glucose, and the total daily insulin dose.

Safety endpoints included severe hypoglycemia (capillary glucose < 2.2 mmol l^−1^, or requiring assistance of another person), adverse events, SAEs and device deficiencies.

Utility evaluation included percentage time of sensor glucose availability, and percentage time of closed-loop operation. Psychosocial assessments were measured using questionnaires collected at the end of each study period.

Exploratory endpoints included analysis of a subset of sensor glucose metrics (time in target range, mean glucose, s.d. and time with glucose < 3.9 mmol l^−1^) over daytime (06:00 to 23:59) and nighttime (00:00 to 05:59) periods, and on a fortnightly basis. There was no prespecified analysis disaggregated by sex.

### Sample size

This was an exploratory study with an aim for at least 24 participants to complete both closed-loop and control therapy periods, with a minimum of 48 h of sensor glucose data for each of these participants. As previous studies using closed-loop systems in an inpatient setting and in outpatients requiring dialysis may not provide reliable information about the s.d. of the primary endpoint in this particular population, no formal power calculation was applied. The sample size corresponds to the sample size of previous feasibility closed-loop randomized trials^10,12,13^.

### Statistical analysis

The statistical analysis plan was agreed by the investigators in advance. All analyses were carried out on an intention-to-treat basis. We included data from participants for whom at least 48 h of sensor glucose data were collected in at least one study period. Comparisons were made between values obtained during the 8-week randomized interventions. A two-sample *t*-test on paired differences was used to compare normally distributed variables^20^. A 95% CI was reported for the difference between the interventions. For the primary and all key and secondary sensor glucose metrics, a single value was calculated for each participant for each 8-week period by pooling all sensor glucose readings between the treatment initiation visit and 8 weeks after the initiation visit, or the end of treatment visit, whichever occurred sooner. For highly skewed residuals for key and secondary endpoints, a transformation (windsorization) was used.

A gatekeeping strategy was used, in which the primary endpoint was tested first and, if passing the significance testing, other key endpoints were tested in order. If a nonsignificant result was encountered, formal statistical hypothesis testing was terminated, and analysis of any key endpoints below the one in question (any that were lower in the hierarchy) was considered exploratory.

A per-protocol analysis was carried out for the primary endpoint, restricted to randomized participants, with a minimum of 60% closed-loop use during the closed-loop therapy period and 60% sensor glucose availability during the control therapy period.

Values are reported as mean ± s.d. for normally distributed values or median (IQR) for non-normally distributed values. Endpoints were calculated using GStat software v.2.3 (University of Cambridge, Cambridge, UK), and statistical analyses were performed using SPSS v.2 (IBM Software). All *P* values are two-tailed, and *P* < 0.05 was considered statistically significant.

### Reporting summary

Further information on research design is available in the Nature Portfolio Reporting Summary linked to this article.

## Online content

Any methods, additional references, Nature Portfolio reporting summaries, source data, extended data, supplementary information, acknowledgements, peer review information; details of author contributions and competing interests; and statements of data and code availability are available at 10.1038/s41591-022-02144-z.

## Supplementary information

Supplementary Information Supplementary Tables 1–11. Protocol and statistical analysis plan.

Reporting Summary

## Data Availability

The data that support the findings of this study are available from the corresponding author for the purposes of advancing the management and treatment of diabetes. All data shared will be de-identified. The study protocol and statistical analysis plan are available in the Supplementary Information.
