# Supplementary material for: Fully automated closed-loop insulin delivery in adults with type 2 diabetes: an open-label, single-center, randomized crossover trial
Source: Nat Med. 2023 Jan 11;29(1):203–8. doi: 10.1038/s41591-022-02144-z (PMC9873557; doi:10.1038/s41591-022-02144-z)
Supplement: Supplementary file 1 — Supplementary Tables 1–11. Protocol and statistical analysis plan. [file 41591_2022_2144_MOESM1_ESM.pdf]

nature medicine

Article

https://doi.org/10.1038/s41591-022-02144-z

Fully automated closed-loop insulin delivery in adults with type 2 diabetes: an open-label, single-center, randomized crossover trial

In the format provided by the authors and unedited

Supplementary Information

Table 1. Diabetes treatment regimen at recruitment.

	**Overall (n=26)**
**Insulin regimen, n (%)**	
Basal insulin only	2 (8)
Basal bolus insulin	20 (77)
Pre-mixed insulin	4 (15)
**Oral antihyperglycaemic agents, n (%)**	
Metformin	20 (77)
Sulphonylurea	3 (12)
DPP4 inhibitor	1 (4)
GLP-1 receptor agonist	11 (42)
SGLT2 inhibitor	6 (23)
Thiazolidinedione	1 (4)

DPP4 = dipeptidyl peptidase 4
GLP1 = glucagon-like peptide-1
SGLT2 = sodium-glucose co-transporter-2

Table 2. Per protocol analysis of the primary endpoint.

	Closed loop (n=24)	Control (n=24)	95% CI for treatment difference
Percent time with glucose 3.9 to 10.0 mmol/L	67.8 ± 13.2	33.5 ± 24.4	34.3 (26.9, 41.6)

Data presented are mean ± SD throughout the 8-week study periods
Glucose data are based on sensor glucose measurements

Table 3. Daytime and night-time glucose control and insulin delivery.

	Daytime 0600 to 2359		Night-time 0000 to 0559	
	**Closed-loop (n=26)**	**Control (n=25)**	**Closed-loop (n=26)**	**Control (n=25)**
Percent of time with sensor glucose level (%)				
3.9 to 10.0 mmol/L	64.8 ± 15.3	31.7 ± 24.4	69.3 ± 19.9	33.6 ± 28.3
<3.9 mmol/L	0.6 (0.2, 0.9)	0.0 (0.0, 0.6)	0.3 (0.1, 0.5)	0.1 (0.0, 1.7)
Mean glucose (mmol/L)	9.2 ± 1.4	12.7 ± 3.0	9.1 ± 1.5	12.6 ± 3.4
Glucose SD (mmol/L)	3.0 ± 0.8	3.5 ± 0.8	2.6 ± 0.8	3.2 ± 0.9

Data presented are mean ± SD or median (IQR) throughout the 8-week study periods
Glucose data are based on sensor glucose measurements

Table 4. Glucose endpoints by fortnight.

	Fortnight							
	**1 (days 1-14)**		**2 (days 15-28)**		**3 (days 29- 42)**		**4 (days 43-56)**	
	Closed-loop (n=26)	Control (n=25)	Closed-loop (n=26)	Control (n=25)	Closed-loop (n=25)	Control (n=25)	Closed-loop (n=25)	Control (n=25)
Percent of time with sensor glucose level (%)								
3.9 to 10.0 mmol/L	63.1 ± 18.5	34.6 ± 25.6	66.5 ± 15.3	32.8 ± 26.0	69.8 ± 12.8	31.1 ± 25.4	69.0 + 13.6	30.8 ± 25.7
<3.9 mmol/L	0.31 (0.04, 0.74)	0.14 (0.00, 0.71)	0.50 (0.18, 0.89)	0.00 (0.00, 0.48)	0.38 (0.22, 0.73)	0.00 (0.00, 0.69)	0.40 (0.14, 0.73)	0.00 (0.00, 1.20)
Mean glucose (mmol/L)	9.5 ± 1.7	12.1 ± 2.8	9.2 ± 1.2	12.6 ± 3.1	8.8 ± 1.0	12.8 ± 3.2	8.9 ± 1.1	13.1 ± 3.6
Glucose SD (mmol/L)	3.1 ± 0.8	3.3 ± 0.8	3.0 + 0.9	3.3 ± 0.8	2.7 ± 0.7	3.4 ± 0.9	2.8 ± 0.8	3.4 ± 0.8

Data presented are mean ± SD or median (IQR) throughout the 8-week study periods
Glucose data are based on sensor glucose measurements

Table 5. Primary and key endpoints by treatment sequence

	CL first (n=14)	CL second (n=12)	Control first (n=12)	Control second (n=13)
**Primary endpoint**				
Percent time with glucose 3.9 to 10.0 mmol/L	67.6±14.3	64.8±16.1	35.3±27.0	29.5±23.0
**Key secondary endpoints**				
Percent time with glucose >10.0 mmol/L	32.0±14.3	34.5±15.8	64.4±27.2	69.4±24.2
Mean glucose (mmol/L)	9.1±1.1	9.3±1.4	12.3±2.4	12.9±3.5
HbA1c (mmol/mol)	55±8*	59±9	74±15	71±10
[HbA1c (%)]	[7.2±0.7*]	[7.5±0.9]	[8.9±1.4]	[8.6±0.9]
Percent time with glucose <3.9 mmol/L	0.34 (0.17, 0.67)	0.66 (0.23, 0.90)	0.04 (0.00, 0.52)	0.08 (0.00, 2.16)

Data presented are mean±SD or median (IQR) throughout the 8 week study periods. Glucose data are based on sensor glucose measurements.
One participant randomised to initial use of closed-loop therapy did not cross over to control therapy.
*HbA1c was not measured at the end of the CL period in the one participant who did not complete this arm (n=13).

Table 6. Serious adverse events.

Study period	Description of serious adverse event
Pre-randomisation	Admission to hospital following a fall. Treated for urinary tract infection and COVID-19 infection.
Pre-randomisation	Admission to hospital with acute kidney injury, diarrhoea and hyperkalaemia. Treated for urinary tract infection and peri-anal abscess. Died during admission.
Control	Admission to hospital with infected foot ulcer requiring trans-metatarsal amputation.
Control	Admission to hospital with constipation and abdominal pain. Treated with IV fluids, analgesia and laxatives.
Closed-loop	Admission to hospital with pyelonephritis and treated with IV antibiotics.
Closed-loop	Admission to hospital with infected leg ulcer and treated with IV antibiotics.
Closed-loop	Admission to hospital with abscess at pump cannula insertion site requiring incision and drainage.
Closed-loop	Admission to hospital for left lower limb vascular intervention.

Table 7. Questionnaire scores.

	Closed-loop	Control
	(n=19)	(n=22)
Hypoglycaemia Fear Survey-II Worry Scale	15.0 (6.5, 20.0)	9.5 (6.0, 21.0)
	(n=18)	(n=21)
Hypoglycaemia Confidence Scale	3.3 (3.2, 3.9)	3.4 (2.9, 3.6)
	(n=19)	(n=22)
Problem Areas In Diabetes	22.5 (6.9, 55.6)	20.0 (11.3, 39.7)

Data presented are median (IQR)

Table 8. Responses to closed-loop experience questionnaire (n=19).

	Strongly agree	Agree	Neutral	Disagree	Strongly disagree
Q1. I was happy to have my glucose levels controlled automatically by the system	18 (95)*	1 (5)	0 (0)	0 (0)	0 (0)
Q2. I spent less time to manage my diabetes (glucose testing, adjusting insulin therapy, keeping a diary, data review...)	17 (89)	0 (0)	2 (11)	0(0)	0 (0)
Q3. I was less worried about my glucose control	9 (47)	8 (42)	1 (5)	1 (5)	0 (0)
Q4. I slept better during the nights	9 (47)	5 (26)	3 (16)	2 (11)	0 (0)
Q5. I would recommend closed-loop to others	16 (84)	3 (16)	0 (0)	0 (0)	0 (0)

Q6. What did you like about the Closed-loop system?

- Only having to change cannula every 3 days. Not having to fingerprick and inject 5 times a day. Automatic insulin delivery. Immediate readout of glucose level
- It would make my life so much better and wonderful, and my family would agree
- Knowing I could carry on with my lifestyle without worrying about my blood sugars as I could check them anytime without the fuss of glucose testing and knowing insulin would be dispensed accordingly.
- A lot better control of my glucose levels and reduction in HbA1c
- Not injecting myself all the time
- I was confident to manage much tighter control keeping under 7mmol/L most of the time. It made it possible to take part in strenuous activity without keeping glucose high in fear of hypo. It gave freedom. It gave the ability to dose throughout the day and night. Just brilliant
- The fact it did the thinking for me
- Easy to use and maintain. No finger pricking so fingers not sore.
- Found it immediately beneficial and gradually got used to the highs and lows of my glucose levels and found consistency in my levels. HbA1c improved at the end.
- Takes away the stress of testing/ finger pricks
- Not having to check bloods
- I liked how easy it was to use once I had all the information on its use.
- No need for finger blood testing. Automatic monitoring.
- Better control of insulin. Adjusting my eating habits as could see what raises levels. Peace of mind of sugar levels. Easy to check levels. Not having to remember to take insulin.
- Gives more flexibility at mealtimes.
- A complete life changer.
- Not pricking my finger 8 times a day. No injections.
- Not having to inject at mealtimes
- Looking at the glucose levels as often as I did. Alarms telling me my blood sugar is high or low.

Q7. What are the things you did not like about the system?

- Pump disconnection from app. Bluetooth issues. Short battery life of pump (Dana-i). Pump clip that secures insulin in place sometimes not secured
- Refilling the insulin pump and having to make sure I had all the equipment to do so if I was away from home
- The batteries on the pump not lasting and on 2 occasions dropping to low glucose levels
- Being attached to the pump all the time. Having to be careful not to pull the cannula out. Refilling with insulin every 3-4 days
- Need better ability to hold it on body
- Sometimes needle tubing got caught on kitchen drawers and pulled out. Sticky pads small and not sticky enough.
- Dropping out of transmitter to app. Alarm after 3 days to refill pump even if still sufficient insulin in pump
- The pump and phone lost connection often and figuring out how to correct pump errors.
- I found it too easy to pull the patches off. I experienced hypos that I don't normally experience
- Risk of hypos at night if communication to pump fails. Pump rate does not appear to change.
- Had to set alarms to self-check glucose at night. Most users would not understand the system's foibles
- A bit fiddly
- I didn't feel in control of my insulin on a long-term basis. I couldn't wear the bag (pouch) as ut was too rough.
- Waking up feeling low in the early morning or being woken up by the system telling me I'm low. Connectivity problems between sensor and phone.
- I thought that every 3 days was a little too often to change the insulin

Q8. Would you like the closed-loop system to have additional features? If yes, which ones?

- A good clip with the pump. Not having to refill insulin so often. A smaller device than a mobile- something easier to carry all the time
- The ability to fill a separate pump and take one with you if you leave home
- Easier input for entering refill amount of insulin on pump. Buttons on pump slow and if pump talked to phone, it reset the amount added. Ability to press and hold so numbers click up in 5-10 units would be beneficial.
- Less alarms
- A better design belt to wear it
- For it to be readily available
- The ability to turn off insulin for a limited time.

*Values are n (%)

Table 9. Protocol deviations.

	**Overall***
Number of protocol deviations	30
Number (%) of participants with protocol deviations	20 (77)
Number (%) of participants with protocol deviations	
1	11 (42)
2	8 (31)
3	1 (4)
Type of protocol deviation	
Out of protocol visit for resetting back-up data collection	14
Out of protocol visit for hardware or supplies	10
Insulin injection during closed-loop period	2
HbA1c sample (late collection, haemolysed requiring repeat sample)	2
GLP-1 receptor agonist stopped during closed-loop period	1
Additional participant training on devices	1

Values are n (%)
*Control and closed-loop periods combined

Table 10. Schedule of study visits / phone contacts when closed-loop intervention precedes control period.

	Visit/ contact	Description	Start relative to previous / next Visit / Activity	Duration
	Visit 1	Recruitment visit: Consent, baseline assessments including questionnaires Hba1c measurement	-	1-2 hours
**RANDOMISATION**				
**CL Intervention (8 weeks)**	Visit 2	CGM, Insulin pump and closed loop training Competency assessment and initiation of closed loop	Within 1 to 3 weeks of Visit 1 Training visits can be repeated if competency not achieved	3-4 hours
	Contact 1*	Review use of study devices	24h after Visit 2 (±3 days)	<0.5 hour
	Contact 2*	Review use of study devices	7 days after Visit 2 (±3 days)	<0.5 hour
	Contact 3*	Review use of study devices	4 weeks after Visit 2 (3 days)	<0.5 hour
	Visit 3	End of closed-loop treatment period (8 weeks) Return devices. Revert back to usual diabetes therapy. Questionnaires HbA1c measurement	After 8 weeks of Visit 2	1 hour
		**Washout period**	Immediately after Visit 3	2-4 weeks
**Control period (8 weeks)**	Visit 4	Blinded CGM insertion Review of diabetes management	Within 2-4 weeks of Visit 3	1-2 hours
	Contact 4*	Review diabetes management	24h after Visit 4 (±3 days)	<0.5 hour
	Contact 5*	Review diabetes management	7 days after Visit 4 (±3 days)	<0.5 hour
	Contact 6*	Review diabetes management.	4 weeks after Visit 4 (±3 days)	<0.5 hour
	Visit 5	End of control period (8 weeks) Questionnaires HbA1c measurement	After 8 weeks of Visit 4	1 hour
* could be done at home or phone/email				

Table 11. Schedule of study visits / phone contacts when control period precedes closed-loop intervention.

	Visit/ contact	Description	Start relative to previous / next Visit / Activity	Duration
	Visit 1	Recruitment visit: Consent, baseline assessments including questionnaires HbA1c measurement	-	1-2 hours
RANDOMISATION				
Control period (8 weeks)	Visit 2	Blinded CGM insertion Review of diabetes management	Within 1 to 3 weeks of Visit 1	1-2 hours
	Contact 1*	Review diabetes management	24h after Visit 2 (±3 days)	<0.5 hour
	Contact 2*	Review diabetes management	7 days after Visit 2 (±3 days)	<0.5 hour
	Contact 3*	Review diabetes management.	4 weeks after Visit 2 (±3 days)	<0.5 hour
	Visit 3	End of control period (8 weeks Questionnaires HbA1c measurement	After 8 weeks of Visit 2	1-2 hours
		**Washout period**	Immediately after Visit 3	2-4 weeks
CL Intervention (8 weeks)	Visit 4	CGM, Insulin pump and closed loop training Competency assessment and initiation of closed loop	Within 2 to 4 weeks of Visit 3 Training visits can be repeated if competency not achieved	3-4 hours
	Contact 4*	Review use of study devices	24h after Visit 4 (±3 days)	<0.5 hour
	Contact 5*	Review use of study devices	7 days after Visit 4 (±3 days)	<0.5 hour
	Contact 6*	Review use of study devices	4 weeks after Visit 4 (±3 days)	<0.5 hour
	Visit 5	End of closed-loop treatment period (8 weeks) Return devices. Revert back to usual diabetes therapy. Questionnaires HbA1c measurement	After 8 weeks of Visit 4	1-2 hours
* could be done at home or phone/email				

INSELSPITAL
Universitätsklinik
für Diabetologie, Endokrinologie,
Ernährungsmedizin und Metabolismus

UNIVERSITY OF CAMBRIDGE

NHS
Cambridge
University Hospitals
NHS Foundation Trust

Clinical Study Protocol

Study Title: An open-label, two-centre (phase 1), single-centre (phase 2), randomised, 2-period cross-over study to assess the efficacy, safety and utility of fully closed-loop insulin delivery in comparison with standard care, in adults with type 2 diabetes requiring maintenance dialysis (phase 1) and in adults with type 2 diabetes not requiring dialysis (phase 2)

Short Title: Closed-loop in adults with T2D (AP-Renal)

Protocol Version: 5.0 04 February 2022

**Chief Investigator**	Prof Roman Hovorka<br>University of Cambridge Metabolic Research Laboratories<br>Level 4, Wellcome Trust-MRC Institute of Metabolic Science<br>Box 289, Addenbrooke's Hospital, Hills Rd<br>Cambridge CB2 0QQ<br>UK Tel: +44 (0)1223 762 862<br>Fax: +44 (0)1223 330 598<br>E-mail: rh347@cam.ac.uk

This protocol has been written in accordance with current ISO 14155:2011 standard

**Protocol Design**	Prof Roman Hovorka<br>University of Cambridge Metabolic Research Laboratories<br>Level 4, Wellcome Trust-MRC Institute of Metabolic Science<br>Box 289, Addenbrooke's Hospital, Hills Rd<br>Cambridge CB2 0QQ<br>UK Tel: +44 (0)1223 762 862<br>Fax: +44 (0)1223 330 598<br>E-mail: rh347@cam.ac.uk<br><br>Dr Lia Bally<br>Department of Diabetes, Endocrinology, Clinical Nutrition and Metabolism<br>Inselspital, Bern University Hospital<br>University of Bern<br>Bern<br>Switzerland<br>Phone: +41 (0)31 632 36 77<br>E-mail: lia.bally@insel.ch
**Principal Clinical Investigators**	Dr Andrew Fry<br>Department of Renal Medicine<br>Box 118<br>Addenbrooke's Hospital<br>Hills Road<br>Cambridge CB2 0QQ<br>UK<br>Phone: +44 1223 216017<br>E-mail: andrew.fry@addenbrookes.nhs.uk<br><br>Dr Lia Bally<br>Department of Diabetes, Endocrinology, Clinical Nutrition and Metabolism<br>Inselspital, Bern University Hospital<br>University of Bern<br>Bern<br>Switzerland<br>Phone: +41 (0)31 632 36 77<br>E-mail: lia.bally@insel.ch<br><br>Dr Mark Evans<br>Wolfson Diabetes Endocrine Clinic,<br>Cambridge University Hospitals NHS Trust<br>IMS Metabolic Research Laboratories<br>Box 289 Addenbrookes Biomedical Campus<br>Hills Road<br>Cambridge CB2 0QQ<br>Phone: +44 (0)1223 336994<br>E-mail: mle24@cam.ac.uk
**Clinical Investigators**	Dr Charlotte Boughton<br>Institute of Metabolic Science<br>Box 289, Level 4<br>Addenbrooke's Hospital

	Hills Road<br>Cambridge<br>CB2 0QQ<br>UK<br>Phone: +44 1223 769066<br>E-mail: cb2000@medschl.cam.ac.uk<br><br>Mrs Sara Hartnell<br>Institute of Metabolic Science<br>Box 289, Level 4<br>Addenbrookes Hospital<br>Hills Road<br>Cambridge<br>CB2 0QQ<br>UK<br>Phone: +44 1223 596451<br>sara.hartnell@addenbrookes.nhs.uk<br><br>Dr Aideen Daly<br>Institute of Metabolic Science<br>Box 289, Level 4<br>Addenbrookes Hospital<br>Hills Road<br>Cambridge<br>CB2 OQQ<br>UK<br>Phone: +44 1223 769080<br>ad2075@medschl.cam.ac.uk
**Other Investigators**	Dr Malgorzata E Wilinska<br>Institute of Metabolic Science<br>University of Cambridge<br>Box 289, Level 4<br>Addenbrooke's Hospital<br>Hills Road, Cambridge<br>CB2 0QQ<br>UK<br>Phone: +44 1223 769 065<br>Fax: +44 1223 336 996<br>E-mail: mew37@cam.ac.uk
**Study Co-ordinator**	Mrs Alina Cezar<br>Institute of Metabolic Science<br>University of Cambridge<br>Box 289, Level 4<br>Addenbrooke's Hospital<br>Hills Road, Cambridge<br>CB2 0QQ<br>UK<br>Phone: + 44(0)1223 769068<br>E-mail: ac2367@medschl.cam.ac.uk

<table>
<tr><td>Study Statistician</td><td colspan="2">Prof Roman Hovorka
Institute of Metabolic Science
University of Cambridge
Box 289, Level 4
Addenbrooke's Hospital
Hills Road, Cambridge
CB2 0QQ
UK
Phone: +44 1223 762 862
Fax: +44 1223 336 996
E-mail: rh347@cam.ac.uk</td></tr>
<tr><td rowspan="2">Study Sponsor</td><td colspan="2">Cambridge University Hospitals NHS Foundation Trust, jointly with University of Cambridge</td></tr>
<tr><td>Tamsin Sayer, Assistant Director School of Clinical Medicine
Research Operations Office
University of Cambridge
Greenwich House
Madingley Road
Cambridge CB3 0TX
Phone: +44 (0) 1223 333543
Email:
ClinicalSchoolContractsAdministration@admin.cam.ac.uk</td><td>Stephen Kelleher
Cambridge University Hospitals NHS Foundation Trust
Box 277, Addenbrooke's Hospital
Hills Road, Cambridge, CB2 0QQ, UK
Phone: +44 (0) 1223 217418
Fax: +44 (0) 1223 348494
E-mail:
r&denquiries@addenbrookes.nhs.uk</td></tr>
<tr><td rowspan="2">Representative of the Sponsor and Principal Investigator (Switzerland)</td><td colspan="2">Bern University Hospital, University of Bern</td></tr>
<tr><td colspan="2">Dr Lia Bally
Department of Diabetes, Endocrinology, Clinical Nutrition and Metabolism
Inselspital, Bern University Hospital
University of Bern
Bern
Switzerland
Phone: +41 (0)31 632 36 77
E-mail: lia.bally@insel.ch</td></tr>
<tr><td>Funders</td><td colspan="2">UK: NIHR Cambridge Biomedical Research Council and Novo Nordisk UK Foundation

Switzerland: Swiss Society of Endocrinology and Diabetes, Swiss Kidney Foundation</td></tr>
</table>

PROTOCOL SIGNATURE PAGE

The signature below documents the approval of the protocol entitled **“An open-label, two-centre (phase 1), single-centre (phase 2), randomised, 2-period cross-over study to assess the efficacy, safety and utility of fully closed-loop insulin delivery in comparison with standard care, in adults with type 2 diabetes requiring maintenance dialysis (phase 1) and in adults with type 2 diabetes not requiring dialysis (phase 2).** Version 5.0 dated 04/02/2022 and provides the necessary assurances that this study will be conducted according to all stipulations of the protocol, the principles of GCP and the appropriate reporting requirements.

Signature ……………………………………. Date……………………….

Prof Roman Hovorka, Chief Investigator

SITE SIGNATURE PAGE

I have read the attached protocol entitled " **An open-label, two-centre (phase 1), single-centre (phase 2), randomised, 2-period cross-over study to assess the efficacy, safety and utility of fully closed-loop insulin delivery in comparison with standard care, in adults with type 2 diabetes requiring maintenance dialysis (phase 1) and in adults with type 2 diabetes not requiring dialysis (phase 2).** Version 5.0 dated 04/02/2022, and agree to abide by all provisions set forth therein.

I agree to comply with the conditions and principles of Good Clinical Practice as outlined in the European Clinical Trials Directives 2001/20/EC and the GCP Directive 2005/28/EC.

I agree to ensure that the confidential information contained in this document will not be used for any other purpose other than the evaluation or conduct of the clinical investigation without the prior written consent of the Sponsor.

Signature ……………………………………. Date………………………

Name ……………………………………

Site, Country………………………….

Principal Clinical Investigator

Table of Contents

LIST OF ABBREVIATIONS AND RELEVANT DEFINITIONS ... 10

1 STUDY SYNOPSIS ... 12

2 SUMMARY ... 17

3 BACKGROUND ... 18

3.1 INTRODUCTION ... 18
3.2 MANAGEMENT OF TYPE 2 DIABETES IN PATIENTS NOT REQUIRING DIALYSIS ... 19
3.3 MANAGEMENT OF TYPE 2 DIABETES IN PATIENTS REQUIRING DIALYSIS ... 19
3.4 GLYCAEMIC CONTROL IN PEOPLE WITH TYPE 2 DIABETES REQUIRING DIALYSIS ... 20
3.5 HYPOGLYCAEMIA IN PEOPLE WITH TYPE 2 DIABETES REQUIRING DIALYSIS ... 21
3.6 GLYCAEMIC TARGETS IN PEOPLE WITH DIABETES REQUIRING DIALYSIS ... 22
3.7 GLYCAEMIC TARGETS IN PEOPLE WITH DIABETES NOT REQUIRING DIALYSIS ... 23
3.8 CLOSED-LOOP INSULIN DELIVERY ... 24
3.9 CLOSED-LOOP RESEARCH IN CAMBRIDGE ... 24
3.9.2 Studies of closed-loop in children and adolescents with type 1 diabetes in the clinical research facility ... 25
3.9.3 Studies of closed-loop in adults with type 1 diabetes in the clinical research facility ... 25
3.9.4 Overnight closed-loop study in children and adolescents with type 1 diabetes in home setting ... 26
3.9.5 Overnight closed-loop studies in adults with type 1 diabetes in home setting ... 26
3.10 RISK AND BENEFITS ... 29
3.11 CAMAPS HX FULLY-AUTOMATED CLOSED LOOP SYSTEM TO BE USED IN THE PRESENT STUDY ... 29
3.12 RATIONALE FOR THE PRESENT STUDY ... 31

4 OBJECTIVES ... 32

4.1 EFFICACY ... 32
4.2 SAFETY ... 32
4.3 UTILITY ... 32

5 STUDY DESIGN ... 32

6 STUDY PARTICIPANTS ... 34

6.1 STUDY POPULATION ... 34
6.1.1 Inclusion criteria ... 34
6.1.2 Exclusion criteria ... 34
6.2 RECRUITMENT AND INFORMED CONSENT ... 35

7 METHODS UNDER INVESTIGATION ... 35

7.1 NAME AND DESCRIPTION OF THE METHOD OF INVESTIGATION ... 35
7.2 INTENDED PURPOSE ... 35
7.3 METHOD OF ADMINISTRATION ... 35
7.4 REQUIRED TRAINING ... 36
7.5 PRECAUTIONS ... 36
7.6 ACCOUNTABILITY OF THE METHOD UNDER INVESTIGATION ... 36

8 STUDY SCHEDULE ... 36

8.1 OVERVIEW ... 36
BASELINE VISIT (VISIT 1) ... 42
8.2 RANDOMISATION ... 43
8.3 POST-RANDOMISATION TRAINING (VISITS 2 AND 4) ... 43

8.3.1 CLOSED LOOP INTERVENTION 43
8.3.2 STANDARD THERAPY (CONTROL INTERVENTION) 44
8.4 CONTACTS AFTER INITIATION OF STUDY ARM 44
8.5 END OF FIRST STUDY ARM VISIT (VISIT 3) 44
8.6 WASHOUT 44
8.7 END OF STUDY VISIT (VISIT 5) 45
8.8 PARTICIPANT WITHDRAWAL CRITERIA 45
8.9 STUDY STOPPING CRITERIA 46
8.10 SUPPORT TELEPHONE LINE 46
8.11 SUBJECT REIMBURSEMENT 46

9 ENDPOINTS 46

9.1 EFFICACY ENDPOINTS 46
9.1.1 PRIMARY EFFICACY ENDPOINT 46
9.1.2 OTHER KEY ENDPOINTS 46
9.1.3 SECONDARY EFFICACY ENDPOINTS 46
9.1.4 Exploratory endpoints 47
9.2 SAFETY EVALUATION 47
9.3 UTILITY EVALUATION 47

10 ASSESSMENT AND REPORTING OF ADVERSE EVENTS 47

10.1 DEFINITIONS 47
10.1.1 REPORTABLE ADVERSE EVENTS 47
10.1.2 ADVERSE EVENTS 48
10.1.3 ADVERSE DEVICE EFFECT 48
10.1.4 SERIOUS ADVERSE EVENT 48
10.1.5 SERIOUS ADVERSE DEVICE EFFECT 49
10.1.6 UNANTICIPATED SERIOUS ADVERSE DEVICE EFFECT 49
10.1.7 DEVICE DEFICIENCIES 49
10.1.8 ADVERSE EVENT INTENSITY 50
10.1.9 ADVERSE EVENT CAUSALITY 50
10.2 RECORDING AND REPORTING OF ADVERSE EVENTS, SERIOUS ADVERSE EVENTS AND DEVICE DEFICIENCIES 51
10.2.1 MONITORING PERIOD OF ADVERSE EVENTS 51
10.2.2 RECORDING AND REPORTING OF ADVERSE EVENTS 51
10.2.3 SEVERE HYPOGLYCAEMIA 52
10.2.4 REPORTING OF SERIOUS ADVERSE EVENTS AND SERIOUS ADVERSE DEVICE EFFECTS 52
10.2.5 RECORDING AND REPORTING OF DEVICE DEFICIENCIES 55
10.2.6 Healthcare arrangements and compensation for adverse events 55
10.3 ANTICIPATED ADVERSE EVENTS, RISKS AND BENEFITS 56
10.3.1 RISKS AND ANTICIPATED ADVERSE EVENTS 56
10.3.2 HYPOGLYCAEMIA AND HYPERGLYCAEMIA 56
10.3.3 FINGER-STICK BLOOD GLUCOSE MEASUREMENTS 57
10.3.4 INSULIN INJECTION THERAPY 57
10.3.5 INSULIN PUMP THERAPY 57
10.3.6 CONTINUOUS GLUCOSE MONITORING 57
10.3.7 Dialysis related events (applicable to phase 1 only) 58
10.3.8 QUESTIONNAIRES 58
10.3.9 RISK ANALYSIS AND RESIDUAL RISK ASSOCIATED WITH THE INVESTIGATIONAL DEVICE 58
10.4 BENEFITS 59
10.5 DATA SAFETY MONITORING BOARD (DSMB) 59

11 METHODS AND ASSESSMENTS 60

11.1 PROCEDURES 60

11.1.1 WEIGHT 60

11.1.2 Blood Glucose Meter Data 60
11.2 ASSESSMENT FOR SAFETY 60
11.3 ASSESSMENT FOR EFFICACY 60
11.4 QUESTIONNAIRES 61
12 STUDY MATERIALS AND PRODUCTS 61
12.1 INSULIN 61
12.2 MULTIPLE DAILY INSULIN INJECTIONS DURING CONTROL INTERVENTION AND WASH OUT PERIOD 61
12.3 INSULIN PUMP 61
12.4 CONTINUOUS SUBCUTANEOUS GLUCOSE MONITOR 62
12.5 BLOOD GLUCOSE METER 62
12.6 COMPUTER-BASED ALGORITHM 62
13 DATA ANALYSIS 62
13.1 PRIMARY ENDPOINT ANALYSIS 62
13.2 OTHER KEY ENDPOINTS 63
13.3 SECONDARY ENDPOINTS 64
13.4 SAFETY EVALUATION 65
13.5 UTILITY EVALUATION 65
13.6 QUESTIONNAIRES 65
13.7 EVALUATIVE PERIODS 66
13.8 INTERIM MONITORING AND ANALYSES 66
13.9 SAMPLE SIZE AND POWER CALCULATIONS 66
14 CASE REPORT FORMS 66
15 DATA MANAGEMENT 67
15.1 FURTHER INFORMATION ON DATA MANAGEMENT – SWITZERLAND ONLY (APPLIES TO PHASE 1 ONLY) 67
15.1.1 Hardware and software 68
15.1.2 Data security, access and backup 68
15.1.3 Analysis and archiving 68
16 ETHICS 69
16.1 RESEARCH ETHICS COMMITTEE AND INSTITUTIONAL REVIEW BOARD 69
16.2 INFORMED CONSENT OF STUDY SUBJECTS 69
17 AMENDMENTS TO THE PROTOCOL 70
18 DEVIATIONS FROM THE PROTOCOL 70
19 STUDY MANAGEMENT 70
19.1 DATA AND SAFETY MONITORING BOARD (DSMB) 70
19.2 TRIAL STEERING COMMITTEE (TSC) 71
20 RESPONSIBILITIES 71
20.1 CHIEF INVESTIGATOR 71
20.2 PRINCIPAL CLINICAL INVESTIGATOR 71
21 REPORTS AND PUBLICATIONS 72
22 TIMETABLE 72
23 RETENTION OF STUDY DOCUMENTATION 72
24 INDEMNITY STATEMENTS 72
25 REFERENCES 74
26 DOCUMENT AMENDMENT HISTORY 80

List of abbreviations and relevant definitions

ACCORD	Action to Control Cardiovascular Risk in Diabetes
ADA	American Diabetes Association
ADE	Adverse Device Effect
ADVANCE	Action in Diabetes and Vascular Disease: Preterax and Diamicron MR Controlled Evaluation
AE	Adverse Event
AP	Artificial Pancreas
AR	Adverse Reaction
ASADE	Anticipated Serious Adverse Device Effect
AUC	Area Under the Curve
BMI	Body Mass Index
CCG	Clinical Commissioning Group
CCTU	Cambridge Clinical Trials Unit
CE	Conformité Européenne (CE-mark)
CGM	Continuous Glucose Monitoring
CI	Chief Investigator *or* Confidence Interval
CL	Closed Loop
CRF	Case Report Form
CSII	Continuous Subcutaneous Insulin Infusion
DCCT	Diabetes Control and Complications Trial
DKA	Diabetic Ketoacidosis
DSMB	Data Safety and Monitoring Board
eCRF	Electronic Case Report Form
EudraCT	European Clinical Trial Database
FDA	US Food and Drug Administration
GCP	Good Clinical Practice
HbA1c	Glycated haemoglobin A1c
HD	Haemodialysis

HFS	Hypoglycaemia Fear Survey
IDE	US Investigational Device Exemption
IRB	Institutional Review Board
i.v.	Intravenous
MDI	Multiple Daily Injection therapy
MHRA	Medicine and Healthcare products Regulatory Agency
MPC	Model-Predictive-Control
NHS	National Health Service
NICE	National Institute for Health and Care Excellence
PD	Peritoneal Dialysis
PI	Principal Investigator
QALY	Quality-Adjusted Life Years
R & D	Research and Development
RCT	Randomised Controlled Trial
REC	Research Ethics Committee
RRT	Renal Replacement Therapy
s.c.	Subcutaneous
SADE	Serious Adverse Device Effect
SAE	Serious Adverse Event
SAP	Sensor Augmented Pump Therapy
SD	Standard Deviation
T1D	Type 1 Diabetes Mellitus
T2D	Type 2 Diabetes Mellitus
TSC	Trial Steering Committee
UKPDS	UK Prospective Diabetes Study
USADE	Unanticipated Serious Adverse Device Effect
VADT	Veterans Affairs Diabetes
WHO	World Health Organisation

1 Study synopsis

**Title of clinical trial**	An open-label, two-centre (phase 1), single-centre (phase 2), randomised, 2-period cross-over study to assess the efficacy, safety and utility of fully closed-loop insulin delivery in comparison with standard care, in adults with type 2 diabetes requiring maintenance dialysis (phase 1) and in adults with type 2 diabetes not requiring dialysis (phase 2)
**Short title**	Closed-loop in adults with T2D (AP-Renal)
**Sponsors name**	Cambridge University Hospitals NHS Foundation Trust and University of Cambridge, Cambridge, UK
**Medical condition or disease under investigation**	Type 2 diabetes
**Purpose of clinical trial**	To determine the efficacy, safety and utility of fully automated closed-loop insulin delivery in the home setting in adults with type 2 diabetes requiring maintenance dialysis. In phase 2, the same will be carried out in adults with type 2 diabetes who do not require maintenance dialysis.
**Study objectives**	The study objective is to compare fully automated closed-loop insulin delivery with usual care in adults with type 2 diabetes requiring maintenance dialysis (phase 1) and in those not requiring dialysis (phase 2).<br>1. EFFICACY: The objective is to assess the ability of fully-automated closed-loop insulin delivery in maintaining CGM glucose levels within the target range from 5.6 to 10.0 mmol/l as compared to usual care in adults with type 2 diabetes requiring maintenance dialysis (phase 1) and in those not requiring dialysis (phase 2).<br>2. SAFETY: The objective is to evaluate the safety of fully automated closed-loop insulin delivery in terms of episodes and severity of hypoglycaemia, and nature and severity of other adverse events.<br>3. UTILITY: The objective is to determine the acceptability and duration of use of the closed-loop system.

**Study design**	An open-label, two-centre (phase 1) and single centre (phase 2) randomised, two-period crossover study comparing fully automated closed-loop insulin delivery with usual care in adults with type 2 diabetes requiring dialysis for phase 1, and not requiring dialysis for phase 2.<br><br>Phase 1 will involve two intervention periods in the home setting lasting 20 days each with a 2-4 week washout period. Phase 2 will consist of two intervention periods lasting 8 weeks each with a 2-4 week washout period. The order of the two interventions in each phase will be random.
**Study endpoints**	The primary endpoint is the time spent in the target glucose range (5.6 to 10.0 mmol/l for phase 1 and 3.9 to 10.0 mmol/l for phase 2) based on CGM glucose levels during the home stay.<br><br>Other key endpoints:<br>• Time spent with sensor glucose above target (10.0 mmol/l)<br>• Average of sensor glucose levels<br>• HbA1c (phase 2 only)<br>• Time spent with sensor glucose <3.9 mmol/l<br><br>Secondary endpoints include:<br>• Time spent with sensor glucose below target (5.6 mmol/l) (phase 1 only)<br>• Time spent with sensor glucose <3.0 mmol/l)<br>• Time spent with sensor glucose levels > 16.7 mmol/l)<br>• Time spent with sensor glucose levels in significant hyperglycaemia (glucose levels > 20 mmol/l)<br>• Standard deviation and coefficient of variation of sensor glucose levels<br>• AUC of glucose below 3.5 mmol/l (63 mg/dl) (phase 1 only)<br>• Total daily insulin requirements<br>• Average inter-dialytic weight gain (phase 1 only)
**Safety evaluation**	Assessment of frequency and severity of hypoglycaemic episodes and nature and severity of other adverse events.

**Utility evaluation**	Assessment of the acceptability and duration of use of the closed-loop system.
**Participating clinical centres**	UK<br>1. Addenbrooke's Hospital, Cambridge University Hospitals NHS Foundation Trust, Cambridge,<br>Switzerland<br>1. Bern University Hospital, Bern (phase 1 only)
**Sample size**	32 adults completing phase 1, 24 adults completing phase 2. Up to 40 (phase 1) and 30 (phase 2) subjects will be recruited to allow for dropouts.
**Summary of eligibility criteria**	Key inclusion criteria:<br>1. Age 18 years or over<br>2. Diagnosis of type 2 diabetes using standard diagnostic practice<br>3. Requirement for maintenance dialysis (phase 1 only)<br>4. Current treatment with subcutaneous insulin<br>5. Screening HbA1c ≤ 12% (108mmol/mol) on analysis from local laboratory<br>6. Subject is willing to perform regular finger-prick blood glucose monitoring<br>7. Willingness to wear study devices<br>8. Literate in English (UK) (phase 1 and 2) or German (Switzerland) (phase 1 only)<br><br>Key exclusion criteria:<br>1. Physical or psychological condition likely to interfere with the normal conduct of the study and interpretation of the study results as judged by the investigator<br>2. Known or suspected allergy to insulin<br>3. Lack of reliable telephone facility for contact<br>4. Pregnancy, planned pregnancy, or breast feeding<br>5. Severe visual impairment<br>6. Severe hearing impairment<br>7. Medically documented allergy towards the adhesive (glue) of plasters

	8. Serious skin diseases located at places of the body, which potentially are possible to be used for localisation of the glucose sensor<br>9. Illicit drugs abuse<br>10. Prescription drugs abuse<br>11. Alcohol abuse
**Maximum duration of study for a participant**	12 weeks (3 months) for phase 1 and 20 weeks (5 months) for phase 2
**Recruitment**	Participants will be recruited through the adult diabetes outpatient clinics and GP practices within the West Suffolk, Cambridge and Peterborough Clinical Commissioning Groups (CCG) (phase 1 and 2) or haemodialysis units at participating centres (phase 1).
**Consent**	Written informed consent will be obtained from participants according to Research Ethics Committee (REC) requirements.
**Screening and baseline assessment**	Eligible participants will undergo a baseline evaluation including medical (diabetes) history and current therapy. A baseline HbA1c will be taken.
**Randomisation**	Eligible participants will be randomised in a 1:1 ratio using randomisation software to the use of fully automated closed-loop insulin delivery or to usual care for the study intervention period with a 2-4 week washout period between the two interventions.
**1. Closed loop arm**	Following randomisation, participants in the closed-loop group will receive training to cover key aspects of insulin pump use and CGM. Competency on the use of study devices will be evaluated<br><br>Once competent in the use of the study pump and CGM, participants will receive training required for safe and effective use of the closed-loop system. During a 2-4 hour session participants will operate the system under the supervision of the clinical team. Competency on the use of closed-loop system will be evaluated. Thereafter, participants are expected to use closed-loop for 20 days (phase 1) or 8 weeks (phase 2) without supervision or remote monitoring.<br><br>• All participants will be provided with 24 hour telephone helpline and will also be given written instructions about when to contact clinical team.

**2. Standard therapy (control arm)**	Participants in the control group will continue with standard insulin therapy with blinded CGM for 20 days (phase 1) or 8 weeks (phase 2).
**Study contacts**	In phase 1, follow up contacts will be made within 24 hours of starting each treatment arm and then at weekly intervals thereafter. In phase 2, contacts will be made at 24 hours, 1 week, then monthly until the end of the intervention period.
**End of study assessments**	Validated questionnaires evaluating the impact of the technology on diabetes management and quality of life will be completed. A blood sample for HbA1c will be taken at the end of each study arm (phase 2). Participants will resume usual care.
**Procedures for safety monitoring during trial**	Standard operating procedures for monitoring and reporting of all adverse events (AE) will be in place, including serious adverse events (SAE), serious adverse device effects (SADE).<br><br>A data safety and monitoring board (DSMB) will be informed of all serious adverse events and any unanticipated serious adverse device effects that occur during the study and will review compiled adverse event data at periodic intervals.
**Criteria for withdrawal of patients on safety grounds**	A participant may terminate participation in the study at any time without necessarily giving a reason and without any personal disadvantage. An investigator can stop the participation of a subject after consideration of the benefit/risk ratio. Possible reasons are:<br>1. Serious adverse events<br>2. Significant protocol violation or non-compliance<br>3. Failure to satisfy competency assessment<br>4. Decision by the investigator, or the Sponsor, that termination is in the participant’s best medical interest<br>5. Pregnancy, planned pregnancy, or breast feeding<br>6. Allergic reaction to insulin<br><br>Efforts will be made to retain participants in follow up for the final primary outcome assessment even if the intervention is discontinued, unless the investigator believes that it will be harmful for the participant to continue in the trial.

2 Summary

The main objective of this study is to determine the efficacy, safety and utility of fully automated closed-loop glucose control in the home setting in adults with type 2 diabetes (T2D). Phase 1 will focus on adults with T2D requiring maintenance dialysis as this is a subgroup presenting unique challenges in diabetes management, glucose regulation and insulin titration. Phase 2 will take a more generalisable approach and will assess the efficacy of closed-loop insulin delivery in adults with T2D who are not on maintenance dialysis. This study builds on previous and on-going studies of closed-loop systems that have been performed in Cambridge in adults with type 1 diabetes (T1D) in the home setting, and in adults with T2D in the inpatient setting.

This is an open-label, two-centre (phase 1) and single centre (phase 2), randomised, cross-over study, involving two home study periods during which glucose levels will be controlled either by a fully automated closed-loop system or by participants' usual insulin therapy in random order. Treatment arms will be 20 days in phase 1, and 8 weeks in phase 2 with a 2-4 week washout period between treatments. For phase 1, a total of up to 40 adults with T2D requiring maintenance dialysis will be recruited through outpatient clinics or the dialysis unit, to allow for 32 completed participants available for assessment. For phase 2, up to 30 adults with T2D, not on dialysis, will be recruited via outpatient clinics and local GP practices within the West Suffolk, Cambridge and Peterborough Clinical Commissioning Groups to allow for 24 completed participants at the end of the study.

Participants will receive appropriate training by the research team on the safe use of the study devices (insulin pump and continuous glucose monitoring (CGM) and closed-loop insulin delivery system). Participants in the control arm will continue with standard therapy and will wear a blinded CGM system.

The primary outcome is time spent with glucose levels in the target range between 5.6 and 10.0 mmol/L for phase 1, and between 3.9 and 10.0 mmol/L for phase 2 as recorded by CGM. Secondary outcomes are the time spent with glucose levels above and below target, as recorded by CGM, change in Hba1c, and other CGM-based metrics in addition to insulin requirements. Safety evaluation comprises the tabulation of severe hypoglycaemic episodes.

3 Background

3.1 Introduction

Type 2 diabetes (T2D) is a condition characterised by chronic hyperglycaemia due to defects in insulin secretion, action, or both (1). The burden of T2D is widespread with an estimated 415 million people affected worldwide, which is expected to double within the next 20 years (2). Although more commonly seen in the older population, incidence of T2D is also increasing in younger age groups, presumably due to physical inactivity, calorie dense diet and rising obesity levels (3).

It is estimated that annual global health expenditure on diabetes is 760 million USD and 50% of this is spent on treating complications of the disease (4). The fear of long-term complications and concerns about managing the condition is a significant burden for patients and healthcare professionals. It is well established in the literature that lowering of HbA1c to <7% reduces microvascular and neuropathic complications of type 1 and 2 diabetes (5), however many patients are currently unable to achieve these targets with the available treatments and are therefore at higher risk of suffering from long term complications including diabetic nephropathy, which can in turn make diabetes management more challenging.

Diabetic nephropathy is the principal cause of end-stage renal disease (ESRD) accounting for 25% of incident cases in the UK (6). The number of adults with type 2 diabetes (T2D) is increasing and therefore the incidence of ESRD associated with diabetes is predicted to rise, with even more patients with T2D requiring renal replacement therapy with haemo- or peritoneal dialysis (HD or PD).

People with diabetes requiring dialysis are a vulnerable group, at high risk of adverse outcomes, with cardiovascular events the leading cause of mortality in this population. The overall survival in patients with diabetes on maintenance HD is approximately half that of their non-diabetic peers (3.7 vs. 7 years) (6).

The diabetes management of patients requiring dialysis is complex for both patients and health care professionals, but guidance on glycaemic targets and management algorithms is lacking. There is a clear need for improved delivery of care and novel approaches to the management of diabetes for people requiring dialysis. Phase 1 of this study will look at this group in particular to explore the use of a closed- loop system for diabetes management.

Phase 2 will consist of a more general approach to study closed loop insulin delivery in patients with T2D not requiring maintenance dialysis. The use of closed-loop and CGM technology to enable safe intensification of glucose management in this group is a promising and realistic therapeutic approach.

With the rising prevalence of T2D across all age groups, it is imperative that therapies are optimised to achieve the best possible blood glucose control in an effort to prevent long term complications of the disease.

3.2 Management of type 2 diabetes in patients not requiring dialysis

Lifestyle interventions have shown an element of reversibility in some patients with T2D and is key to reducing cardiovascular risk (7) however the majority of patients with T2D ultimately require a combination of lifestyle interventions and pharmacological therapy as the disease progresses.

An increasing number of oral anti-hyperglycaemic agents have become available to target different mechanisms driving hyperglycaemia in T2D. Unfortunately side effect profiles, contraindications, or the nature of the disease itself often results in the need to intensify treatment to insulin therapy (8). Indeed, in the UK Prospective Study (UKPDS), over 50% of the patients with newly diagnosed T2D required additional insulin therapy alongside oral anti-hyperglycaemic agents within six years (9). Treatment with insulin in T2D has been shown to be effective with respect to improving glycaemic control, however can lead to hypoglycaemia and other associated adverse outcomes including arrhythmias and hypoglycaemia unawareness (10). These limitations to insulin therapy may provide an explanation for the fact that only approximately 50% of patients with T2D reach their target HbA1c (2).

3.3 Management of type 2 diabetes in patients requiring dialysis

Many oral anti-hyperglycaemia therapies are contraindicated in people with diabetes and ESRD, and insulin use is common in this population. There are important changes to both glucose and insulin metabolism that occur with dialysis. Once glomerular filtration rate (GFR) is sufficiently low, insulin clearance becomes markedly reduced, leading to higher levels of circulating insulin (11). The loss of clearance of insulin and reduction in gluconeogenesis in the kidneys leads to falling insulin requirements and subsequently, to a higher risk of hypoglycaemia if insulin is not adjusted (12). Uraemia-induced anorexia and weight loss may occur, reducing insulin requirements.

Dialysis significantly improves insulin sensitivity by removing uraemic toxins (13) and people with diabetes using insulin are often dialysed against a dialysate containing supra-physiological glucose concentrations to mitigate against intra-dialytic hypoglycaemia.

Day to day variability in glucose levels and insulin requirements is high and therefore achieving glucose control can be very challenging. At present, basal bolus insulin regimens are the most flexible and often best suited to the glycaemic variability seen in patients with diabetes requiring maintenance haemodialysis; however demand a significant degree of self-management.

Regular glucose monitoring is essential for the assessment of glycaemic control in patients with diabetes on maintenance dialysis receiving insulin therapy. Hypoglycaemia can be a frequent occurrence, and symptoms of hypoglycaemia are often less pronounced in individuals requiring haemodialysis (hypoglycaemia unawareness). At present, individuals are required to undertake regular self-monitoring with finger-stick blood glucose measurements.

Continuous glucose monitoring (CGM) can provide an accurate assessment of glucose excursions in people with type 2 diabetes requiring haemodialysis (14, 15). CGM provides information on short-term glucose fluctuations associated with dialysis. Studies have shown that using CGM data to guide diabetes treatment significantly improves HbA1c and diabetes control (16).

3.4 Glycaemic control in people with type 2 diabetes requiring dialysis

Fear of hypoglycaemia, in addition to monitoring difficulties, complexity regarding the use of available treatments and therapeutic inertia means that glucose levels are often significantly above the usual glucose targets for individuals with type 2 diabetes requiring dialysis. In the short-term, patients on maintenance haemodialysis with diabetes are more likely to be able to maintain lower intra-dialytic weight gain if glucose control is optimised.

The long-term clinical benefits from maintaining effective glycaemic control in diabetes before ESRD is established and renal replacement therapy is required are well known (17, 18). The benefits of improving chronic hyperglycaemia at the stage of haemodialysis are less clear. Observational studies have shown that better glycaemic control predicts better survival among patients with diabetes on maintenance HD (19, 20). However, the threshold defining good glycaemic control was HbA1c <7.5% (58 mmol/mol), which is higher than conventional target HbA1c values. A one-year

follow-up study of 23,000 subjects with diabetes suggested that low HbA1c levels may not confer survival benefit in ESRD (21). However, the association between poor glycaemic control and greater survival may be explained by confounding from factors such as malnutrition and anaemia (22). Overall, higher HbA1c was associated with increased mortality risk and lower HbA1c levels not related to malnutrition or anaemia appeared to be associated with improved survival in patients on maintenance HD.

In a prospective study in 444 patients on renal replacement therapy (HD or PD), glycated albumin (GA) and HbA1c levels were measured longitudinally (23). For each 5% increase in GA, the risk of death increased by 14%, whilst HbA1c did not predict survival. Restricting the analysis to the patients on haemodialysis, GA significantly predicted risk of death after adjustment for age, gender, race, and BMI, whereas HbA1c did not. A study in 9,201 haemodialysis patients with type 1 or type 2 diabetes showed that mortality was lowest at HbA1c 53–63 mmol/mol (7.0–7.9%) and increased progressively for either lower or higher HbA1c levels (24).

Attempts to intensify glycaemic control have the potential to increase mortality associated with severe hypoglycaemia (25). The increased risk of hypoglycaemia associated with conventional insulin therapy used to achieve tight glycaemic control limits treatment intensification. With current management strategies, it is often necessary for target HbA1c levels among patients with diabetes requiring haemodialysis to be less stringent than levels recommended for other people with diabetes.

3.5 Hypoglycaemia in people with type 2 diabetes requiring dialysis

People with diabetes and chronic kidney disease (CKD) have twice the frequency of hypoglycaemia episodes than people with diabetes who do not have CKD (10.7 vs 5.3 episodes per 100 patient-months, respectively) (26). People with diabetes requiring haemodialysis are at even higher risk of hypoglycaemia. Severe hypoglycaemia, particularly nocturnal episodes, is also more common in individuals with ESRD due to reduced hypoglycaemia awareness, and is associated with arrhythmias.

Among patients admitted to hospital with hypoglycaemia, those with ESRD had a higher mortality rate, longer length-of-stay (LOS) and higher hospitalization costs compared to those without ESRD.

In multivariate analysis, ESRD was significantly associated with increased odds for mortality (OR 2.92, 95% CI 1.98, 4.29, p<0.01), longer LOS (p<0.001) and higher hospitalization costs (p<0.001) (27).

Fear of hypoglycaemia for both patients and healthcare professionals is common and can impact quality of life and lead to suboptimal glucose control. Avoiding hypoglycaemia remains a priority in this vulnerable population. For these reasons, phase 1 of this trial will concentrate on the use of closed loop technology in this group. It is hoped that patients with T2DM and ESRD may derive additional benefits from this treatment, particularly in relation to safer insulin delivery and enabling further intensification of treatment by healthcare professionals.

3.6 Glycaemic targets in people with diabetes requiring dialysis

Glycated haemoglobin (HbA1c) measurement is the main biomarker for assessing glycaemic control in patients with diabetes and renal impairment, however, the accuracy of HbA1c values is poor due to the impact of anaemia and iron deficiency, elevated blood urea nitrogen levels, and uraemia. The relationship between HbA1c and average glycaemia has not been confirmed in patients receiving haemodialysis. HbA1c levels underestimate average blood glucose in patients on maintenance haemodialysis, so the quality of glycaemic control is overestimated, especially in patients with good to moderate glycaemic control.

Glycated albumin (GA) may offer a better opportunity to assess glycaemic control over a shorter time period (15–20 days) and with greater accuracy in patients with diabetes on maintenance haemodialysis. While HbA1c is affected by haemoglobin concentrations and erythropoietin dosage, these factors and serum albumin concentration do not significantly impact GA. In best-fit multivariate models, haemodialysis status significantly impacted HbA1c levels, without significant effect on GA (28-30). GA more accurately reflects recent glycaemic control.

National clinical guidelines do not distinguish between glycaemic targets for those with or without diabetic nephropathy (31, 32). Consensus groups have extrapolated from general recommendations, such as with Kidney Disease Outcomes Quality Initiative (KDOQI) in 2012, which suggested a target HbA1c level of 7% (53 mmol/mol) in those with CKD (33).

The Joint British Diabetes Societies (JBDS) and Association of British Clinical Diabetologists suggest a target HbA1c of 7.5-8.4% (58–68 mmol/mol) in patients with diabetes who require haemodialysis, given the hypoglycaemic and cardiovascular safety considerations (34, 35).

3.7 Glycaemic targets in people with diabetes not requiring dialysis

Current National Institute for Health and Care Excellence (NICE) guidelines support a HbA1c target of 48mmol/mol (6.5%) for adults with T2DM managed by lifestyle and diet ± a single drug not associated with hypoglycaemia. A more relaxed target of 53mmol/mol (7%) is in place for patients on a drug associated with hypoglycaemia. Intensification of therapy is recommended at HbA1c 58mmol/mol (7.5%) or above and it is at this stage that insulin is considered (31)]

A ten year follow up of the UKPDS study showed continued reduction in microvascular risk with intensive glucose management compared to conventional dietary therapy in patients with T2D (36) therefore glucose lowering has remained central to management guidelines. The traditional aim to achieve 'near normal' HbA1c in patients with T2D, however, was challenged in the ACCORD (Action to Control Cardiovascular Risk in Diabetes) and VADT (Veterans Affairs Diabetes) trials, where there was no significant reduction seen in cardiovascular events and increased mortality with intensive therapy (target HbA1c <6%) compared to standard therapy. Among the postulated causes for this included a higher incidence of severe hypoglycaemic events along with weight gain and the use of multiple drugs to achieve this level of control in the ACCORD trial. Interestingly, patients in the ADVANCE (Action in Diabetes and Vascular Disease: Preterax and Diamicron MR Controlled Evaluation) trial achieved a similar median HbA1c to ACCORD within the intensive treatment arm but with no increase in mortality and a key difference noted between the two studies was a higher rate of severe hypoglycaemia in the latter (3% vs 16%) (5), suggesting that avoidance of hypoglycaemia is crucial when implementing insulin therapy.

A meta-analysis carried out in 2015 showed that, on average, a patient with T2DM on insulin experiences 23 episodes of mild-moderate hypoglycaemia (defined as no third-party assistance required) and 1 episode of severe hypoglycaemia (requiring third-party assistance), per year. This was higher than patients who were on oral agents alone (37). A closed-loop system with continuous glucose monitoring (GCM) and integrated alarm systems for hypo- and hyperglycaemia would represent a major step towards safer insulin administration in this patient group.

3.8 Closed-Loop Insulin Delivery

The emergence of new technologies including CGM (38), sensor augmented pump therapy (SAP) (39), and threshold pump suspend (40, 41) provides new opportunities to improve outcomes in diabetes. The most promising approach is closed-loop insulin therapy (42) which combines real-time CGM with insulin pump therapy to achieve glucose-responsive subcutaneous insulin delivery. The vital component of such a system, also known as an artificial pancreas (AP), is a computer-based algorithm. The role of the control algorithm is to compute the amount of insulin to be delivered by the pump using the real-time sensor glucose levels.

The closed-loop approach has been successfully evaluated in children and adults with type 1 diabetes in controlled laboratory studies (43-45) and in home settings (46-51). The results demonstrated improved glucose control and reduced risk of hypoglycaemia events. Psychosocial assessments supported acceptability and positive impact of this novel therapeutic approach. A fully closed-loop approach to the management of type 2 diabetes has been evaluated in the inpatient setting (52, 53), with results suggesting that this technology is a tangible option to improve glucose control in this population.

3.9 Closed-Loop Research in Cambridge

The University of Cambridge and collaborators have a considerable track record investigating closed-loop glucose control in young children, older children, adolescents, adults, and pregnant women with type 1 diabetes (46, 54-57). Since 2012, the University of Cambridge with collaborators have enrolled over 180 subjects in RCTs of free-living closed loop home conditions lasting 1 week to 2 years focusing on young people.

3.9.1 Preclinical testing of Cambridge closed-loop algorithm

The research conducted at the University of Cambridge focused on developing a closed loop system for overnight glucose (initial approach) and day-and-night control (more recent applications; see below) in subjects with T1D. Studies that have been performed employed model predictive control (MPC) – this algorithm estimates user-specific parameters from CGM measurements taken every 1 to 15 minutes and makes predictions of glucose excursions, which are then used to direct insulin infusion between meals and overnight whilst standard bolus calculator is used to deliver prandial insulin (58).

The MPC algorithm has been studied extensively using *in silico* testing utilising a simulator developed by members of the study team (59). The simulations suggested a reduced risk of nocturnal hypoglycaemia and hyperglycaemia with the use of the MPC algorithm (60).

3.9.2 Studies of closed-loop in children and adolescents with type 1 diabetes in the clinical research facility

To date around sixty children and adolescents with type 1 diabetes have been studied at the clinical research facility. Closed-loop insulin delivery was maintained on more than 100 nights. No episodes of significant hypoglycaemia (plasma glucose concentration less than 2.8 mmol/l) have been observed thus far during closed-loop blood glucose control. Results from these studies were published in The Lancet (43) and showed that overnight closed loop therapy increased the time spent euglycaemic by 37% and reduced the risk of overnight hypoglycaemia eight-fold, as compared to conventional pump treatment. Different real-life scenarios predisposing to nocturnal hypoglycaemia, such as afternoon exercise, were explored and closed-loop therapy reduced the risk of overnight hypoglycaemia as compared to conventional insulin pump therapy in a randomised, cross-over design.

3.9.3 Studies of closed-loop in adults with type 1 diabetes in the clinical research facility

We have completed two randomised overnight closed-loop studies in 24 adults with T1D, testing a similar closed-loop system comprising CGM and pump devices and the MPC algorithm. The first study (n=12) assessed the feasibility and efficacy of overnight closed-loop insulin delivery following a moderate-sized (60g carbohydrate) evening meal compared with conventional pump therapy. We demonstrated that overnight closed-loop insulin delivery, compared with usual continuous subcutaneous insulin infusion (CSII), significantly increased time in target plasma glucose range (3.9-8 mmol/l) by 24% and reduced glycaemic variability as measured by standard deviation of plasma glucose. The improvements in glucose control seen on closed-loop were even greater after midnight, when time in target increased by 41%. In the second study we tested the efficacy of overnight closed-loop following a common situation such as consuming a large (100g carbohydrate) evening meal and drinking alcohol (0.75g ethanol/kg body weight of 13%abv white wine). We showed that overnight closed-loop insulin delivery, compared with conventional CSII, similarly increased time in target plasma glucose between 3.9 and 8.0 mmol/l by 24% and reduced time spent above target by 11%, even following such challenges. Importantly these improvements during

closed-loop were achieved with no increased requirement in the average rate of insulin infusion overnight. These results have been published in the British Medical Journal (61).

3.9.4 Overnight closed-loop study in children and adolescents with type 1 diabetes in home setting

Following successful demonstration of safety and efficacy of closed-loop insulin delivery in the research facility, overnight closed-loop studies under free living conditions were commenced in July 2012. The first study compared the efficacy and safety of closed-loop with sensor augmented pump therapy in 16 adolescents over a three week duration (46). Closed-loop was activated over at least 4 hours on 269 nights (80%); sensor data were collected over at least 4 hours on 282 control nights (84%). Closed-loop increased the time when glucose was in target range by a median 15% (interquartile range −9 to +43), P<0.001. Mean overnight glucose was reduced by a mean 0.8±3.2 mmol/l, P<0.001. Time when glucose was below 3.9 mmol/l was low in both groups but nights with glucose below 3.5mmol/l for at least 20min were less frequent during closed-loop (10% vs. 17%, P=0.01). Despite lower total daily insulin doses by a median 2.3 (interquartile range -4.7 to +9.3) units, P=0.009, overall 24h glucose was reduced by a mean 0.5 (standard deviation 2.3 mmol/l (P=0.006) during closed-loop.

In a second multicentre, crossover, randomised, controlled study, we compared 12 week use of an overnight closed-loop insulin delivery system with sensor augmented pump therapy in children and adolescents aged 6 to 18 years (47). The proportion of time with the night-time glucose level in the target range (3.9 to 8.0 mmol/l) was higher during the closed-loop phase than during the control phase (by 24.7 percentage points; 95% CI, 20.6 to 28.7; P<0.001), and the mean night-time glucose level was lower (difference, −1.6 mmol/l; 95% CI,−2.2 to −1.1; P<0.001). The area under the curve for the period in which the day-and-night glucose levels were less than 3.5 mmol/l was lower by 42% (95% CI, 4 to 65; P = 0.03). Two severe hypoglycaemic episodes occurred during the closed-loop phase when the closed-loop system was not in use.

3.9.5 Overnight closed-loop studies in adults with type 1 diabetes in home setting

A four week overnight closed-loop study under free living conditions in 24 adults with type 1 diabetes on insulin pump therapy in a multicentre crossover study design was completed in 2014 (51). Closed-loop was utilised over median 8.3 (interquartile range 6.0, 9.6) hours on 555 nights (86%). The proportion of time when overnight glucose was in the overnight target range between 3.9 and 8.0 mmol/l from midnight to 07:00 was significantly higher during closed-loop compared to sensor

augmented pump therapy (52.6%±10.6 vs. 39.1%±12.8, mean±SD; p<0.001). Mean overnight glucose (8.2±0.9 vs. 9.0±1.3 mmol/l, p=0.005) and time spent above target (44.3%±11.9 vs. 57.1%±15.6, p=0.001) were significantly lower during closed-loop. Time spent below target was low and comparable between interventions [1.8%(0.6, 3.6) vs. 2.1%(0.7, 3.9), p=0.28].

3.9.6 Day-and-night closed-loop studies in adolescents with type 1 diabetes in home setting

We completed a randomised, crossover design study in adolescents aged 10 to 18 years who underwent two 7-day home periods of sensor-augmented insulin pump therapy or closed-loop insulin delivery without supervision or remote monitoring (49). The proportion of time when the sensor glucose level was in the target range (3.9–10 mmol/L) was increased during closed-loop insulin delivery compared with sensor-augmented pump therapy (72% vs. 53%, P < 0.001; primary end point), the mean glucose concentration was lowered (8.7 vs. 10.1 mmol/L, P = 0.028), and the time spent above the target level was reduced (P = 0.005) without changing the total daily insulin amount (P = 0.55). The time spent in the hypoglycaemic range was low and comparable between interventions. A three week single centre study in children and adolescents has also been completed (N = 12).

3.9.7 Day and night closed-loop studies in adults with type 1 diabetes in home setting

In 2014, we completed a first study testing a day and night home system over a seven day period in 17 adults. This randomised clinical trial adopted a multicentre, multi-national, crossover design. During the home phase, the percentage time when glucose was in target range (3.9 to 10.0 mmol/l) was significantly higher during closed loop compared to sensor augmented pump therapy (75 [61, 79] vs. 62 [53, 70]%, median [IQR], p=0.005). Mean glucose (8.1 vs. 8.8 mmol/l, p=0.027) and time spent above target (p=0.013) were lower during closed-loop while time spent below target was comparable (p=0.339). Increased time in target was observed during both day-time (p=0.017) and night-time (p=0.013).

We completed a multicentre, multinational, crossover, randomised, controlled study under free living home conditions comparing 24/7 closed-loop insulin delivery with sensor augmented pump therapy (control intervention) in 33 adults with type 1 diabetes (47). The proportion of time that the glucose level was in the target range (3.9 to 10.0 mmol/l) was 11.0 percentage points (95% confidence interval [CI], 8.1 to 13.8) greater with the use of the closed-loop system day and night than with

control therapy (P<0.001). The mean glucose level was lower during the closed-loop phase than during the control phase (difference, −0.6 mmol/l; 95% CI, −0.9 to −0.3; P<0.001), as were the area under the curve for the period when the glucose level was less than 3.5 mmol/l (39% lower; 95% CI, 24 to 51; P<0.001) and the mean glycated haemoglobin level (difference, −0.3%; 95% CI, −0.5 to −0.1; P = 0.002).

3.9.8 Closed-loop studies in adults with type 2 diabetes

The Cambridge closed-loop system has been shown to be safe and feasible in insulin-naive patients with type 2 diabetes in a controlled research facility setting (62).

We have previously assessed fully automated closed-loop insulin delivery in non-critical care patients with type 2 diabetes hospitalised in the general wards (63). Forty participants were randomised to either automated fully closed-loop insulin delivery or usual insulin therapy for a 72h study period. Results showed that closed-loop significantly increased time spent within target glucose range (5.6-10.0mmol/l), without any increase in the risk of hypoglycaemia. Closed-loop insulin delivery without meal-time boluses is effective and safe in insulin-treated adults with type 2 diabetes.

In a larger multi-national study, 136 adults with type 2 diabetes who required insulin therapy were randomised to receive either closed-loop insulin delivery or conventional subcutaneous insulin therapy for up to 15 days or until hospital discharge (53). The mean percentage of time that the sensor glucose measurement was in the target range was 65.8% in the closed-loop group and 41.5% in the control group, a difference of 24.3 percentage points (P<0.001); values above the target range were found in 23.6% and 49.5% of the patients, respectively, a difference of 25.9 percentage points (P<0.001). The mean glucose level was 8.5 mmol/l in the closed-loop group and 10.4 mmol/l in the control group (P<0.001). There was no significant between-group difference in the duration of hypoglycaemia or in the amount of insulin that was delivered. Among inpatients with type 2 diabetes receiving noncritical care, the use of an automated, closed-loop insulin-delivery system resulted in significantly better glycaemic control than conventional subcutaneous insulin therapy, without a higher risk of hypoglycaemia.

3.10 Risk and benefits

A potential key benefit of closed-loop insulin delivery is a reduction in hypoglycaemia which has been shown to be associated with cardiovascular events, increased hospital admissions and increased mortality. The long-term impact of improved glucose control in this population may be reduced rates of diabetes complications and improved quality of life.

Any potential risks presented by this investigation have been minimized and adequate testing, safeguards, and safety monitoring will be incorporated into the investigation to further minimize and mitigate these risks. A detailed Risk Management File adopting risk management processes complying with EN ISO 14971:2012 Medical Devices – Application of Risk Management to Medical Devices, will be submitted as part of the regulatory submission to the MHRA.

3.11 CamAPS HX fully-automated closed loop system to be used in the present study

In the present study, we will use the CamAPS HX closed-loop system comprising:

- Dana insulin pump (Diabecare, Sooil, Seoul, South Korea)
- Dexcom G6 real-time CGM sensor (Dexcom, Northridge, CA, USA)
- An Android smartphone hosting CamAPS HX Application with the Cambridge model predictive control algorithm and communicating wirelessly with the insulin pump
- Cloud upload system to monitor CGM/insulin data.

An overview of this proposed automated closed loop system is given in Figure 1.

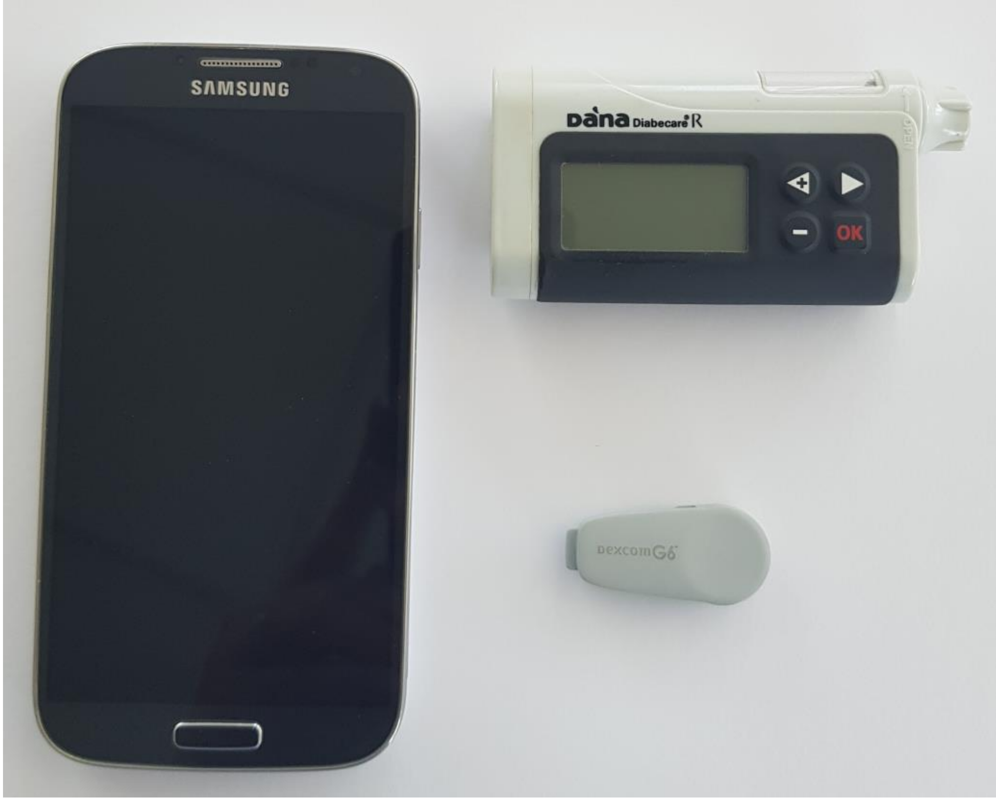

Figure 1: CamAPS HX comprises Samsung Galaxy phone (or similar) running Cambridge control algorithm, Dana insulin pump (Sooil), G6 real-time CGM sensor (Dexcom).

3.12 Rationale for the present study

The study builds on recent technological advances of closed-loop insulin delivery (artificial pancreas). Studies from our group have assessed the safety and efficacy of closed-loop insulin delivery in T1D in a controlled research setting and at home. Closed-loop use in hospitalised patients has been evaluated in the intensive care setting and on the general wards, and has demonstrated efficacy and safety in achieving target glucose range when compared to standard treatment (52, 64-67). Despite studies in people with type 2 diabetes, including those requiring maintenance dialysis, reporting an adverse relationship between dysglycaemia and clinical outcome, glycaemic management of these patients remains suboptimal.

Closed-loop insulin delivery may be of benefit to such patients in whom the optimal dosing regimen is difficult to establish, and hence may be better facilitated by an algorithm-initiated and driven insulin therapy. Most importantly, closed-loop may provide a safer method of insulin delivery with the added benefit of continuous monitoring of glucose levels, thus minimising the likelihood of hyper- and hypoglycaemic events and their known associated worse outcomes.

The purpose of phase 1 of this study is to test the impact of closed loop insulin delivery in patients with type 2 diabetes requiring maintenance dialysis on time in target glucose range and frequency of hypoglycaemia. This patient group has been selected due to the range of challenges associated with insulin use and glycaemic control as a result of their renal disease. Compared to patients with T2DM who do not require renal replacement therapy, there is a greater requirement for safer insulin delivery and careful monitoring of blood glucose in this group, therefore it is foreseeable that this group may derive additional benefit from the closed-loop system.

Phase 2 of the trial will comprise a more generalisable approach to include the wider population of patients with T2D who are not on renal replacement therapy. It is well established that there is progress to be made in safely intensifying insulin treatment in this group also, and closed loop technology offers hope that this can be achieved. The feasibility and acceptance of this therapy will be assessed so that it could be considered as a standard treatment modality in the future.

4 Objectives

4.1 Efficacy

To assess efficacy of day-and-night fully automated closed-loop insulin delivery in maintaining glucose levels within the target range from 5.6 to 10.0 mmol/l in phase 1 and from 3.9- 10.0 mmol/l in phase 2 based on subcutaneous continuous glucose monitoring (CGM), during the 20 day (phase 1) and 8 week (phase 2) home stay as compared to usual care.

4.2 Safety

To evaluate the safety of fully-automated closed-loop glucose control in terms of episodes and severity of hypoglycaemia and nature and severity of other adverse events.

4.3 Utility

To determine the acceptability, duration and frequency of use of the closed-loop system.

5 Study design

An open-label, two-centre (phase 1) and single centre (phase 2), randomised, two-period crossover study assessing the efficacy, safety and utility of fully automated closed-loop insulin delivery compared with usual care in adults with type 2 diabetes with and without a requirement for maintenance dialysis (phase 1 and 2, respectively). Two intervention periods in the home setting will last 20 days each (phase 1) and 8 weeks each (phase 2) with 2-4 weeks washout period. The order of the two interventions will be random.

Phase 1 will recruit up to 40 adults with T2D requiring maintenance dialysis, to allow for 32 completed subjects available for assessment. Phase 2 will recruit up to 30 adults with T2D and no requirement for dialysis, to allow for 24 completed subjects available for assessment.

The study flow chart is outlined in Figure 2.

Figure 2: Study flow chart

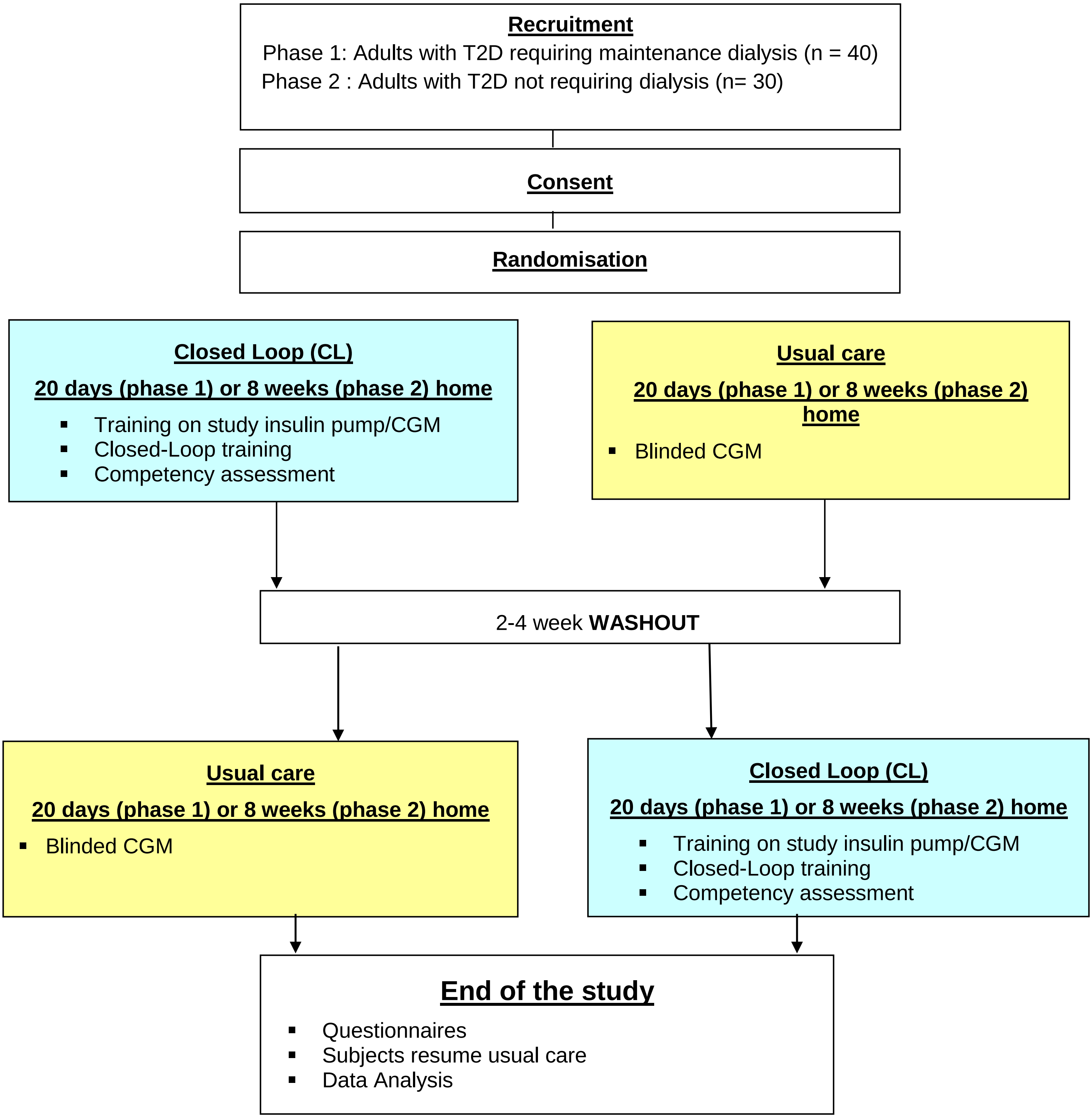

6 Study participants

6.1 Study population

Adults with type 2 diabetes will be recruited (phase 1 and 2). Phase 1 will be limited to adults with T2D requiring maintenance dialysis.

6.1.1 Inclusion criteria

1. The subject is age 18 years or over
2. Diagnosis of type 2 diabetes using standard diagnostic practice
3. The subject requires maintenance dialysis (phase 1 only)
4. The subject requires current treatment with subcutaneous insulin
5. Screening HbA1c ≤ 12% (108mmol/mol) on analysis from local laboratory
6. Subject is willing to perform regular finger-prick blood glucose monitoring
7. The subject is literate in English (UK) (phase 1 and 2) or German (Switzerland) (phase 1)
8. The subject is willing to wear study devices 24/7 during intervention arm and follow study specific instructions

6.1.2 Exclusion criteria

1. Physical or psychological condition likely to interfere with the normal conduct of the study and interpretation of the study results as judged by the investigator
2. Known or suspected allergy to insulin
3. Lack of reliable telephone facility for contact
4. Pregnancy, planned pregnancy, or breast feeding
5. Severe visual impairment
6. Severe hearing impairment
7. Medically documented allergy towards the adhesive (glue) of plasters or unable to tolerate tape adhesive in the area of sensor placement
8. Serious skin diseases located at places of the body, which potentially are possible to be used for localisation of the glucose sensor
9. Illicit drugs abuse
10. Prescription drugs abuse
11. Alcohol abuse

6.2 Recruitment and informed consent

The study will aim for 32 and 24 completed subjects in phase 1 and 2, respectively. Recruitment will target up to 40 subjects (phase 1) and 30 subjects (phase 2) to allow for drop-outs. Participants will be recruited from the adult diabetes outpatient clinics, GP practices within the West Suffolk, Cambridge and Peterborough Clinical Commissioning Groups (CCG) or from the dialysis units at the following centres.

1. Addenbrooke’s Hospital, Cambridge University Hospitals NHS Foundation Trust, Cambridge, UK (phase 1 and 2)
2. Bern University Hospital, Bern, Switzerland (phase 1 only)

Potential participants will be identified by their treating clinicians and a contact with the research team will be established if agreed. Study information leaflets and/or similar recruitment material will be handed out or sent to participants by the research team including an invitation to join the study. Written informed consent will be obtained from all participants before any study related activities.

7 Methods under investigation

7.1 Name and description of the method of investigation

The investigational treatment is a closed-loop system, see section 3.11, or follow up prototypes of the automated closed-loop insulin delivery system manufactured by the Cambridge University Hospitals NHS Foundation Trust. Component versions will be identified during regulatory submission to the national regulatory bodies.

7.2 Intended purpose

The intended purpose of the investigational treatment is automated day and night fully closed-loop insulin delivery. The investigated medical device is used to manage glucose levels in adults with type 2 diabetes, using a fully closed-loop approach.

7.3 Method of administration

The closed-loop system consists of components directly attached to the patient, which are the CGM sensor/transmitter and the insulin pump. The component not directly attached to the patient is the

handheld smartphone containing closed-loop algorithm and communicating wirelessly with the insulin pump.

7.4 Required training

Prior to commencement of the study, the research team nurses/clinicians will be trained to use the closed-loop system and its components. Prior to the use of study devices, participants will be trained to use the study CGM device, the study pump and the closed-loop system. Competency assessments of the participants' capability to use study devices and the closed-loop system will be made.

7.5 Precautions

During treatment with insulin there is a risk of hypoglycaemia and hyperglycaemia. In-hospital testing and hazard analysis have documented reduced risk of hypoglycaemia and hyperglycaemia during closed loop compared to conventional treatment.

7.6 Accountability of the method under investigation

The local Investigator will provide training for the study participants and will make every effort, through regular contact, to ascertain that the closed loop system is used for the study purposes only. Devices will be identified using batch/lot/serial numbers and the location of investigational devices and their dates of use by subjects will be documented throughout the study.

8 Study schedule

8.1 Overview

The study will be co-ordinated from the Institute of Metabolic Science, Addenbrooke's Hospital, Cambridge. Participants in the UK will be recruited through the Dialysis unit (phase 1) or GP practices within the West Suffolk, Cambridge and Peterborough Clinical Commissioning Groups (CCG), outpatient diabetes clinics (phase 2) in Addenbrooke's Hospital, Cambridge, and other satellite haemodialysis units in the Cambridgeshire region. Participants in Switzerland will be recruited through the Dialysis unit in University Hospital Bern (phase 1 only).

Over the two study periods (closed-loop vs. usual care), the study will consist of up to 5 visits and 6 contacts. The study periods will last 20 days each for phase 1 and 8 weeks each for phase 2. The

order of the two interventions will be random. There will be a 2-4 week washout period between the two study periods.

Prior to the closed-loop intervention, there will be a training visit, which will be conducted at the CRF/dialysis unit, followed by a 20 day or 8 week study period at the participant's home in phase 1 and 2 respectively. The training visit will last approximately 4 hours and will return home when the participant is competent and confident in using the closed-loop system. Maximum time in study is 12 weeks for phase 1 and 20 weeks for phase 2.

Table 1 outlines study activities when CL intervention precedes standard care in phase 1.

Table 2 outlines study activities when standard care precedes CL intervention in phase 1.

Table 3 outlines study activities when CL intervention precedes standard care in phase 2.

Table 4 outlines study activities when standard care precedes CL intervention in phase 2.

Table 1: Schedule of study visits / phone contacts in phase 1 when closed-loop intervention precedes usual care

	**Visit/ contact**	**Description**	**Start relative to previous / next Visit / Activity**	**Duration**
	Visit 1	Recruitment visit: Consent, baseline assessments including questionnaires	-	1-2 hours
**RANDOMISATION**				
**CL Intervention (20 days)**	Visit 2	CGM, Insulin pump and closed loop training<br>Competency assessment and initiation of closed loop	Within 1 to 3 weeks of Visit 1<br>Training visits can be repeated if competency not achieved	3-4 hours
	Contact 1*	Review use of study devices	24h after Visit 2 (±3 days)	<0.5 hour
	Contact 2*	Review use of study devices	7 days after Visit 2 (±3 days)	<0.5 hour
	Contact 3*	Review use of study devices	14 days after Visit 2 (±3 days)	<0.5 hour
	Visit 3	End of closed-loop treatment period (20 days)<br>Return devices. Revert back to usual diabetes therapy. Questionnaires	After 20 days of Visit 2	1-2 hours
		**Washout period**	Immediately after Visit 3	2-4 weeks
**Usual Care (20 days)**	Visit 4	Blinded CGM insertion<br>Review of diabetes management	Within 2-4 weeks of Visit 3	1-2 hours
	Contact 4*	Review diabetes management	24h after Visit 4 (±3 days)	<0.5 hour

	Contact 5*	Review diabetes management	7 days after Visit 4 (±3 days)	<0.5 hour
	Contact 6*	Review diabetes management.	14 days after Visit 4 (±3 days)	<0.5 hour
	Visit 5	End of usual care period (20 days)<br>Questionnaires	After 20 days of Visit 4	1-2 hours
* could be done at home or phone/email				

Table 2: Schedule of study visits / phone contacts in phase 1 when usual care precedes closed loop intervention

	Visit/ contact	Description	Start relative to previous / next Visit / Activity	Duration
	Visit 1	Recruitment visit: Consent, baseline assessments including questionnaires	-	1-2 hours
**RANDOMISATION**				
**Usual Care (20 days)**	Visit 2	Blinded CGM insertion<br>Review of diabetes management	Within 1-3 weeks of Visit 1	1-2 hours
	Contact 1*	Review diabetes management	24h after Visit 2 (±3 days)	<0.5 hour
	Contact 2*	Review diabetes management	7 days after Visit 2 (±3 days)	<0.5 hour
	Contact 3*	Review diabetes management	14 days after Visit 2 (±3 days)	<0.5 hour
	Visit 3	End of usual care period (20 days)<br>Questionnaires	After 20 days of Visit 2	1-2 hours
		**Washout period**	Immediately after Visit 3	2-4 weeks
**CL Intervention (20 days)**	Visit 4	CGM, Insulin pump and closed loop training<br>Competency assessment and initiation of closed loop	Within 2 to 4 weeks of Visit 3<br>Training visits can be repeated if competency not achieved	3-4 hours
	Contact 4*	Review use of study devices	24h after Visit 4 (±3 days)	<0.5 hour

	Contact 5*	Review use of study devices	7 days after Visit 4 (±3 days)	<0.5 hour
	Contact 6*	Review use of study devices	14 days after Visit 4 (±3 days)	<0.5 hour
	Visit 5	End of closed-loop treatment period (20 days)<br>Return devices. Revert back to usual diabetes therapy. Questionnaires	After 20 days of Visit 4	1-2 hours
* could be done at home or phone/email				

Table 3. Schedule of study visits / phone contacts in phase 2 when closed loop intervention precedes usual care.

	Visit/ contact	Description	Start relative to previous / next Visit / Activity	Duration
	Visit 1	Recruitment visit: Consent, baseline assessments including questionnaires<br>Hba1c measurement	-	1-2 hours
**RANDOMISATION**				
**CL Intervention (8 weeks)**	Visit 2	CGM, Insulin pump and closed loop training<br>Competency assessment and initiation of closed loop	Within 1 to 3 weeks of Visit 1<br>Training visits can be repeated if competency not achieved	3-4 hours
	Contact 1*	Review use of study devices	24h after Visit 2 (±3 days)	<0.5 hour
	Contact 2*	Review use of study devices	7 days after Visit 2 (±3 days)	<0.5 hour
	Contact 3*	Review use of study devices	4 weeks after Visit 2 (3 days)	<0.5 hour
	Visit 3	End of closed-loop treatment period (8 weeks)<br>Return devices. Revert back to usual diabetes therapy. Questionnaires<br>HbA1c measurement	After 8 weeks of Visit 2	1 hour
		**Washout period**	Immediately after Visit 3	2-4 weeks

Usual Care (8 weeks)	Visit 4	Blinded CGM insertion<br>Review of diabetes management	Within 2-4 weeks of Visit 3	1-2 hours
	Contact 4*	Review diabetes management	24h after Visit 4 (±3 days)	<0.5 hour
	Contact 5*	Review diabetes management	7 days after Visit 4 (±3 days)	<0.5 hour
	Contact 6*	Review diabetes management.	4 weeks after Visit 4 (±3 days)	<0.5 hour
	Visit 5	End of usual care period (8 weeks)<br>Questionnaires<br>HbA1c measurement	After 8 weeks of Visit 4	1 hour
* could be done at home or phone/email				

Table 4. Schedule of study visits / phone contacts in phase 2 when usual care precedes closed-loop intervention

	Visit/ contact	Description	Start relative to previous / next Visit / Activity	Duration
	Visit 1	Recruitment visit: Consent, baseline assessments including questionnaires<br>HbA1c measurement	-	1-2 hours
**RANDOMISATION**				
Usual Care (8 weeks)	Visit 2	Blinded CGM insertion<br>Review of diabetes management	Within 1 to 3 weeks of Visit 1	1-2 hours
	Contact 1*	Review diabetes management	24h after Visit 2 (±3 days)	<0.5 hour
	Contact 2*	Review diabetes management	7 days after Visit 2 (±3 days)	<0.5 hour
	Contact 3*	Review diabetes management.	4 weeks after Visit 2 (±3 days)	<0.5 hour
	Visit 3	End of usual care period (8 weeks<br>Questionnaires<br>HbA1c measurement	After 8 weeks of Visit 2	1-2 hours
		**Washout period**	Immediately after Visit 3	2-4 weeks

**CL Intervention (8 weeks)**	Visit 4	CGM, Insulin pump and closed loop training<br>Competency assessment and initiation of closed loop	Within 2 to 4 weeks of Visit 3<br>Training visits can be repeated if competency not achieved	3-4 hours
	Contact 4*	Review use of study devices	24h after Visit 4 (±3 days)	<0.5 hour
	Contact 5*	Review use of study devices	7 days after Visit 4 (±3 days)	<0.5 hour
	Contact 6*	Review use of study devices	4 weeks after Visit 4 (±3 days)	<0.5 hour
	Visit 5	End of closed-loop treatment period (8 weeks)<br>Return devices. Revert back to usual diabetes therapy. Questionnaires<br>HbA1c measurement	After 8 weeks of Visit 4	1-2 hours
* could be done at home or phone/email				

Baseline visit (Visit 1)

Once participants have agreed to participate in the study, they will be invited for the baseline visit, when the following activities will be performed by the research team:

- written informed consent
- checking inclusion and exclusion criteria
- medical (diabetes) history
- record of current insulin therapy
- body weight measurement
- questionnaires will be distributed to assess quality of life and diabetes management.
- HbA1c measurement

Switzerland only (phase 1)

Woman of reproductive age will be required to take a pregnancy test and will be advised to use contraception during study participation.

8.2 Randomisation

On completion of Visit 1, eligible participants will be randomised in a 1:1 ratio using randomisation software to the initial use of fully automated closed-loop glucose control or to usual care for 20 days in phase 1 or 8 weeks in phase 2, with a 2-4 week washout period before crossing over to the second intervention arm.

8.3 Post-randomisation training (Visits 2 and 4)

8.3.1 Closed loop intervention

Participants starting the closed-loop arm will receive training to cover key aspects of insulin pump use and CGM, prior to training on closed-loop insulin delivery. Particular attention will be paid to:

- Insulin cartridge and infusion set changes and correct priming procedure
- Sensor insertion and calibration
- Blood glucose targets and alarm settings
- Hypo- and hyperglycaemia management
- Connection and disconnection of the closed-loop system

Written easy to use guidelines for the operation of insulin pump, CGM and closed-loop will be provided. This session will be conducted by a professional pump educator and/or member of the study team. Device manual guides will be provided.

Competency in the use of study pump, CGM and closed-loop system will be assessed by the study team. Only subjects who demonstrate competency in use of the system will be allowed to continue to the home study phase.

Subjects will be advised to use closed-loop 24/7 for the next 20 days (phase 1) or 8 weeks (phase 2). Written step by step guidance will also be provided, including how to deal with low and high glucose at home. Subjects will be provided with 24 hour telephone helpline and information on when to contact study team.

The subject is allowed to drive while adhering to usual precautions and country specific rules and regulations.

8.3.2 Standard therapy (control intervention)

Participants in the control arm will continue to follow their current diabetes management plan for the 20 day or 8 week study period in phase 1 and 2 respectively. During the control arm, participants and/or the clinical team are free to adjust insulin therapy as per usual clinical practice, but no active treatment optimisation will be undertaken by the study team. For self-monitoring of blood glucose (SMBG) throughout the study, subjects will continue using their own glucose meter provided the meter type meets ISO standards (Standard 15197: 2013).

Participants will be shown how to insert the study CGM and they will be asked to wear a blinded continuous glucose monitoring (CGM) system during the 20 day (phase 1) or 8 week (phase 2) home stay. If the sensor fails or sensor function is interrupted prematurely (detached sensor), another sensor will be inserted. The sensor(s) will be sent back/collected by the research team once the sensor life has expired and/or the sensor has detached.

8.4 Contacts after initiation of study arm

Participants will be contacted 24 hours and 1 and 2 weeks after initiation of the respective study arm (phase 1). Phase 2 will involve contacts at 24 hours, 1 week and monthly thereafter until the end of the 8 week period. These contacts can be via telephone/email. The purpose of this contact would be to troubleshoot any problems, and to record any adverse events, device deficiencies, and changes in insulin therapy.

8.5 End of first study arm visit (Visit 3)

On completion of the first study arm, participants will be invited to attend the research facility/clinical area 20 days (phase 1) or 8 weeks (phase 2) after study arm initiation. Participants will return all study devices and then resume usual care. Participants will be asked to complete questionnaires and a HbA1c sample will be taken.

8.6 Washout

A minimum washout period of 2 weeks must be ensured between treatment periods. Duration of the wash out period has been chosen to minimise any carry over effect between the two interventions. The subject will continue with their usual diabetes care during the washout period. Following the washout period subjects will cross over to alternative intervention.

8.7 End of study visit (Visit 5)

On completion of the second study arm, participants will be invited to attend the research facility/clinical area 20 days (phase 1) or 8 weeks (phase 2) after study arm initiation. Participants will return all study devices and then resume usual care. Participants will be asked to complete questionnaires and a HbA1c sample will be taken.

8.8 Participant withdrawal criteria

The following pre-randomisation withdrawal criterion will apply:

1. Subject is unable to demonstrate safe application of insulin therapy as judged by the investigator

The following pre- and post-randomisation withdrawal criteria will apply:

2. Subject is unable to demonstrate safe use of insulin injections or study insulin pump and/or CGM and/or closed loop during post randomisation training period as judged by the investigator
3. Subjects may terminate participation in the study at any time without necessarily giving a reason and without any personal disadvantage
4. Significant protocol violation or non-compliance
5. Recurrent severe hypoglycaemia events not related to the use of the closed loop system
6. Recurrent severe hyperglycaemia event/DKA unrelated to infusion site failure and related to the use of the closed loop system
7. Decision by the investigator or the Sponsor that termination is in the subject's best medical interest
8. Allergic reaction to insulin
9. Allergic reaction to adhesive surface of infusion set or glucose sensor

Subjects who are withdrawn for reasons stated in (4) to (9) will be invited to complete questionnaires at the end of the planned study intervention. Subjects who discontinue study intervention prior to the final visit will receive an exit survey.

8.9 Study stopping criteria

The study may be stopped if three consecutive participants withdraw from the study, the study may be stopped for safety reasons or on the advice of the Data Safety Monitoring Board.

8.10 Support telephone line

In addition to standard clinical advice, there will be a 24-hour telephone helpline to the research team for subjects in case of any technical device or problems related to diabetes management such as hypo- or hyperglycaemia.

8.11 Subject reimbursement

The study will provide the CGM device, insulin pump, CL components and related consumables. A study payment will be made to reflect local practice. The amount paid will be specified in the participant information sheet/informed consent form and REC application form. Reasonable travel expenses will be reimbursed. After completing the study, subjects will not keep the study devices. They will revert to their usual diabetes management.

9 Endpoints

9.1 Efficacy endpoints

9.1.1 Primary efficacy endpoint

The primary endpoint is the time spent in the target glucose range from 5.6 to 10.0 mmol/l during the 20 day study period for phase 1, and time spent in target glucose range from 3.9 to 10.0 mmol/l during the 8 week study period in phase 2, based on continuous glucose monitoring (CGM).

9.1.2 Other Key Endpoints

- Time spent above target glucose (10.0 mmol/l)
- Average of glucose levels
- HbA1c (phase 2 only)
- Time spent with glucose levels <3.9 mmol/l

9.1.3 Secondary efficacy endpoints

Secondary endpoints include:

- Time spent below target glucose of 5.6 mmol/l for phase 1

- Standard deviation and coefficient of variation of glucose levels
- The time with glucose levels <3.0 mmol/l
- The time with glucose levels > 16.7 mmol/l (phase 2 only)
- The time with glucose levels in significant hyperglycaemia (glucose levels > 20 mmol/l)
- Total daily insulin dose
- AUC of glucose below 3.5mmol/l (phase 1 only)

Endpoints regarding glucose levels will be based on sensor glucose data.

9.1.4 Exploratory endpoints

- Average inter-dialytic weight gain (phase 1 only)

9.2 Safety evaluation

Safety evaluation will comprise the number of episodes of severe hypoglycaemia as well as the number of subjects experiencing severe hypoglycaemia, severe hyperglycaemia (>20 mmol/l) and number, nature and severity of any other adverse events.

All subjects including those who withdraw will be included in the safety evaluation.

9.3 Utility evaluation

Utility evaluation is the frequency and duration of use of the closed-loop system. Expectations, attitudes and responses to the closed-loop system will be assessed using questionnaires.

10 Assessment and reporting of adverse events

10.1 Definitions

10.1.1 Reportable Adverse Events

A reportable Adverse Event is any untoward medical occurrence that meets criteria for a serious adverse event or any unanticipated medical occurrence in a study subject that is study or device-related. Device deficiencies that could have led to a serious adverse device effect will also be reported (ISO 14155: 8.2.5 and 9.8).

10.1.2 Adverse Events

An adverse event (AE) is any untoward medical occurrence, unintended disease or injury, or untoward clinical signs (including abnormal laboratory findings) in a subject who has received an investigational device, whether or not related to the investigational medical device (ISO 14155: 3.2). This definition includes events related to the device under investigation or the comparator or to the study procedures. For users or other persons, this definition is restricted to events related to the investigational device. The following anticipated adverse events will not be recorded:

- Non clinically significant skin reactions as judged by investigator
- Pre-existing medical conditions
- New illnesses or conditions not requiring concomitant medication or medical intervention/procedures
- Non severe hypoglycaemia
- Hyperglycaemia without significant ketonaemia

10.1.3 Adverse Device Effect

An Adverse Device Effect (ADE) is an adverse event related to the use of an investigational medical device (ISO 14155: 3.1). This includes adverse events resulting from insufficient or inadequate instructions for use, deployment, implantation, installation, or operation, or any malfunction of the investigational medical device. This definition also includes any event resulting from use error or from intentional misuse of the device under investigation.

10.1.4 Serious Adverse Event

A serious adverse event (SAE) is an adverse event that:

- led to a death
- led to a serious deterioration in the health of the subject, that either resulted in:
    - a life threatening illness or injury
    - a permanent impairment of a body structure or function
    - in-patient hospitalisation or prolonged hospitalisation
    - medical or surgical intervention to prevent life-threatening illness or injury or permanent impairment to a body structure or a body function
- led to foetal distress, foetal death or a congenital abnormality or birth defect

A planned hospitalisation for pre-existing condition, or a procedure required by the study protocol, without a serious deterioration in health, is not considered to be a serious adverse event.

More than one of the above criteria can be applicable to one event. Life-threatening in the definition of a serious adverse event or serious adverse reaction refers to an event in which the subject was at risk of death at the time of the event; it does not refer to an event which hypothetically might have caused death if it were more severe. Medical judgement should be exercised in deciding whether an adverse event or reaction is serious in other situations.

Important adverse events or reactions that are not immediately life-threatening or do not result in death or hospitalisation but may jeopardise the subject or may require intervention to prevent one of the other outcomes listed in the definition above, should also be considered serious.

The following serious adverse events are anticipated:

- Severe hypoglycaemia
- DKA

10.1.5 Serious Adverse Device Effect

A Serious Adverse Device Effect (SADE) is an adverse device effect that has resulted in any of the consequences characteristic of a serious adverse event.

10.1.6 Unanticipated Serious Adverse Device Effect

An Unanticipated Serious Adverse Device Effect (USADE) is a serious adverse device effect which by its nature, incidence, severity or outcome has not been identified in the current version of the protocol or the risk analysis report (ISO 14155: 3.42).

An Anticipated Serious Adverse Device Effect (ASADE) is a serious adverse device effect which by its nature, incidence, severity or outcome has been identified in the protocol.

10.1.7 Device Deficiencies

A device deficiency is an inadequacy of a medical device with respect to its identity, quality, durability, reliability, safety or performance such as malfunction, misuse or user error and inadequate labelling (ISO 14155: 3.15). A device deficiency may lead to an Adverse Device Effect or Serious Adverse Device Effect. The following anticipated device deficiencies and device-related issues will not be recorded:

- Infusion set occlusion/leakage not leading to ketonaemia
- Sensor failure due to significant over/under-reading (difference>3mM) or detachment

- Premature interruption of sensor-life
- Battery lifespan deficiency due to inadequate charging or extensive wireless communication
- CAD error messages not needing system replacement
- Intermittent device communication failure not leading to system replacement

10.1.8 Adverse event intensity

Intensity	Definition
Mild	Patient is aware of signs and symptoms but they are easily tolerated
Moderate	Signs / symptoms cause sufficient discomfort to interfere with usual activities
Severe	Patient is incapable to work or perform usual activities

NB. The term "severe" is often used to describe the intensity (severity) of a specific event. This is not the same as 'serious', which is based on patient/event outcome or action criteria (see definition 10.1.4). For example, itching for several days may be rated as severe, but may not be clinically serious.

10.1.9 Adverse event causality

Intensity	Definition
Not assessable	A report suggesting an adverse event, which cannot be judged because information is insufficient or contradictory, and which cannot be supplemented or verified.
Unlikely	A clinical event, including laboratory test abnormality, with a temporal relationship, which makes a causal relationship

	improbable, and in which other drugs/treatments, chemicals or underlying disease(s) provide plausible explanations.
Possible	A clinical event, including laboratory test abnormality, with a reasonable time sequence to administration of the treatment/use of investigational treatment/device, but which also could be explained by concomitant diseases or other drugs/treatments or chemicals.
Probable	A clinical event, including laboratory test abnormality, with a reasonable time sequence to administration of the treatment/use of medical method/device, unlikely to be attributable to concomitant disease(s) or other drugs/treatments or chemicals, and which follows a clinically reasonable response on withdrawal (dechallenge). Rechallenge information is not required to fulfil this definition.
Definite/certain	A clinical event, including laboratory test abnormality, occurring in a plausible time relationship to study treatment/use of medical method/device and which cannot be explained by concomitant disease(s), other drugs/treatments or chemicals. The response to withdrawal of the treatment (dechallenge) should be clinically plausible. The event must be unambiguous, either pharmacologically or as phenomenon, using satisfactory rechallenge procedures if necessary.

(Reference: WHO-UMC Causality Categories)

10.2 Recording and reporting of adverse events, serious adverse events and device deficiencies

10.2.1 Monitoring period of adverse events

The period during which adverse events will be reported is defined as the period from the beginning of the study (obtaining informed consent) until 72 hours after the end of the study participation. Adverse events that continue after the subject's discontinuation or completion of the study will be followed until their medical outcome is determined or until no further change in the condition is expected. The follow up of AEs may therefore extend after the end of the clinical investigation; however no new AEs will be reported after the trial reporting period.

10.2.2 Recording and reporting of adverse events

Throughout the course of the study, all efforts will be made to remain alert to possible adverse events or untoward findings. The first concern will be the safety of the subject, and appropriate medical

intervention will be taken. The investigator will elicit reports of adverse events from the subject at each visit and complete adverse event forms. All AEs, including those the subject reports spontaneously, those the investigators observe, and those the subject reports in response to questions will be recorded on paper or electronic AE forms at each site within seven days of discovering the event.

The study investigator will assess the relationship of any adverse event to be device-related or unrelated by determining if there is a reasonable possibility that the adverse event may have been caused by the study device or study procedures. The individual investigator at each site will be responsible for managing all adverse events according to local protocols, and decide if reporting is required.

10.2.3 Severe hypoglycaemia

Severe hypoglycaemia will be defined as an event requiring assistance of another person to actively administer carbohydrate, glucagon, or other resuscitative actions. These episodes may be associated with sufficient neuroglycopaenia to induce seizure or coma. If plasma glucose measurements are not available during such an event, neurological recovery attributable to the restoration of plasma glucose to normal is considered sufficient evidence that the event was induced by a low plasma glucose concentration.

Severe hypoglycaemia will be regarded as a foreseeable adverse event and an adverse event form will be completed. Severe hypoglycaemia is not necessarily a serious adverse event and hence may not require immediate reporting to the Sponsor. Non-severe hypoglycaemia will not be reported or considered an adverse event.

10.2.4 Reporting of serious adverse events and serious adverse device effects

When reporting adverse events, all pertinent data protection legislation must be adhered to.

The serious adverse event report should contain the following information*:

1. Study identifier (EudraCT number if applicable)
2. Participant’s unique study number
3. Date of birth
4. Event description
5. Start date of event

6. Laboratory tests used and medical interventions used to treat the SAE
7. Planned actions relating to the event, including whether the study device was discontinued
8. Statement on the patient's current state of health
9. Criterion for seriousness (i.e. death, life threatening, hospitalisation, disability/incapacity or other)
10. Evaluation of causality (including grade of relatedness) with the following (more than one may apply):
    a. the investigational treatment/medical device
    b. the clinical study/a study specific procedure
    c. other: e. g. concomitant treatment, underlying disease
11. Date of procedure
12. Reporter's name, date and signature

*In the case of incomplete information at the time of initial reporting, all appropriate information should be provided as soon as this becomes available.

UK only

The relationship of the SAE to the investigational treatment / medical device should be assessed by the investigator at site, as should the anticipated or unanticipated nature of any SAEs and SADEs.

All SAEs whether or not deemed investigational method/device related and whether anticipated or unanticipated must be reported to the Sponsor by email or fax within 24 hours (one working day) of the Investigator learning of its occurrence.

SAEs should be reported to:

Stephen Kelleher
Cambridge University Hospitals
NHS Foundation Trust
Box 277, Addenbrooke's Hospital
Hills Road, Cambridge, CB2 0QQ, UK
Phone: +44 (0) 1223 217418
Fax: +44 (0) 1223 348494
E-mail: enquiries@addenbrookes.nhs.uk

A written report must follow within five working days and is to include a full description of the event and sequelae, in the format detailed on the Serious Adverse Event reporting form. If applicable, the Sponsor will notify the competent authority of all Serious Adverse Events in line with pertinent legal requirements.

The Investigator will notify the Research Ethics Committee (REC) in UK of all Serious Adverse Events in line with pertinent legal requirements. The Investigator will inform the Sponsor about all reports sent to the reporting organisation including follow-up information and answers by the reporting organisation. The local investigator is responsible for informing other site principal investigators and the CI of all SAEs.

The regulatory authority (MHRA) will be notified of all SAEs as soon as possible within ten days of the event occurring during the study. The main REC will be notified of all unexpected and related SAEs within 15 days of the occurrence of the event.

Switzerland only (applicable to phase 1 only)

The following events are reported to the Representative of the Sponsor and Principal Investigator within 24 hours upon becoming aware of the event:

- All SAEs and SADEs
- Device deficiencies that might have potentially led to an SAE
- Health hazards that require measures

Dr Lia Bally
Department of Diabetes, Endocrinology, Clinical Nutrition and Metabolism
Inselspital, Bern University Hospital
University of Bern, Bern
Switzerland
Tel: 0041 31 632 36 77
Email: lia.bally@insel.ch

SAEs will evaluated by the Representative of the Sponsor and Principal Investigator with regard to causality and seriousness. Device deficiencies are assessed regarding their potential to lead to an SAE.

10.2.5 Recording and reporting of device deficiencies

All device deficiencies will be documented throughout the study. The investigator will be responsible for managing all device deficiencies and determine and document in writing whether they could have led to a serious adverse device effect.

All device deficiencies that might have led to a serious adverse device effect(s) if: suitable action had not been taken; intervention had not been made, or if circumstances had been less fortunate, must be reported to the Sponsor as for SAEs/SADEs.

10.2.6 Healthcare arrangements and compensation for adverse events

Healthcare arrangements for subjects who suffer an adverse event as a result of participating in the study may include advice from clinical members of the study team or the patient's treating diabetes team, or use of emergency health services.

If an adverse event occurs, there are no special compensation arrangements unless this was due to the negligence of one of the clinical investigators or due to harm resulting from study protocol design. In this case subjects may have grounds for legal action for compensation. The normal national complaints mechanism will be available. In addition, any harm arising due to study design (both negligent and non-negligent) will be covered under Sponsor's insurance policy as applicable.

10.2.6.1 Country specific requirements

1. UK - The Investigator will notify the ethics committee of all Serious Adverse Events in line with pertinent legal requirements. The Investigator will inform the Sponsor about all reports sent to the ethics committee including follow-up information and answers by the ethics committee. The MHRA and REC will be notified of all SUSARs occurring during the study according to the following timelines: fatal and life-threatening within 7 days of notification and non-life threatening within 15 days.

2. Switzerland (applicable to phase 1 only)- The Representative of the Sponsor and Principal Investigator reports SAEs and SADEs in Switzerland within 7 days to the local Ethics Committee which are
    - related or possibly related to the medical device under investigation
    - related or possibly related to study procedures

The Representative of the Sponsor and Principal Investigator reports the above both locally and internationally, within 7 days to Swissmedic. In addition, all device deficiencies that could have led to a SADE in Switzerland and study centres abroad will be notified to Swissmedic and the local Ethics Committee within 7 days (ClinO Rt: 42)

Health hazards that require measures are reported to the Ethics Committee and Swissmedic within 2 days (ClinO Art. 37).

An annual safety report including adverse events and device deficiencies including local and international events will be provided to Ethics Committee and Swissmedic every year by the Representative of the Sponsor (ClinO Art. 43).

The Representative of the Sponsor and Principal Investigator notifies Swissmedic and Ethics Committee of the completion of the study within 90 days (as of last patient, last visit). A discontinuation or an interruption of the trial, and the reasons for this are notified within 15 days. A final report, with contents in accordance with EN ISO 14155, will be submitted within one year of completion to Swissmedic and Local Ethics Committee (ClinO Art 38).

10.3 Anticipated adverse events, risks and benefits

10.3.1 Risks and anticipated adverse events

Known risks represent hazardous situations which may result in anticipated adverse events. In the following text, where appropriate, the term “risk” and “anticipated adverse events” are used interchangeably without affecting meaning.

10.3.2 Hypoglycaemia and hyperglycaemia

Subjects with type 2 diabetes requiring insulin therapy have a pre-existing risk for hypoglycaemia and hyperglycaemia. Potential risks are:

- Risk of mild to moderate hypoglycaemia and associated symptoms such as sweating, trembling, difficulty thinking and dizziness. There is also a rare risk of severe hypoglycaemia when conscious level is altered, needing help from a third party to correct the hypoglycaemia. These risks are pre-existent in any patient with type 2 diabetes requiring insulin and the study objective is to develop systems to minimise these risks

- Risk of possible mild to moderate hyperglycaemia similar to the risk that a subject with type 2 diabetes requiring insulin experiences on a daily basis

10.3.3 Finger-stick blood glucose measurements

Finger-stick tests may produce pain and/or bruising at the site.

10.3.4 Insulin injection therapy

Potential risks associated with multiple daily injection therapy include:

- Slight discomfort at the time of insulin injection (common)
- Slight bruising at the site of injection (common)
- Bleeding at injection site (rare)
- Infection at the site of injection (rare)
- Insulin pen malfunction and mechanical problems (rare)
- Allergy to insulin (very rare)
- Lipodystrophy / lipoatrophy (very rare)

10.3.5 Insulin pump therapy

Potential risks associated with insulin pump therapy include:

- Slight discomfort at the time of insertion of the insulin delivery cannula (common)
- Slight bruising at the site of insertion (common)
- Bleeding at insertion site (rare)
- Infection at the site of insertion (rare)
- Allergy to the insulin delivery cannula or adhesive (rare)
- Infusion set and cannula occlusions (rare)
- Insulin pump malfunction and mechanical problems (rare)
- Allergy to insulin (very rare)
- Lipodystrophy / lipoatrophy (very rare)

10.3.6 Continuous glucose monitoring

Potential risks associated with CGM:

- Slight discomfort at the time of insertion of CGM (common)

- Slight bruising at the site of insertion (unlikely)
- Bleeding at insertion site (rare)
- Infection at the site of insertion (rare)
- Allergic reaction to the CGM sensor material (rare)

If a skin reaction is classified as severe (the observation is noticeable and bothersome to subject and may indicate infection or risk of infection or potentially life-threatening allergic reaction), an adverse event form will be completed.

10.3.7 Dialysis related events (applicable to phase 1 only)

Potential risks associated with haemodialysis include:

- Intra-dialytic hypotension
- Muscle cramps
- Cannulation difficulties
- Clotting of the dialysis circuit
- Infiltration of the haemodialysis access

10.3.8 Questionnaires

As part of the study, subjects will complete questionnaires which include questions about their private attitudes, feelings and behaviour related to diabetes. It is possible that some people may find these questionnaires to be mildly upsetting. Similar questionnaires have been used in previous research and these reactions are uncommon.

10.3.9 Risk Analysis and residual risk associated with the investigational device

After in-depth analysis and consideration of all the potential hazards in relation to use of the CamAPS HX system in the home environment, it is concluded that the CamAPS HX system is safe, if used as intended.

Risk Assessment of the CamAPS HX system has been carried out in accordance with ISO 14971:2012. A preliminary Hazard Determination has been carried out including consideration of the questions in Annex C of ISO 14971:2012.

Relevant to phase 1, one hazard ‘Hazard S16:“Unknown CGM accuracy in Infants/Children, Pregnant Women and those on dialysis treatment or with critical illness’ is the only hazard identified that could not be reduced to an acceptable risk level, post mitigation. Our in-detail risk/benefit assessment concluded that the benefits of the system outweigh the risk with respect to this specific hazard.

As per our risk management process, further risk analysis shall be undertaken post production and release as to ensure any issues raised are acted upon to ensure the CamAPS HX system continues to improve and develop.

10.4 Benefits

It is expected that day and night closed loop system may have an important role in the management of diabetes in this patient group. The closed loop system may impact on the frequency of hypoglycaemia with suspected fewer low glucose levels with closed loop insulin delivery compared with usual care. In addition to this, higher blood glucose levels above target should be reduced with use of the closed loop algorithm. During the closed-loop period, participants will not need to self-administer insulin, which may facilitate diabetes management. Therefore, participation in this study is likely to be beneficial for study participants.

As this will be a proof-of-concept study, it is possible that subjects will not directly benefit from being a part of this study. However, it is also possible that the blood glucose information from the CGM devices along with the information about insulin dosing during day and night closed loop will be useful for subjects’ diabetes self-management.

10.5 Data Safety Monitoring Board (DSMB)

An independent Data Safety Monitoring Board (DSMB) will be informed of all serious adverse events and any unanticipated adverse device effects that occur during the study and will review compiled adverse event data at periodic intervals.

11 Methods and assessments

11.1 Procedures

11.1.1 Weight

Weight will be recorded at the study initiation visit at baseline and before and after each dialysis session for phase 1. Participants in phase 2 will have a weight recorded at the beginning of each treatment arm. Weight will be measured in kilograms using a calibrated electronic scale.

11.1.2 Blood Glucose Meter Data

The blood glucose meter will be downloaded periodically during the duration of the study.

11.2 Assessment for Safety

Subjects will receive education about treating hypo- and hyperglycaemia during closed loop insulin delivery. If the low glucose alert from the CGM becomes activated in closed-loop, a capillary glucose sample will also be measured. They will be advised to treat any finger stick capillary glucose level below 4 mmol/l with quick acting carbohydrate. Written guidelines in keeping with subjects' usual treatment guidelines will be provided for dealing with hypo- and hyperglycaemia.

11.3 Assessment for Efficacy

Continuous glucose monitoring during the study for 20 days (phase 1) or 8 weeks (phase 2) in each arm will be used in each participant to assess for efficacy.

Two continuous glucose monitoring systems (CGM) will be used throughout this study: a blinded CGM with retrospective sensor glucose data read out, and a real-time system providing a contemporaneous display of sensor readings.

Blinded CGM will be used throughout the control arm. Sensors will be returned to the research team thereafter. Secondary glucose endpoints as outlined in 10.1 will be based on glucose data derived from data captured during this 20 day or 8 week period (phases 1 and 2 respectively).

Real-time CGM will be applied during closed loop intervention. The control algorithm will use the real-time CGM's continuous stream of glucose data to control insulin titration.

11.4 Questionnaires

Quantitative data on health-related quality of life will be assessed using validated questionnaires. Participants will complete a series of questionnaires including an evaluation of their experience and views of the diabetes treatment received. It is estimated that participants will take 10-15 minutes to complete the questionnaires. All results will be evaluated at the end of the study.

Questionnaires will include:

- Problem Areas in Diabetes Questionnaire
- Hypoglycaemia Fear Survey (HFS)
- Closed-loop experience questionnaire

12 Study materials and products

12.1 Insulin

During the control intervention and wash out period, subcutaneous insulin therapy will be administered using CE-marked insulin pen devices as per usual clinical practice.

During closed-loop intervention, faster acting insulin aspart (Fiasp, Novo Nordisk, Copenhagen, Denmark) will be administered via an insulin pump as described below (see 12.3).

12.2 Multiple daily insulin injections during control intervention and wash out period

During the control intervention when multiple daily injection therapy will be applied, insulin will be administered using CE-marked insulin pen devices as per usual clinical practice.

12.3 Insulin pump

During day and night automated closed loop glucose control the Dana insulin pump (SOOIL) or similar CE-marked insulin pump will be used.

12.4 Continuous subcutaneous glucose monitor

The Dexcom G6 real-time sensor with sensor applicator (Dexcom, Northridge, CA, USA) will be the study CGM. The sensor will be calibrated according to manufacturer's instructions.

12.5 Blood Glucose Meter

Study participants will use their own approved glucose meter for self-monitoring of capillary blood glucose (SMBG) during the study. The capillary glucose meter readings may be used to calibrate the sensor according to manufacturer's instructions.

12.6 Computer-based algorithm

The Cambridge closed loop controller has been used safely and effectively in the closed loop studies in both children and adults with T1D (study REC Ref. 06/Q0108/350, REC Ref. 07/H0306/116, REC Ref. 08/H0304/75, REC Ref. 08/H0308/297, REC Ref. 09/H0306/44, REC Ref. 10/H0304/87, REC Ref. 12/EE/0155, REC Ref. 12/EE/0034, REC Ref. 12/EE/0424, REC Ref. 13/EE/0120, REC Ref. 13/WM/0498, REC Ref. 13/EE/0251, REC Ref. 13/EE/0321, REC Ref. 13/EE/0018, REC Red 15/EE/0324, REC ref 16/EE/0286, REC ref 16/EE/0380 and REC Ref 17/LO/0576).

13 Data Analysis

13.1 Primary Endpoint Analysis

The primary analysis will evaluate the between group difference in time spent in the target glucose range from 5.6 to 10.0 mmol/l for phase 1, or from 3.9 to 10.0 mmol/l for phase 2 based on CGM glucose levels during the 20 day (phase 1) or 8 week (phase 2) intervention periods.

Mean ± SD or summary statistics appropriate to the distribution will be reported for the primary endpoint over the defined period by treatment intervention (20 days for phase 1 and 8 weeks for phase 2). The treatment interventions will be compared using a linear mixed model. A 95% confidence interval will be reported for the difference between the interventions based on the linear mixed model.

Residual values will be examined for an approximate normal distribution. If values are highly skewed, then a transformation or non-parametric methods will be used instead. However, previous experience suggests that the primary endpoint will follow an approximately normal distribution.

A 5% significance level will be used to declare statistical significance for the primary comparison. A two-sided p-value will be reported.

The primary analysis will be a single statistical comparison of a single outcome measure. No formal corrections for multiple comparisons will be performed for the key or secondary analyses in phase 1. In phase 2 analysis of the primary endpoint and other key endpoints listed below, the familywise type I error rate (FWER) will be controlled at two-sided $\alpha = 0.05$. A gatekeeping strategy will be used, where the primary endpoint will be tested first and if passing the significance testing, other key endpoints will be tested in the order listed below using the fixed- sequence methods at $\alpha = 0.05$:

- Time spent in the target range (3.9 to 10.0 mmol/L)
- Time spent with glucose levels above 10.0 mmol/L
- Mean of glucose levels
- HbA1c
- Percent time spent with glucose levels below 3.9 mmol/L

This process continues iteratively moving to the next variable down on the list until a non-significant result ($p \geq 0.05$) is observed, or all five variables have been tested. If a non-significant result is encountered, then formal statistical hypothesis testing is terminated and any variables below on the list are not formally tested and analysis of these variables becomes exploratory.

The primary analysis will be performed on an intention-to-treat basis using the treatment group assigned by randomization.

A per-protocol analysis restricted to participants with a minimum of 60% CGM data during control period and 60% use of closed-loop during closed-loop period will be conducted for the primary endpoint.

Primary endpoint hypotheses

- Null Hypothesis: There is no difference in the true mean time spent in the target range (5.6 to 10.0 mmol/L for phase 1, or 3.9 to 10.0 mmol/L for phase 2) over the intervention period between the two treatment groups.
- Alternative Hypothesis: There is a nonzero difference in the true mean time spent in the target range over the intervention period between the two treatment groups.

13.2 Other Key Endpoints

For the following key endpoints will be assessed:

- Time spent above target glucose (10.0 mmol/l)
- Average of glucose levels

- HbA1c (phase 2 only)
- Time spent with glucose levels <3.9 mmol/l

13.3 Secondary Endpoints

CGM derived indices:

- Percentage time spent at glucose <5.6 mmol/l to quantify time spent below target (phase 1)
- Percentage time spent at glucose <3.0 mmol/l
- Percentage time spent at glucose > 16.7 mmol/l (phase 2 only)
- Percentage time spent at glucose > 20.0 mmol/l
- Standard deviation and coefficient of variation of glucose to quantify the glucose variability
- Number of hypoglycaemia events (glucose < 3.5mmol/l) (phase 1 only)

Insulin and Other Endpoints:

- Total amount of insulin delivered
- Average inter-dialytic weight gain (phase 1 only)

For all secondary endpoints, summary statistics appropriate to the distribution will be tabulated by treatment group. Analysis of key endpoint, and all secondary CGM and insulin endpoints will parallel the primary analysis. A transformation or non-parametric method will be applied to all highly skewed secondary endpoints.

Summary statistics for the following outcome metrics will also be tabulated separately for daytime (defined as 8am to less than 12am for phase 1 and 6am to less than 12am for phase 2) and night time (defined as 12am to less than 8am for phase 1 and 12am to less than 6am for phase 2) for the duration of the study. For phase 1, the same will also be performed for dialysis days and non-dialysis days:

- Percent time with glucose levels spent in the target range (5.6 to 10.0 mmol/L in phase 1, 3.9 to 10.0 mmol/L in phase 2)
- Mean of glucose levels
- Standard deviation of glucose levels
- Percent time with glucose levels below 3.9 mmol/L
- Total insulin dose

13.4 Safety Evaluation

For each of the following safety outcomes, mean ± SD or summary statistics appropriate to the distribution will be tabulated by treatment group:

- Number of subjects with any severe hypoglycaemia events
- Number of adverse events per subject
- Number of serious adverse events per subject

For purposes of analysis, a severe hypoglycaemic event will be defined as an event requiring assistance of another person actively to administer carbohydrate, glucagon, or other resuscitative actions. These episodes may be associated with sufficient neuroglycopaenia to induce seizure or coma. If plasma glucose measurements are not available during such an event, neurological recovery attributable to the restoration of plasma glucose to normal is considered sufficient evidence that the event was induced by a low plasma glucose concentration.

All of the above safety outcomes will be tabulated for all subjects (including dropouts and withdrawals), regardless of whether CGM data are available and irrespective of whether closed loop was operational. All adverse events will be listed for the entire study duration, including washout period.

For severe hypoglycaemia (if enough events), the event rates will be compared using a repeated measures Poisson regression model.

13.5 Utility Evaluation

The amount of CGM use will be tabulated for each treatment arm, in addition to the amount of closed loop system use in the CL arm. Summary statistics appropriate to the distribution and range will be reported for the percentage of time using the CGM over the intervention period for each treatment group. The same will be done for the percentage of time using the closed loop system in the CL arm. Tabulations of summary statistics will also be performed for the percentage of time spent using the closed-loop system while using the CGM in the CL arm.

13.6 Questionnaires

Descriptive tabulations of questionnaires will be carried out, and scores will be calculated using provided scaling and scoring tools as appropriate.

13.7 Evaluative periods

Where appropriate, sensor based measures will also be calculated for day and night-time periods (phase 1 and 2) and dialysis days and non-dialysis days (phase 1). The interval from 8.00 to 24:00 defines day-time period, 00:00 to 08:00 am defines the night-time period in phase 1. The interval from 6.00 to 24:00 defines day-time period, 00:00 to 06:00 am defines the night-time period in phase 2. The primary and secondary measures will be calculated from day 1 until the end of each study intervention.

13.8 Interim monitoring and analyses

No formal interim analysis will be performed.

13.9 Sample size and power calculations

This is an exploratory analysis involving 32 subjects with at least 48 hours of data for phase 1, and 24 subjects with at least 48 hours of data for phase 2. Previous studies in people with type 2 diabetes in hospital may not provide reliable information about the within group variability in this particular population including those requiring maintenance renal replacement therapy. No formal power calculations thus apply.

Allowing for 20% loss to follow up means we would need a total of 40 randomised participants for phase 1, and 30 randomised participants for phase 2.

14 Case Report Forms

The Case Report Form (CRF) is the printed, optical, or electronic document designed to record all the protocol required information to be reported to the Chief Investigator for each study participant.

CRFs will be completed in accordance with GCP and ISO 14551 Guidelines. Corrections to the CRF will be performed by striking through the incorrect entry and by writing the correct value next to the data that has been crossed out; each correction will be initialled and explained (if necessary) by the Investigator or the Investigator’s authorised staff.

If any amendments to the protocol or other study documents are made, CRFs will be reviewed to determine if an amendment to these forms is also necessary.

15 Data Management

Confidentiality of subject data shall be observed at all times during the study. Personal details for each subject taking part in the research study and linking them to a unique identification number will be held locally on a study screening log in the Trial Master File at the investigation centre. These details will not be revealed at any other stage during the study, and all results will remain anonymous (in Switzerland, data will be considered coded).

Case report forms (CRFs) will be used for recording anonymised (in Switzerland, data will be considered coded) study data. CRFs will be completed in accordance with GCP and ISO 15197: 2013 Guidelines. The study identification number will be used on CRF. Names and addresses will not be used.

Electronic data will be stored on password-protected computers. All paper records will be kept in locked filing cabinets, in a secure office at the investigation centre. Only members of the research team and collaborating institutions will have password access to the anonymised (in Switzerland, data will be considered coded) electronic data. Only members of the research teams will have access to the filing cabinet. All data will be stored for at least 15 years in line with the General Data Protection Regulation (GDPR) (EU) 2016/679. In case of withdrawal of participants, data that were obtained before will be further used anonymised (in Switzerland, data will be considered coded).

Direct access to the source data will be provided for monitoring, audits, REC review and regulatory authority inspections during and after the study. The fully anonymised data may be shared with third parties (EU or non-EU based) for the purposes of advancing management and treatment of diabetes.

Appropriate procedures agreed by the Chief Investigator and Clinical Principal Investigators will be put in place for data review, database cleaning and issuing and resolving data queries.

15.1 Further information on data management – Switzerland only (applies to phase 1 only)

15.1.1 Hardware and software

Data will be managed using a research cluster with three servers. The cluster provides enhanced availability, reliability and scalable performance. The servers are located in locked dedicated server rooms with restricted access.

Two dedicated virtual machines are installed for research data management with REDCap – one productive instance and one test instance. The servers are running on Microsoft Windows Server and using Apache HTTP Server.

Study data will be collected and managed using the REDCap (Research Electronic Data Capture) software. REDCap is a secure, web-based application designed to support data capture for research studies, providing 1) an intuitive interface for validated data entry; 2) audit trails for tracking data manipulation and export procedures; 3) automated export procedures for seamless data downloads to common statistical packages; and 4) procedures for importing data from external sources [https://projectredcap.org/resources/ citations/]. The REDCap tools are hosted at the Department of Anaesthesiology and Pain Medicine, University Hospital Bern.

15.1.2 Data security, access and backup

The servers are behind a firewall and cannot be accessed through the internet. Furthermore, Apache HTTP Server and REDCap were configured to run under Secure Sockets Layer (SSL) hence data is being encrypted and is transmitted securely.

Available disk space is monitored actively. If free disk space is less than 10 percent then administrators get an email and more storage capacity will be added accordingly.

The application has a group and role-based security model. Each user belongs to one or more security groups with specific sets of permissions in relation to folder or projects in the system. Only dedicated site administrators have access to the admin console enabling user management and changing security settings.

The system can only be accessed entering a user name and password. All events are recorded in the user event list of the audit log files.

All servers are regularly backed up on storage servers in a separate server room using a multi-level system.

15.1.3 Analysis and archiving

REDCap provides data analysis by integrated tools for creating reports and charts.

All data can be exported in different formats (Microsoft Excel, CSV, PDF, SAS, Stata, R, SPSS) suitable for transfer to a statistical software package of choice. All data will be archived and secured in the database as long as required by legislation.

16 Ethics

The study will be conducted in accordance with the Declaration of Helsinki Ethical Principles for Medical Research involving Human Subjects (64th WMA General Assembly, Fortaleza, Brazil, October 2013).

16.1 Research Ethics Committee and Institutional Review Board

Prior to commencement of the study, the protocol, any amendments, subject information and informed consent and assent forms, any other written information to be provided to the subject, subject recruitment procedures, current investigator CVs, and any other documents as required by the Research Ethics Committee (REC) or Institutional Review Board will be submitted. Written approval will be obtained from the REC prior to the commencement of the study. Any additional requirements imposed by the REC or regulatory authority shall be followed.

16.2 Informed consent of study subjects

In obtaining and documenting informed consent, the investigator will comply with the applicable regulatory requirements and will adhere to GCP standards and to the ethical principles that have their origin in the Declaration of Helsinki. Prior to the start of the study, the Investigator will obtain favourable ethical opinion of the written informed consent form and any other written information to be provided to subjects.

Subjects will be given full verbal and written information regarding the objectives and procedures of the study and the possible risks involved. The study team will avoid any coercion or undue improper inducement of the subject to participate and subjects will be given ample time to consider participation in the study.

All subjects will have the right to leave the study at any time, without stating any reason, and without any negative consequences to their subsequent medical treatment. The subject will be informed in

a timely manner should any new information become available during the course of the study that may affect their well-being, safety and willingness to participate in the study.

Written consent will be obtained from participants according to REC requirements. The signed informed consent forms will be photocopied, originals filed in the Investigator’s Site File, a copy placed in the patient’s notes and a copy given to the subjects. All subjects will receive a copy of the informed consent form, and the Project Coordinator’s office will hold copies of the consent forms and Ethics Committee approvals and make them available upon request.

17 Amendments to the protocol

Any substantial amendments to the protocol and other documents shall be notified to, and approved by, the Research Ethics Committee or Institutional Review Board, and the regulatory authority, prior to implementation as per nationally agreed guidelines.

18 Deviations from the protocol

Deviations from the protocol should not occur without prior approval of the REC or sponsor except under emergency circumstances, to protect the rights, safety and well-being of subjects. If deviations do occur, they will be documented, stating the reason and the date, the action taken, and the impact for the subject and for the study. The documentation will be kept in the Investigator’s Site File.

Deviations affecting the subject’s rights, safety and well-being or the scientific integrity of the study will be reported to the REC and sponsor as soon as possible in a timely manner, following nationally-agreed guidelines.

19 Study management

19.1 Data and Safety Monitoring Board (DSMB)

An independent Data Safety Monitoring Board (DSMB) will comprise an independent chairperson and two external experts. The DSMB aims to safeguard the interests of trial participants, assess the

safety and efficacy of the interventions during the trial, and monitor the overall conduct of the clinical trial.

The DSMB should receive and review the progress and accruing data of the project clinical trials and provide advice on the conduct of the trial. The DSMB will be informed of all serious adverse events and any unanticipated adverse device effects that occur during the study and will review compiled adverse event data at periodic intervals.

19.2 Trial Steering Committee (TSC)

A trial steering committee (TSC) will supervise the trial, to ensure it is conducted to high standards in accordance with the protocol, the principles of GCP, and with regard to participant safety. This committee will consist of the Chief Investigator and Clinical Investigators.

The TSC will meet (in person or conference call) at regular intervals during active phase, and at the conclusion of the study. The TSC will consider the study and relevant information from other sources, ensuring at all times that ethical considerations are met when recommending the continuation of the trial.

20 Responsibilities

20.1 Chief Investigator

The Chief Investigator (CI) is the person with overall responsibility for the research and all ethical applications will be submitted by the CI. The CI is accountable for the conduct of the study and will ensure that all study personnel are adequately qualified and informed about the protocol, any amendments to the protocol, the study treatments and procedures and their study related duties. The CI should maintain a list of appropriately qualified persons to whom he/she has delegated specified significant study-related duties.

20.2 Principal Clinical Investigator

The Principal Clinical Investigator will be responsible for the day-to-day conduct of the clinical aspects of the study.

21 Reports and Publications

Data will be submitted for publication in internationally peer-reviewed scientific journals; members of the investigator group will all be co-authors. The privacy of each subject and confidentiality of their information shall be preserved in reports and publication of data.

22 Timetable

Inclusion of the first subject in the study is planned to take place in April 2019 for phase 1, and in October 2020 for phase 2. Expected completion of the last subject for phase 1 is December 2020 and the planned completion of the Phase 1 Clinical Study Report is March 2021. For phase 2, expected completion of the last subject is December 2021 and the planned completion of the Clinical Study Report is March 2022

23 Retention of Study Documentation

Subject notes must be kept for the maximum time period as permitted by each relevant institution. Other source documents and the Investigator's Site File must be retained for at least 15 years, in line with the General Data Protection Regulation (GDPR) (EU) 2016/679. The Principal Investigator will archive the documentation pertaining to the study after completion or discontinuation of the study.

Switzerland only (applies to phase 1 only)

The Representative of the Sponsor and Principal Investigator will retain all data pertaining for a minimum of 10 years after completion or discontinuation of the trial (Art 45 ClinO of HRA). Access will be restricted to research team members, Swissmedic and Ethics Committee.

24 Indemnity statements

The clinical investigators are indemnified to cover negligent harm to patients participating in the study by their membership of medical defence organisations. Indemnity for any harm arising from the conduct of research will be provided according to local arrangements in respective centre.

1. Cambridge, UK - National Health Service indemnity cover will apply for any claims arising from management and conduct of research. Any liability arising from study design will be covered by the clinical trial insurance policy organised by the University of Cambridge.

2. Bern, Switzerland - (applies to phase 1 only) Study insurance will be provided by the University Hospital of Bern. A copy of the certificate will be filed in the investigator site file and the trial master file.

25 References

1. American Diabetes Association. Diagnosis and classification of diabetes mellitus. Diabetes Care. 2010;33 Suppl 1:S62-9.

2. Marín-Peñalver JJ, Martín-Timón I, Sevillano-Collantes C, Del Cañizo-Gómez FJ. Update on the treatment of type 2 diabetes mellitus. World J Diabetes. 2016;7(17):354-95.

3. Lascar N, Brown J, Pattison H, Barnett AH, Bailey CJ, Bellary S. Type 2 diabetes in adolescents and young adults. Lancet Diabetes Endocrinol. 2018;6(1):69-80.

4. International Diabetes Federation. IDF Diabetes Atlas, 9th edn. Brussels, Belgium: 2019. Available at: https://www.diabetesatlas.org.

5. Skyler JS, Bergenstal R, Bonow RO, Buse J, Deedwania P, Gale EA, et al. Intensive glycemic control and the prevention of cardiovascular events: implications of the ACCORD, ADVANCE, and VA diabetes trials: a position statement of the American Diabetes Association and a scientific statement of the American College of Cardiology Foundation and the American Heart Association. Diabetes Care. 2009;32(1):187-92.

6. Registry TRAUR. 19th Annual Report of the Renal Association. NEPHRON 2017;137 (suppl1); 2017.

7. Orchard TJ, Temprosa M, Barrett-Connor E, Fowler SE, Goldberg RB, Mather KJ, et al. Long-term effects of the Diabetes Prevention Program interventions on cardiovascular risk factors: a report from the DPP Outcomes Study. Diabet Med. 2013;30(1):46-55.

8. Cahn A, Miccoli R, Dardano A, Del Prato S. New forms of insulin and insulin therapies for the treatment of type 2 diabetes. Lancet Diabetes Endocrinol. 2015;3(8):638-52.

9. Wright A, Burden AC, Paisey RB, Cull CA, Holman RR, Group UKPDS. Sulfonylurea inadequacy: efficacy of addition of insulin over 6 years in patients with type 2 diabetes in the U.K. Prospective Diabetes Study (UKPDS 57). Diabetes Care. 2002;25(2):330-6.

10. Rys P, Wojciechowski P, Rogoz-Sitek A, Niesyczyński G, Lis J, Syta A, et al. Systematic review and meta-analysis of randomized clinical trials comparing efficacy and safety outcomes of insulin glargine with NPH insulin, premixed insulin preparations or with insulin detemir in type 2 diabetes mellitus. Acta Diabetol. 2015;52(4):649-62.

11. Rabkin R, Simon NM, Steiner S, Colwell JA. Effect of renal disease on renal uptake and excretion of insulin in man. The New England journal of medicine. 1970;282(4):182-7.

12. Mak RH, DeFronzo RA. Glucose and insulin metabolism in uremia. Nephron. 1992;61(4):377-82.

13. Kobayashi S, Maejima S, Ikeda T, Nagase M. Impact of dialysis therapy on insulin resistance in end-stage renal disease: comparison of haemodialysis and continuous ambulatory peritoneal dialysis. Nephrology, dialysis, transplantation : official publication of the European Dialysis and Transplant Association - European Renal Association. 2000;15(1):65-70.

14. Kazempour-Ardebili S, Lecamwasam VL, Dassanyake T, Frankel AH, Tam FW, Dornhorst A, et al. Assessing glycemic control in maintenance hemodialysis patients with type 2 diabetes. Diabetes care. 2009;32(7):1137-42.

15. Riveline JP, Teynie J, Belmouaz S, Franc S, Dardari D, Bauwens M, et al. Glycaemic control in type 2 diabetic patients on chronic haemodialysis: use of a continuous glucose monitoring system. Nephrology, dialysis, transplantation : official publication of the European Dialysis and Transplant Association - European Renal Association. 2009;24(9):2866-71.

16. Rodbard D. Continuous Glucose Monitoring: A Review of Recent Studies Demonstrating Improved Glycemic Outcomes. Diabetes Technology & Therapeutics. 2017;19(Suppl 3):S-25-S-37.

17. Agarwal R, Light RP. Relationship between glycosylated hemoglobin and blood glucose during progression of chronic kidney disease. American journal of nephrology. 2011;34(1):32-41.

18. Stratton IM, Adler AI, Neil HA, Matthews DR, Manley SE, Cull CA, et al. Association of glycaemia with macrovascular and microvascular complications of type 2 diabetes (UKPDS 35): prospective observational study. BMJ (Clinical research ed). 2000;321(7258):405-12.

19. Oomichi T, Emoto M, Tabata T, Morioka T, Tsujimoto Y, Tahara H, et al. Impact of glycemic control on survival of diabetic patients on chronic regular hemodialysis: a 7-year observational study. Diabetes care. 2006;29(7):1496-500.

20. Morioka T, Emoto M, Tabata T, Shoji T, Tahara H, Kishimoto H, et al. Glycemic control is a predictor of survival for diabetic patients on hemodialysis. Diabetes care. 2001;24(5):909-13.

21. Williams ME, Lacson E, Jr., Teng M, Ofsthun N, Lazarus JM. Hemodialyzed type I and type II diabetic patients in the US: Characteristics, glycemic control, and survival. Kidney international. 2006;70(8):1503-9.

22. Kalantar-Zadeh K, Kopple JD, Regidor DL, Jing J, Shinaberger CS, Aronovitz J, et al. A1C and survival in maintenance hemodialysis patients. Diabetes care. 2007;30(5):1049-55.

23. Freedman BI, Andries L, Shihabi ZK, Rocco MV, Byers JR, Cardona CY, et al. Glycated albumin and risk of death and hospitalizations in diabetic dialysis patients. Clinical journal of the American Society of Nephrology : CJASN. 2011;6(7):1635-43.

24. Ramirez SP, McCullough KP, Thumma JR, Nelson RG, Morgenstern H, Gillespie BW, et al. Hemoglobin A(1c) levels and mortality in the diabetic hemodialysis population: findings from the Dialysis Outcomes and Practice Patterns Study (DOPPS). Diabetes care. 2012;35(12):2527-32.

25. Finfer S, Chittock DR, Su SY, Blair D, Foster D, Dhingra V, et al. Intensive versus conventional glucose control in critically ill patients. The New England journal of medicine. 2009;360(13):1283-97.

26. Moen MF, Zhan M, Hsu VD, Walker LD, Einhorn LM, Seliger SL, et al. Frequency of hypoglycemia and its significance in chronic kidney disease. Clinical journal of the American Society of Nephrology : CJASN. 2009;4(6):1121-7.

27. Galindo RJ, Hurtado CR, Pasquel FJ, Vellanki P, Umpierrez GE. Clinical Outcomes and Mortality among Patients Hospitalized with Hypoglycemia and End-Stage Renal Disease in the U.S. Diabetes. 2018;67(Supplement 1).

28. Freedman BI, Shenoy RN, Planer JA, Clay KD, Shihabi ZK, Burkart JM, et al. Comparison of glycated albumin and hemoglobin A1c concentrations in diabetic subjects on peritoneal and hemodialysis. Perit Dial Int. 2010;30(1):72-9.

29. Freedman BI, Shihabi ZK, Andries L, Cardona CY, Peacock TP, Byers JR, et al. Relationship between assays of glycemia in diabetic subjects with advanced chronic kidney disease. Am J Nephrol. 2010;31(5):375-9.

30. Peacock TP, Shihabi ZK, Bleyer AJ, Dolbare EL, Byers JR, Knovich MA, et al. Comparison of glycated albumin and hemoglobin A(1c) levels in diabetic subjects on hemodialysis. Kidney Int. 2008;73(9):1062-8.

31. NICE guideline [NG 28] Type 2 diabetes in adults: management.: National Institute for Health and Care Excellence; 2015 Dec 2015.

32. Approaches to Glycemic Treatment. Diabetes care. 2016;39(Supplement 1):S52.

33. National Kidney Foundation. KDOQI Clinical Practice Guideline for Diabetes and CKD: 2012 Update. 2012.

34. Care JBDSfI. Management of adults with diabetes on the haemodialysis unit. 2016.

35. Association AoBCDaTR. Managing hyperglycaemia in patients with diabetes and diabetic nephropathy-chronic kidney disease. 2018.

36. Holman RR, Paul SK, Bethel MA, Matthews DR, Neil HA. 10-year follow-up of intensive glucose control in type 2 diabetes. N Engl J Med. 2008;359(15):1577-89.

37. Edridge CL, Dunkley AJ, Bodicoat DH, Rose TC, Gray LJ, Davies MJ, et al. Prevalence and Incidence of Hypoglycaemia in 532,542 People with Type 2 Diabetes on Oral Therapies and Insulin: A Systematic Review and Meta-Analysis of Population Based Studies. PLoS One. 2015;10(6):e0126427.

38. Phillip M, Danne T, Shalitin S, Buckingham B, Laffel L, Tamborlane W, et al. Use of continuous glucose monitoring in children and adolescents (*). Pediatr Diabetes. 2012;13(3):215-28.

39. Kordonouri O, Hartmann R, Pankowska E, Rami B, Kapellen T, Coutant R, et al. Sensor augmented pump therapy from onset of type 1 diabetes: late follow-up results of the Pediatric Onset Study. Pediatr Diabetes. 2012;13(7):515-8.

40. Bergenstal RM, Klonoff DC, Garg SK, Bode BW, Meredith M, Slover RH, et al. Threshold-based insulin-pump interruption for reduction of hypoglycemia. N Engl J Med. 2013;369(3):224-32.

41. Ly TT, Nicholas JA, Retterath A, Lim EM, Davis EA, Jones TW. Effect of sensor-augmented insulin pump therapy and automated insulin suspension vs standard insulin pump therapy on hypoglycemia in patients with type 1 diabetes: a randomized clinical trial. JAMA. 2013;310(12):1240-7.

42. Hovorka R. Closed-loop insulin delivery: from bench to clinical practice. Nature reviews Endocrinology. 2011;7(7):385-95.

43. Hovorka R, Allen JM, Elleri D, Chassin LJ, Harris J, Xing D, et al. Manual closed-loop insulin delivery in children and adolescents with type 1 diabetes: a phase 2 randomised crossover trial. Lancet. 2010;375(9716):743-51.

44. Elleri D, Allen JM, Kumareswaran K, Leelarathna L, Nodale M, Caldwell K, et al. Closed-loop basal insulin delivery over 36 hours in adolescents with type 1 diabetes: randomized clinical trial. Diabetes care. 2013;36(4):838-44.

45. Nimri R, Danne T, Kordonouri O, Atlas E, Bratina N, Biester T, et al. The "Glucositter" overnight automated closed loop system for type 1 diabetes: a randomized crossover trial. Pediatric diabetes. 2013;14(3):159-67.

46. Hovorka R, Elleri D, Thabit H, Allen JM, Leelarathna L, El-Khairi R, et al. Overnight closed loop insulin delivery in young people with type 1 diabetes: A free-living randomised clinical trial. Diabetes Care. 2014;37(5):1204-11.

47. Thabit H, Tauschmann M, Allen JM, Leelarathna L, Hartnell S, Wilinska ME, et al. Home Use of an Artificial Beta Cell in Type 1 Diabetes. N Engl J Med. 2015;373(22):2129-40.

48. Nimri R, Muller I, Atlas E, Miller S, Kordonouri O, Bratina N, et al. Night glucose control with MD-Logic artificial pancreas in home setting: a single blind, randomized crossover trial-interim analysis. Pediatric diabetes. 2014;15(2):91-9.

49. Tauschmann M, Allen JM, Wilinska ME, Thabit H, Stewart Z, Cheng P, et al. Day-and-Night Hybrid Closed-Loop Insulin Delivery in Adolescents With Type 1 Diabetes: A Free-Living, Randomized Clinical Trial. Diabetes Care. 2016;Jan 6 [Epub ahead of print].

50. Leelarathna L, Dellweg S, Mader JK, Allen JM, Benesch C, Doll W, et al. Day and night home closed-loop insulin delivery in adults with type 1 diabetes: three-center randomized crossover study. Diabetes Care. 2014;37(7):1931-7.

51. Thabit H, Lubina-Solomon A, Stadler M, Leelarathna L, Walkinshaw E, Pernet A, et al. Home use of closed-loop insulin delivery for overnight glucose control in adults with type 1 diabetes: a 4-week, multicentre, randomised crossover study. Lancet Diabetes Endocrinol. 2014;2(9):701-9.

52. Thabit H, Hartnell S, Allen JM, Lake A, Wilinska ME, Ruan Y, et al. Closed-loop insulin delivery in inpatients with type 2 diabetes: a randomised, parallel-group trial. The lancet Diabetes & endocrinology. 2017;5(2):117-24.

53. Bally L, Thabit H, Hartnell S, Andereggen E, Ruan Y, Wilinska ME, et al. Closed-Loop Insulin Delivery for Glycemic Control in Noncritical Care. New England Journal of Medicine. 2018;379(6):547-56.

54. Elleri D, Allen JM, Tauschmann M, El-Khairi R, Benitez-Aguirre P, Acerini CL, et al. Feasibility of overnight closed-loop therapy in young children with type 1 diabetes aged 3-6 years: comparison between diluted and standard insulin strength. BMJ Open Diabetes Res Care. 2014;2(1):e000040.

55. Thabit H, Tauschmann M, Allen JM, Leelarathna L, Hartnell S, Wilinska ME, et al. Home use of an artificial beta cell in type 1 diabetes. N Engl J Med. 2015;373(22):2129-40.

56. Tauschmann M, Allen JM, Wilinska ME, Thabit H, Stewart Z, Cheng P, et al. Day-and-Night Hybrid Closed-Loop Insulin Delivery in Adolescents With Type 1 Diabetes: A Free-Living, Randomized Clinical Trial. Diabetes care. 2016;39(7):1168-74.

57. Stewart ZA, Wilinska ME, Hartnell S, Temple RC, Rayman G, Stanley KP, et al. Closed-Loop Insulin Delivery during Pregnancy in Women with Type 1 Diabetes. The New England journal of medicine. 2016;375(7):644-54.

58. Hovorka R, Canonico V, Chassin LJ, Haueter U, Massi-Benedetti M, Orsini Federici M, et al. Nonlinear model predictive control of glucose concentration in subjects with type 1 diabetes. Physiol Meas. 2004;25(4):905-20.

59. Wilinska ME, Chassin LJ, Acerini CL, Allen JM, Dunger DB, Hovorka R. Simulation environment to evaluate closed-loop insulin delivery systems in type 1 diabetes. Journal of diabetes science and technology. 2010;4(1):132-44.

60. Wilinska ME, Budiman ES, Taub MB, Elleri D, Allen JM, Acerini CL, et al. Overnight closed-loop insulin delivery with model predictive control: assessment of hypoglycemia and hyperglycemia risk using simulation studies. J Diabetes Sci Technol. 2009;3(5):1109-20.

61. Hovorka R, Kumareswaran K, Harris J, Allen JM, Elleri D, Xing D, et al. Overnight closed loop insulin delivery (artificial pancreas) in adults with type 1 diabetes: crossover randomised controlled studies. Bmj. 2011;342:d1855.

62. Kumareswaran K, Thabit H, Leelarathna L, Caldwell K, Elleri D, Allen JM, et al. Feasibility of closed-loop insulin delivery in type 2 diabetes: a randomized controlled study. Diabetes care. 2014;37(5):1198-203.

63. Thabit H, Hartnell S, Allen JM, Lake A, Wilinska ME, Ruan Y, et al. Closed-loop insulin delivery in inpatients with type 2 diabetes: a randomised, parallel-group trial. Lancet Diabetes Endocrinol. 2016.

64. Blaha J, Kopecky P, Matias M, Hovorka R, Kunstyr J, Kotulak T, et al. Comparison of three protocols for tight glycemic control in cardiac surgery patients. Diabetes Care. 2009;32(5):757-61.

65. Amrein K, Ellmerer M, Hovorka R, Kachel N, Parcz D, Korsatko S, et al. Hospital glucose control: safe and reliable glycemic control using enhanced model predictive control algorithm in medical intensive care unit patients. Diabetes Technol Ther. 2010;12(5):405-12.

66. Takahashi G, Sato N, Matsumoto N, Shozushima T, Hoshikawa K, Akitomi S, et al. Preliminary study on glucose control with an artificial pancreas in postoperative sepsis patients. Eur Surg Res. 2011;47(1):32-8.

67. Bally L, Thabit H, Hartnell S, Andereggen E, Ruan Y, Wilinska ME, et al. Closed-Loop Insulin Delivery for Glycemic Control in Noncritical Care. The New England journal of medicine. 2018.

26 Document amendment history

Version number	Date	Amendment information
1.1	12 June 2019	1. Inselspital, Bern University Hospital logo added<br>2. Switzerland funder information added<br>3. Language requirements in Switzerland updated<br>4. Recruitment in Switzerland clarified<br>5. Requirement for women of reproductive age to take a pregnancy test and use contraception during the study in Switzerland<br>6. Country specific requirements in Switzerland regarding safety reporting and data management
2.0	04 September 2020	All amendments made to reflect the addition of phase 2 of this trial involving patients with type 2 diabetes not on dialysis.<br>1. Study title and short title amended to include details of phase 2<br>2. Protocol version updated<br>3. Addition of clinical investigator and study co-ordinator names<br>4. Change to title on protocol signature page<br>5. Change to title on site signature page<br>6. Clarification of length of study periods for phase 1 and 2 throughout the protocol<br>7. Table of contents updated with relevant changes<br>8. Four further abbreviations added to abbreviations page<br>9. Study synopsis amended to outline additional details for phase 2<br>10. Addition to summary information to include details and reasoning behind phase 2<br>11. Further background on type 2 diabetes added which is relevant to phase 1 and 2 (3.1, 3.2, 3.5, 3.7).<br>12. Amendment to rationale for present study (3.12) to reflect purpose of phase 2<br>13. Study design and study flow chart figure updated to include details of phase 2<br>14. Minor amendments to study participant details to clarify differences in phase 1 and 2

		15. Minor amendments to recruitment and consent information to include recruitment targets for phase 2<br>16. Study schedule updated to include details of study contacts and visits in phase 2. Two tables added to outline study activities in phase 2.<br>17. Addition of contact times for phase 2<br>18. Addition of HbA1c measurement to baseline visit and end of intervention visit for phase 2<br>19. Addition of glucose target parameter for primary endpoint in phase 2 (3.9 to 10mmol/L)<br>20. Clarification of exploratory endpoint to confirm inter-dialytic weight gain applies to phase 1 only.<br>21. Adverse events: clarification that reporting of adverse events in Switzerland and those related to dialysis is applicable to phase 1 only.<br>22. Addition of details for recording participants' weight at beginning of each treatment arm for phase 2<br>23. Amendments to data analysis/ endpoints to include percentage time spent at <3.9 for phase 2 and clarification that dialysis related endpoints are applicable to phase 1 only<br>24. Sample size of 24 and target recruitment of 30 participants for phase 2 added to sample size and power calculations (13.9)<br>25. Clarification that data management, retention of study documentation and indemnity statements in Switzerland is applicable to phase 1 only<br>26. Changes to timetable to reflect altered completion dates for phase 1 and proposed completion dates for phase 2<br>27. References updated accordingly
3.0	14 April 2021	1. Change to recruitment to include GP practices within the West Suffolk, Cambridge and Peterborough Clinical Commissioning Group (CCG)
4.0		1. Increase in HbA1c upper threshold in inclusion criteria from 11% (97mmol/mol) to 12% (108mmol/mol) in order to allow recruitment of people with HbA1c in higher ranges, as people with HbA1c values >11% are commonly seen in type 2 diabetes outpatient clinics. This was discussed at the Data Safety and Monitoring Board meeting (DSMB) and was felt to be a safe approach to widen recruitment.

5.0	4 February 2022	1. Modification of endpoints to include HbA1c, and statistics for phase 2 in line with statistical analysis plan.

An open-label, single-centre, randomised, 2-period cross-over study to assess the efficacy, safety and utility of fully closed-loop insulin delivery in comparison with standard care, in adults with type 2 diabetes

AP-Renal Phase 2 Study

Statistical Analysis Plan
Randomised Crossover Trial

Version: 1.0
Version date: 26th January 2022
Author: Aideen Daly
Protocol version: 4.0

Revision History

Version Number	Author	Approver	Effective Date	Study Stage
1.0	Aideen Daly	Charlotte Boughton	3/2/2022	Enrolment

Version Number	Revision Description

1. Overview

This is an open label, single-centre, randomised, 2 period, cross-over study to assess the efficacy, safety and utility of fully closed-loop (CL) insulin delivery in comparison with standard care in adults with type 2 diabetes. It is expected that approximately 30 subjects will be randomised and participate in the trial. All participants will receive both interventions and the order of these interventions will be randomised based on a 1:1 ratio. There will be no run-in period. Following randomisation, subjects will enter into two eight-week study periods and will test one intervention per study period. The two study periods will be separated by a 2-4-week washout period.

2. Statistical Hypotheses

- Null Hypothesis: There is no difference in the true mean time spent in the target range (3.9 to 10.0 mmol/L) over the eight-week period between the two treatment groups.
- Alternative Hypothesis: There is a nonzero difference in the true mean time spent in the target range over the eight-week period between the two treatment groups.

3. Sample Size:

The study is projected to randomise 30 subjects. As this is an exploratory analysis and previous studies in people with type 2 diabetes in hospital may not provide reliable information about the within group variability in this outpatient population, no formal power calculations thus apply. Allowing for a 20% loss to follow-up, we aim to randomise 30 participants in order to have 24 completed subjects.

4. Outcome Measures

4.1 Primary efficacy endpoint:

Percent time spent in the target range (3.9 to 10.0 mmol/L) over the eight-week intervention period.

4.2 Other key endpoints:

- Percent time spent with glucose levels above 10.0 mmol/L
- Mean of glucose levels
- HbA1c

- Percent time spent with glucose levels below 3.9 mmol/L

4.3 Secondary efficacy endpoints:

CGM Metrics

- Percent time spent with glucose levels below 3.0 mmol/L
- Percent time spent with glucose levels above 16.7 mmol/L
- Percent time spent with glucose levels above 20.0 mmol/L
- Standard deviation of glucose levels
- Coefficient of variation of glucose levels

Insulin Delivery:

- Total daily insulin dose

4.3.1 Calculation of CGM metrics

For the primary and all key and secondary CGM metrics, a single value will be calculated for each subject for each eight-week period by pooling all CGM readings between the treatment initiation visit up to eight weeks post initiation visit or the end of treatment visit, whichever comes first. All glucose sensor readings will be weighted equally in the pooled percentages regardless of how they distribute across weeks. Data will not be truncated due to protocol deviations.

Summary statistics for the following CGM outcome metrics will be tabulated separately for daytime (defined as 06.00 to 23:59) and night-time (defined as 00:00 to 05:59),

- Percent time with glucose levels spent in the target range (3.9 to 10.0 mmol/L)
- Mean of glucose levels
- Standard deviation of glucose levels
- Percent time with glucose levels below 3.9 mmol/L

4.4 Questionnaires

Expectations, attitudes and responses to diabetes, diabetes management and the closed-loop system will be assessed using the following questionnaires:

- Hypoglycaemia Fear Survey
- Problem Areas in Diabetes (PAID) scale

- Closed-loop participant experience questionnaire (Closed-loop period only)

4.5 Utility outcomes

- Percent time of usage of the closed-loop system
- Percent time with sensor glucose available

5. Analysis Cohort

- The primary analysis and all secondary analyses will be performed on an intention-to-treat basis with each day included in the treatment group assigned by randomisation.
- A per-protocol analysis restricted to randomized participants with a minimum of 60% of available CGM readings during the control period and 60% CL system use during the CL period will be conducted for the primary outcome.
- Safety outcomes will be reported for all enrolled participants, regardless of whether the study was completed.

6. Analysis of the Primary Efficacy Endpoint

6.1 Included Subjects

Only subjects with a minimum of 48 hours of CGM data in at least one study period will be included. No minimum amount of closed-loop system use is required. If a subject has more than 48 hours of data in period 1 and then drops out of the study without any data in period 2, then they will be included in the analysis.

6.2 Missing Data

Missing data will not be imputed for the primary analysis in this study

6.3 Statistical Methods:

Mean ± SD or summary statistics appropriate to the distribution will be reported for the primary outcome and each of the key secondary outcomes listed below over the eight week period by treatment intervention. The treatment interventions will be compared using a linear mixed model. Mean changes from baseline will be analysed using a restricted maximum likelihood (REML)-based repeated measures approach. Analyses will include the fixed

categorical effect of period. A common unstructured covariance structure will be used to model the within-patient errors. The Kenward-Roger approximation will be used to estimate denominator degrees of freedom. A 95% confidence interval will be reported for the difference between the interventions based on the linear mixed model. Residual values will be examined for an approximate normal distribution. If values are highly skewed, then a transformation or non-parametric methods will be used instead. However, previous experience suggests that the primary outcome will follow an approximately normal distribution. A separate model will also be built with the inclusion of a period by treatment interaction to assess for the presence of a carryover effect. We do not expect a carryover effect to be present.

Significance tests will be based on least-squares means using a two-sided $\alpha = .05$ (two-sided 95% confidence intervals). Analyses will be implemented using SPSS.

For the primary endpoint and other key endpoints listed in section 4, the familywise type I error rate (FWER) will be controlled at two-sided $\alpha = 0.05$. A gatekeeping strategy will be used, where the primary endpoint will be tested first and if passing the significance testing, other key endpoints will be tested in the order listed below using the fixed- sequence methods at α= 0.05:

- Time spent in the target range (3.9 to 10.0 mmol/L)
- Time spent with glucose levels above 10.0 mmol/L
- Mean of glucose levels
- HbA1c
- Percent time spent with glucose levels below 3.9 mmol/L

This process continues iteratively moving to the next variable down on the list until a non-significant result ($p \geq 0.05$) is observed, or all five variables have been tested. If a non-significant result is encountered, then formal statistical hypothesis testing is terminated and any variables below on the list are not formally tested and analysis of these variables becomes exploratory.

Regardless of the results of the hierarchical testing, summary statistics appropriate to the distribution will be tabulated by treatment arm for each hierarchical outcome. A 95% confidence interval for the treatment arm difference will also be calculated for all five hierarchical outcomes listed. However, a confidence interval that excludes zero will not be considered a statistically significant result if an outcome variable higher on the hierarchical list failed to reach statistical significance.

7. Analysis of the secondary endpoints

7.1 Included Subjects

For secondary CGM metrics, HbA1c and insulin metrics, inclusion criteria will be the same as the primary endpoint analysis. Subjects with a minimum of 48 hours of CGM data in at least one study period will be included in the primary analysis. No minimum amount of closed-loop system use is required.

7.2 Missing Data

For secondary endpoints, missing data will not be imputed in this study.

7.3 Analysis Windows

HbA1c obtained within ±14 days of the end of treatment visit dates during each period will be included in the analyses as the outcome. Baseline HbA1c measurements must be within ±14 days of the recruitment visit.

7.4 Statistical Methods

7.4.1 Secondary CGM Outcomes:

For all secondary CGM outcomes, summary statistics appropriate to the distribution will be tabulated by treatment group over the eight-week period. Analysis of all secondary CGM endpoints will parallel the primary analysis. A transformation or non-parametric methods will be applied to all highly skewed secondary outcomes.

7.4.2 HbA1c

For HbA1c, the treatment interventions will be compared using a linear mixed model. Mean changes from baseline will be analysed using a restricted maximum likelihood (REML)-based repeated measures approach. The model will include three time points: (1) baseline, (2) period 1 outcome, and (3) period 2 outcome. Analyses will include the fixed, categorical effect of period. A common unstructured covariance structure will be used to model the within-patient errors. The Kenward-Roger approximation will be used to estimate denominator degrees of freedom. A 95% confidence interval will be reported for the difference between the interventions based on the linear mixed model. Residual values will be examined for an approximate normal distribution. If values are highly skewed, then a transformation or non-parametric methods will be used instead.

Significance tests will be based on least-squares means using a two-sided $\alpha = .05$ (two-sided 95% confidence intervals). The primary treatment comparison will be the contrast between treatments.

7.4.3 Secondary Insulin Outcomes

For all secondary insulin outcomes, summary statistics appropriate to the distribution will be tabulated by treatment group over the eight-week period. Analysis of insulin endpoints will parallel the primary analysis.

7.4.4 Secondary analyses by time of day

Summary statistics for the following outcome metrics will also be tabulated separately for daytime (defined as 06.00 to 23:59) and night-time (defined as 00:00 to 05:59),

- Percent time with glucose levels spent in the target range (3.9 to 10.0 mmol/L)
- Mean of glucose levels
- Standard deviation of glucose levels
- Percent time with glucose levels below 3.9 mmol/L
- Total insulin dose

The measures will be calculated from day 1 until the end of each study intervention.

7.5 Questionnaire analyses

For each questionnaire (and their corresponding subscales), summary statistics appropriate to the distribution will be reported for the mean scores at each time point. Overall scores (and mean scores for each subscale) will be compared between treatments using the same model described above for the primary outcome.

For all questionnaires, at least 75% of the questions must be answered in order to be included in the analyses. This rule will be applied separately for the overall score and for each individual subscale so it is possible that the sample size may be different for some subscales. The score used for the analysis will only be based on the questions that were answered. Analysis will be limited to subjects who submit a questionnaire (no imputation).

7.6 Utility analyses

The amount of CGM use will be tabulated for each treatment arm, in addition to the amount of closed-loop system use in the CL arm. Summary statistics appropriate to the distribution and range will be reported for the percentage of time using the CGM over the eight-week period for each treatment group. The same will be done for the percentage of time using the

closed-loop system in the CL arm. Tabulations of summary statistics will also be performed for the percentage of time using the closed-loop system while using the CGM in the CL arm.

The percentage of time spent using the CGM will be calculated by dividing the total number of CGM readings by the expected number of readings during the eight-week period. The percentage of time using the closed-loop system in the CL arm will be calculated by dividing the total amount of time that temporary basal infusion lasts no more than 30 minutes by the maximum possible amount of time that the system could have been used. The percentage of time using the closed-loop system while using the CGM (in the CL arm) will then be computed by dividing the time that the closed-loop system was operational by the amount of time that the CGM was available.

If a subject drops out of the study in the middle of a period, then the CGM or closed-loop system use will be calculated until the time of withdrawal.

8. Safety Analyses

8.1 Adverse events summary

All episodes of severe hypoglycaemia along with any other reportable adverse event will be listed by treatment group. Separate listings will be provided for pre-randomisation and post-randomisation adverse events.

8.2 Comparison of Safety outcomes between Treatment Groups

The following safety outcomes will be compared between treatment arms if there are enough events associated with each arm:

- Number of subjects with any severe hypoglycaemia events
- Number of adverse events per subject
- Number of serious adverse events per subject

For purposes of analysis, a severe hypoglycaemic event will be defined as an event requiring assistance of another person actively to administer carbohydrate, glucagon, or other resuscitative actions. These episodes may be associated with sufficient neuroglycopaenia to induce seizure or coma. If plasma glucose measurements are not available during such an event, neurological recovery attributable to the restoration of plasma glucose to normal is considered sufficient evidence that the event was induced by a low plasma glucose concentration.

All of the above safety outcomes will be tabulated for all subjects (including dropouts and withdrawals) and by treatment period regardless of whether CGM data are available or whether the closed-loop system was operational. All adverse events will be listed by

participant for each intervention arm for all events for the entire study duration, including washout period. Each period will inclusively consist of all days in between the treatment initiation visit and the end of treatment visit. If the subject drops out of the study in the middle of a period and the end of treatment visit for that particular period does not occur, then the dropout date will be used as the last day of the period. Any adverse events that occurred before the treatment initiation visit in period 1 or during the washout period will not be included in the rate calculations or treatment group comparisons.

For severe hypoglycaemia (if enough events), the event rates will be compared using a repeated measures Poisson regression model adjusting for period. Binary variables will also be compared using a repeated measures logistic regression model adjusting for period.

9. Adherence and retention analyses

The following tabulations and analyses will be performed to assess protocol adherence for the study:

- Number of protocol and procedural deviations per subject along with the number and percentage of subjects with each number of deviations
- Number of protocol and procedural deviations by severity with brief descriptions
- A flow chart accounting for the number of subjects enrolled, the number of dropouts pre- and post-randomisation, and the number of subjects eligible to be included in the primary analysis
- Number of and reasons for unscheduled visits.

10. Baseline Descriptive Statistics

Baseline demographic and clinical characteristics of the cohort of all randomised subjects will be summarised in a table. Descriptive statistics will be tabulated overall and by randomisation group. For continuous variables, summary statistics appropriate to the distribution will be given. For discrete variables, number and percentage will be reported for each category.

11. Planned Interim Analyses

No formal interim analyses are planned for this study. The DSMB will review data collected for the study every 4-6 months. The data to be reviewed will included information regarding the following:

- Status of randomised participants

- Recruitment rates by month
- Baseline demographics
- Dropped participants and reasons for discontinuation
- Reportable adverse events

12. Subgroup Analyses

No subgroup analyses are planned for this study

13. Multiple Comparisons

For the primary endpoint and other key endpoints listed in section 4, a gatekeeping strategy will be used, where the primary endpoint will be tested first and if passing the significance test, other key endpoints will be tested in turn using a fixed-sequence method at α= 0.05 (see section 6.3).

- Time spent with sensor glucose levels between 3.9 to 10.0 mmol/l
- Time spent above target glucose (10.0 mmol/l)
- Average of glucose levels
- HbA1c
- Time spent below target glucose (3.9 mmol/l)

For the secondary endpoints, Benjamini-Hochberg false discovery rate (FDR) adjusted p-values will be calculated within each category. The FDR will be calculated separately within each of the following categories:

- Secondary efficacy outcomes
- Questionnaires

14. Exploratory Analyses

14.1 CGM analyses by fortnight (two-weeks)

Each of the following CGM metrics will be calculated on a fortnightly level.

- Percent time with glucose levels spent in the target range (3.9 to 10.0 mmol/L)
- Mean of glucose levels
- Standard deviation of glucose levels
- Percent time with glucose levels below 3.9 mmol/L
- Total insulin dose

Fortnight level metrics will only be tabulated descriptively. Treatment arm comparisons will not be done for these metrics. We expect each subject to have eight weeks' worth of data. The following table displays the post-initiation days that will be included in the calculation of CGM metrics for each fortnight:

Fortnight	First Day Included	Last Day Included
1	1	14
2	15	28
3	29	42
4	43	56

If the end of treatment visit occurs prior to day 56, then the visit date will be the last day of the fortnight, and the subsequent fortnights will not include any CGM readings.
