# Supplementary material for: Fully automated closed-loop insulin delivery in adults with type 2 diabetes: an open-label, single-center, randomized crossover trial
Source: Nat Med. 2023 Jan 11;29(1):203–8. doi: 10.1038/s41591-022-02144-z (PMC9873557; doi:10.1038/s41591-022-02144-z)
Supplement: Supplementary file 2 — Reporting Summary [file 41591_2022_2144_MOESM2_ESM.pdf]

Corresponding author(s): Dr Charlotte Boughton

Last updated by author(s): Nov 22, 2022

Reporting Summary

Nature Research wishes to improve the reproducibility of the work that we publish. This form provides structure for consistency and transparency in reporting. For further information on Nature Research policies, see our Editorial Policies and the Editorial Policy Checklist.

Statistics

For all statistical analyses, confirm that the following items are present in the figure legend, table legend, main text, or Methods section.

n/a	Confirmed	
☐	☒	The exact sample size (*n*) for each experimental group/condition, given as a discrete number and unit of measurement
☐	☒	A statement on whether measurements were taken from distinct samples or whether the same sample was measured repeatedly
☐	☒	The statistical test(s) used AND whether they are one- or two-sided<br>*Only common tests should be described solely by name; describe more complex techniques in the Methods section.*
☒	☐	A description of all covariates tested
☐	☒	A description of any assumptions or corrections, such as tests of normality and adjustment for multiple comparisons
☐	☒	A full description of the statistical parameters including central tendency (e.g. means) or other basic estimates (e.g. regression coefficient) AND variation (e.g. standard deviation) or associated estimates of uncertainty (e.g. confidence intervals)
☐	☒	For null hypothesis testing, the test statistic (e.g. F, t, r) with confidence intervals, effect sizes, degrees of freedom and P value noted<br>*Give P values as exact values whenever suitable.*
☒	☐	For Bayesian analysis, information on the choice of priors and Markov chain Monte Carlo settings
☐	☒	For hierarchical and complex designs, identification of the appropriate level for tests and full reporting of outcomes
☐	☒	Estimates of effect sizes (e.g. Cohen's *d*, Pearson's *r*), indicating how they were calculated

Our web collection on statistics for biologists contains articles on many of the points above.

Software and code

Policy information about availability of computer code

Data collection	The CamAPS HX closed-loop app (CamDiab, Cambridge, UK) resides on an unlocked Android phone, receives sensor glucose data from a Dexcom G6 transmitter (Dexcom, San Diego, CA, USA), and uses Cambridge adaptive model predictive control algorithm (version 0.3.71) to direct insulin delivery on a Dana pump (Diabecare, Sooil, South Korea). Sensor glucose and insulin data were obtained from the Diasend/ Glooko (https://diasend.com//en) data management platform.
Data analysis	Outcomes were calculated using GStat software, version 2.3 (University of Cambridge), and statistical analyses were performed using SPSS, version 27 (IBM Software, Hampshire, UK).

For manuscripts utilizing custom algorithms or software that are central to the research but not yet described in published literature, software must be made available to editors and reviewers. We strongly encourage code deposition in a community repository (e.g. GitHub). See the Nature Research guidelines for submitting code & software for further information.

Data

Policy information about availability of data

All manuscripts must include a data availability statement. This statement should provide the following information, where applicable:

- Accession codes, unique identifiers, or web links for publicly available datasets
- A list of figures that have associated raw data
- A description of any restrictions on data availability

The data that support the findings of this study are available from the corresponding author for the purposes of advancing the management and treatment of diabetes. All data shared will be de-identified. Study protocol and statistical analysis plan are available with publication.

Field-specific reporting

Please select the one below that is the best fit for your research. If you are not sure, read the appropriate sections before making your selection.

☒ Life sciences ☐ Behavioural & social sciences ☐ Ecological, evolutionary & environmental sciences

For a reference copy of the document with all sections, see nature.com/documents/nr-reporting-summary-flat.pdf

Life sciences study design

All studies must disclose on these points even when the disclosure is negative.

Sample size

This was an exploratory study aiming for 24 completed subjects with at least 48 hours of data. Since previous studies using closed-loop in an inpatient setting and in outpatients requiring dialysis may not provide reliable information about the standard deviation of the primary endpoint in this particular population, no formal power calculation was applied. The sample size corresponds to the sample size of previous feasibility closed-loop randomised trials (Thabit et al. Lancet Diabetes & Endocrinol. 2017, Boughton et al. Lancet Diabetes & Endocrinol. 2019, Boughton et al. Nat Med. 2021).

Data exclusions

All analyses were carried out on an intention-to-treat basis. We included data from participants with at least 48 hours of sensor glucose data in at least one study period. Of 28 randomized participants, two participants withdrew prior to starting their first study period (both randomised to control therapy first). One participant stopped the study early during the first intervention period (closed-loop), due to difficulty managing devices. This participant completed a minimum of 48 hours therefore data for this period was included in the analysis.

Replication

Not replicated due to the nature of the study design: randomised crossover trial.

Randomization

Participants were randomised in a 1:1 ratio to an 8-week period of fully closed-loop glucose control with faster-acting insulin aspart (Fiasp) followed by an 8-week period of standard insulin therapy, or vice versa. Randomisation was performed using a web-based, permuted blocks-of-four, randomisation method to assign study participants to one of the two treatment sequences.

Blinding

Participants and investigators were not masked to the intervention being used during each period due to the nature of the interventions precluding the ability to mask.

Reporting for specific materials, systems and methods

We require information from authors about some types of materials, experimental systems and methods used in many studies. Here, indicate whether each material, system or method listed is relevant to your study. If you are not sure if a list item applies to your research, read the appropriate section before selecting a response.

Materials & experimental systems

n/a	Involved in the study	
☒	☐	Antibodies
☒	☐	Eukaryotic cell lines
☒	☐	Palaeontology and archaeology
☒	☐	Animals and other organisms
☐	☒	Human research participants
☐	☒	Clinical data
☒	☐	Dual use research of concern

Methods

n/a	Involved in the study	
☒	☐	ChIP-seq
☒	☐	Flow cytometry
☒	☐	MRI-based neuroimaging

Human research participants

Policy information about studies involving human research participants

Population characteristics

From 16th December 2020 to 24th November 2021, 30 participants were recruited and 28 randomised. Baseline characteristics of the study population (7 females and 19 males, mean±SD age 59±11years, diabetes duration 17.5±8.2years, baseline HbA1c 75±15mmol/mol [9.0±1.4%]), are shown in Table 1 and baseline diabetes regimen details are shown in Supplementary Table 1. Sex of participants was determined based on self-report.

Recruitment

Participants were recruited from diabetes outpatient clinics at Addenbrooke's Hospital in Cambridge, United Kingdom, and a primary care participant identification centre. Inclusion criteria comprised age 18 years and above with type 2 diabetes requiring subcutaneous insulin therapy and screening HbA1c ≤ 12% (108mmol/mol). Participants had to be literate in English, willing to perform regular finger-prick blood glucose monitoring and willing to wear study devices and follow study specific instructions. Exclusion criteria were type 1 diabetes, pregnancy or breast-feeding, severe visual or hearing impairment, allergy to insulin or the adhesive of plasters or serious skin disease affecting device placement, lack of reliable telephone facility for contact, alcohol abuse, Illicit or prescription drug abuse or any physical or psychological disease or use of medication(s) likely to interfere with the conduct of the trial or interpretation of the results. Sex and/or gender was not

considered in the study design.

Ethics oversight	The study protocol was approved by the local Research Ethics Committees (London – Stanmore Ethics Committee, UK) and Regulatory Authorities (MHRA). The safety aspects of the trial were overseen by an independent Data and Safety Monitoring Board.

Note that full information on the approval of the study protocol must also be provided in the manuscript.

Clinical data

Policy information about clinical studies

All manuscripts should comply with the ICMJE guidelines for publication of clinical research and a completed CONSORT checklist must be included with all submissions.

Clinical trial registration	The study is registered with ClinicalTrials.gov NCT04701424.
Study protocol	Study protocol is available with publication
Data collection	Participants were enrolled and randomised from 16th December 2020 to 24th November 2021. Participant demographics and medical history, body weight and height, glycated haemoglobin (HbA1c) and total daily insulin dose were recorded at enrolment. Participants were randomly assigned to either initial use of fully closed-loop glucose control with faster acting insulin aspart for 8 weeks followed by standard multiple daily insulin injection therapy for 8 weeks or vice versa. Glycaemic and insulin endpoints were collected from each 8 week study period. Psychosocial assessments were measured using questionnaires collected at the end of each study period.
Outcomes	The primary endpoint was the percentage of time the sensor glucose measurement was in the target glucose range of 3.9 to 10.0mmol/L based on sensor glucose levels during the 8-week study period.<br>Key endpoints included the percentage of time with sensor glucose above 10.0mmol/L, mean sensor glucose, HbA1c, and the percentage of time with sensor glucose below 3.9mmol/L. Secondary efficacy endpoints included time with sensor glucose below 3.0mmol/L, time with sensor glucose levels in hyperglycaemia (>16.7 and >20mmol/L), variability of glucose measured by standard deviation and the coefficient of variation of sensor glucose, and the total daily insulin dose.<br>Safety endpoints included severe hypoglycaemia (capillary glucose <2.2 mmol/L, or requiring assistance of another person), adverse events, serious adverse events and device deficiencies.<br>Utility evaluation included percentage time of sensor glucose availability, and percentage time of closed-loop operation. Psychosocial assessments were measured using questionnaires collected at the end of each study period.<br>Exploratory endpoints included analysis of a subset of sensor glucose metrics (time in target range, mean glucose, standard deviation and time with glucose <3.9mmol/L) over daytime (06:00 to 23:59) and night-time (00:00 to 05:59) periods, and on a fortnightly basis. There was no pre-specified analysis disaggregated by sex.
